# Dangers and Benefits of Social Media on E-Professionalism of Health Care Professionals: Scoping Review

**DOI:** 10.2196/25770

**Published:** 2021-11-17

**Authors:** Tea Vukušić Rukavina, Joško Viskić, Lovela Machala Poplašen, Danko Relić, Marko Marelić, Drazen Jokic, Kristijan Sedak

**Affiliations:** 1 Andrija Stampar School of Public Health School of Medicine University of Zagreb Zagreb Croatia; 2 Department of Fixed Prosthodontics School of Dental Medicine University of Zagreb Zagreb Croatia; 3 Department of Orthodontics School of Dental Medicine University of Zagreb Zagreb Croatia; 4 Department of Communication Sciences Catholic University of Croatia Zagreb Croatia

**Keywords:** e-professionalism, social media, internet, health care professionals, physicians, nurses, students, medicine, dental medicine, nursing

## Abstract

**Background:**

As we are witnessing the evolution of social media (SM) use worldwide among the general population, the popularity of SM has also been embraced by health care professionals (HCPs). In the context of SM evolution and exponential growth of users, this scoping review summarizes recent findings of the e-professionalism of HCPs.

**Objective:**

The purpose of this scoping review is to characterize the recent original peer-reviewed research studies published between November 1, 2014, to December 31, 2020, on e-professionalism of HCPs; to assess the quality of the methodologies and approaches used; to explore the impact of SM on e-professionalism of HCPs; to recognize the benefits and dangers of SM; and to provide insights to guide future research in this area.

**Methods:**

A search of the literature published from November 1, 2014, to December 31, 2020, was performed in January 2021 using 3 databases (PubMed, CINAHL, and Scopus). The searches were conducted using the following defined search terms: “professionalism” AND “social media” OR “social networks” OR “Internet” OR “Facebook” OR “Twitter” OR “Instagram” OR “TikTok.” The search strategy was limited to studies published in English. This scoping review follows the PRISMA-ScR (Preferred Reporting Items for Systematic Reviews and Meta-Analyses Extension for Scoping Reviews) guidelines.

**Results:**

Of the 1632 retrieved papers, a total of 88 studies were finally included in this review. Overall, the quality of the studies was satisfactory. Participants in the reviewed studies were from diverse health care professions. Medical health professionals were involved in about three-quarters of the studies. Three key benefits of SM on e-professionalism of HCPs were identified: (1) professional networking and collaboration, (2) professional education and training, and (3) patient education and health promotion. For the selected studies, there were five recognized dangers of SM on e-professionalism of HCPs: (1) loosening accountability, (2) compromising confidentiality, (3) blurred professional boundaries, (4) depiction of unprofessional behavior, and (5) legal issues and disciplinary consequences. This scoping review also recognizes recommendations for changes in educational curricula regarding e-professionalism as opportunities for improvement and barriers that influence HCPs use of SM in the context of e-professionalism.

**Conclusions:**

Findings in the reviewed studies indicate the existence of both benefits and dangers of SM on e-professionalism of HCPs. Even though there are some barriers recognized, this review has highlighted existing recommendations for including e-professionalism in the educational curricula of HCPs. Based on all evidence provided, this review provided new insights and guides for future research on this area. There is a clear need for robust research to investigate new emerging SM platforms, the efficiency of guidelines and educational interventions, and the specifics of each profession regarding their SM potential and use.

## Introduction

### Background

Global digital growth shows no sign of slowing, with a million new people worldwide coming online every day. This growth is clearly fueling social media (SM) use, as 45% of the world’s population are now SM users: a whopping 3.5 billion people [1]. The popularity and use of SM has increased substantially in the past few years, despite controversy around privacy, hacking, fake news, and all other negative aspects of online life [2].

Social media have been defined as “a group of online applications that allow for the creation and exchange of content generated by users” [3] and can be categorized into five groups: (1) collaborative projects (eg, Wikipedia), (2) blogs or microblogs (eg, Blogger or Twitter), (3) content communities (eg, YouTube), (4) social networking sites (SNSs; eg, Facebook), and (5) virtual gaming or social worlds (eg, Second Life) [4]. SNSs (eg, Facebook) are “applications that enable users to connect by creating personal information profiles, inviting friends and colleagues to have access to those profiles, and sending e-mails and instant messages between each other” [4]. There is a lot of mixing and confusion between the terms SM and SNSs with SM being a newer and a much broader term, encompassing SNSs.

As we are witnessing the evolution of SM use globally among the general population, popularity of SM has also been embraced by health care professionals (HCPs) [5]. It is further reflected as a considerable growth in the research about SM use in health and medicine [6], mainly focusing on the roles of SM or SNSs in linking patients and HCPs [7-9] or use of SM/SNSs for communication among HCPs [10].

Within these new platforms exists an unprecedented ability to expand access and communication, with the potential to revolutionize the way medical professionals interact with peers, patients, and the public. However, along with this expanded access lies the potential for inadvertent overlap between the physicians’ personal and professional lives. Boundary concerns are increasing with the blurring of personal and professional lines on SM [11]. Anything placed on the internet is essentially permanent, and our “digital footprint” stays forever documented in this virtual yet, for almost everyone, accessible world.

A definition for a new term “e-professionalism” was given by Cain and Romanelli [12] as “attitudes and behaviors (some of which may occur in private settings) reflecting traditional professionalism paradigms that are manifested through digital media.” The intersection between medical professionalism and SM has been termed also as online professionalism or digital professionalism [13].

Prior reviews have focused on the e-professionalism of medical students, residents, or physicians [14], or have presented a full spectrum of SM-related challenges and opportunities in the context of medical professionalism of diverse types of HCPs [15,16], but these studies were conducted almost 6 years ago. Within that time frame, the number of SM users (both HCPs and patients), SM influence on private and professional life, and new features within SM have increased substantially leaving scientific research struggling to keep up [17-19]. Since the end of November 2014, the number of Facebook users has doubled (from 1.35 billion to 2.7 billion) and the number of Instagram users almost quadrupled (from 300 million to 1.158 billion). New SM platforms like TikTok have gained popularity [20]. A gap has emerged in comprehensive understanding of the ways SM has influenced the medical field, especially professional behavior. With time being crucial in the context of SM evolution and exponential growth of users, this scoping review maps and summarizes recent findings and fills the knowledge gap about e-professionalism of HCPs.

### Objective

The purpose of this scoping literature review was to characterize the recent original peer-reviewed research studies published between November 1, 2014, to December 31, 2020, on e-professionalism of HCPs; to assess quality of the methodologies and approaches used; to explore the impact of SM on e-professionalism of HCPs; to recognize benefits and dangers of SM; and to provide insights to guide future research in this area.

## Methods

### A Scoping Review

We performed a scoping review to explore the extent of the latest current evidence on e-professionalism of HCPs. The review questions were what are the reported outcomes of the benefits and dangers, and of SM on e-professionalism for HCPs, and what was the quality of methodologies and approaches used on the use of SM affecting e-professionalism of HCPs? The review subquestions were which knowledge gaps have been identified in studies and what are the recommendations for future research?

The scoping review method was chosen to map and summarize the evidence, and inform future research in the domain of e-professionalism of HCPs [21-23]. We performed a scoping review consistent with the guidance provided by the Joanna Briggs Institute Reviewer Manual [24,25]. The scoping review follows the PRISMA-ScR (Preferred Reporting Items for Systematic Reviews and Meta-Analyses Extension for Scoping Reviews) guidelines [26]. The protocol was registered on the Open Science Framework (registration DOI 10.17605/OSF.IO/YR8TW) [27] on April 9, 2021.

### Search Strategy

A search of the literature was performed in January 2021 using 3 databases (PubMed, CINAHL [EBSCO], and Scopus). The searches were conducted for the period from November 1, 2014, to December 31, 2020, using the following defined search terms: “professionalism” (a Medical Subject Headings [MeSH] term) AND (“social media” [a MeSH term] OR “social networks” OR “Internet” [a MeSH term] OR “Facebook” OR “Twitter” OR “Instagram” OR “TikTok”) included in the title or abstract or keywords. The search strategy was limited to studies published in English.

The full search strategy is summarized in Multimedia Appendix 1. A senior information specialist validated the search strategy. For a comprehensive assessment, we also searched the reference lists of all the included articles to identify other studies that may be relevant to our review.

### Study Inclusion and Exclusion Criteria

Studies were included in this review if they were original research focused primarily on the use of SM among health professionals and studied the uses, benefits, or dangers of SM.

Studies were excluded from this review if they were not in English; were books, dissertations, reviews, reports, abstracts only, case studies, opinions, letters, commentaries, policies, guidelines, or recommendations; did not focus primarily on the use of SM among health professionals; did not study the uses, benefits, or dangers of SM among health professionals; did not study HCPs as a study population; described the use of SM primarily with a marketing or advertising focus; and were not available as full text in the final search.

### Eligibility and Data Extraction

Following the search, all references captured by the search engine were uploaded to the reference management system Zotero (Corporation for Digital Scholarship). Duplicates were identified and removed. Initial screening of the studies, based on the information contained in the titles and abstracts, was undertaken independently by 2 reviewers (TVR and JV). The interrater reliability (IRR) between them was established. IRR to screen the papers was determined using the indices *average pairwise percent agreement*, *Cohen kappa,* and *Krippendorff α* (alpha) [28]. IRR was calculated with the ReCal (“Reliability Calculator”), an online utility that computes IRR coefficients [29]. To assess the eligibility of the papers, two researchers (TVR and JV) independently reviewed and evaluated the studies. Discrepancies were discussed with reference to the research objectives until consensus was reached on the inclusion for the analysis.

Data extraction was done in two passes. In the first pass, seven reviewers (TVR, JV, DR, LMP, DJ, KS, MM) extracted the data from included studies; in the second pass, two reviewers (TVR and JV) agreed on the extracted data. The following data were extracted: authors, year, country of origin, study objective and design, data analysis methods, study population, type of SM, key measures, conclusions/recommendations, and ethics statements.

### Quality Evidence Assessment

The Quality Assessment Tool for Observational Cohort and Cross-Sectional Studies developed by the National Heart, Lung, and Blood Institute was used to evaluate the quality of the quantitative studies [30]. The Critical Appraisal Skills Programme appraisal tools were used to evaluate the quality of the qualitative and mixed methods reviewed studies [31]. Quality evidence assessment was performed by two reviewers (TVR and JV). If there was a discrepancy, a third reviewer was consulted (LMP).

### Ethics Approval and Consent to Participate

This was a scoping review with no data collected from human participants. Ethical approval was not needed.

## Results

### Findings

**Figure 1:**
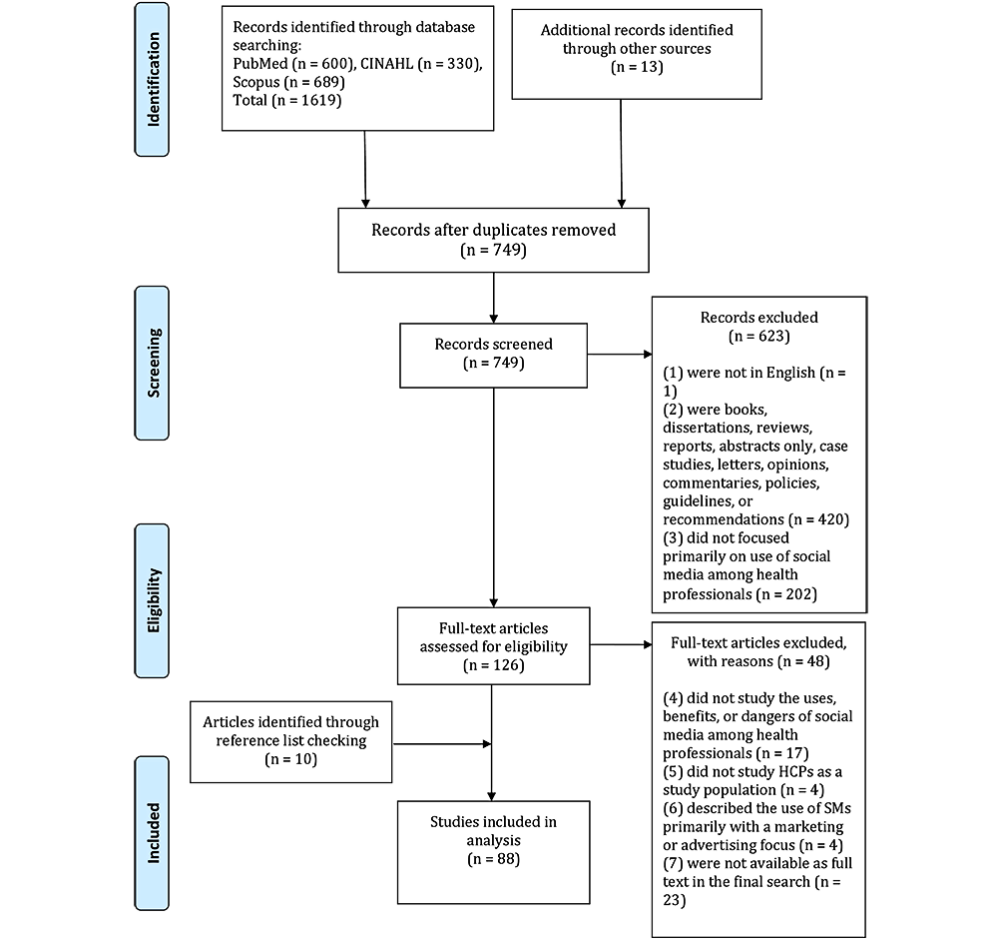
PRISMA (Preferred Reporting Items for Systematic Reviews and Meta-Analyses) flowchart of the selection procedure. HCP: health care professional; SM: social media.

Figure 1 shows the PRISMA flowchart illustrating the searching process and how the studies were included in the review. The literature search retrieved 1632 papers, and after removing duplicates, 749 titles and abstracts were screened. The IRR between the two researchers (TVR and JV) who screened titles and abstracts was established. IRR values indicated high reliability (average pairwise percent agreement 89%, Cohen kappa 0.80, Krippendorff α=.83). Full texts of 126 papers were assessed for eligibility. We also searched the reference lists of the included articles and found another 10 relevant articles for inclusion. Thus, a total of 88 studies were finally included in this review. Details of the studies including year, country of origin, study objective and design, data analysis methods, study population, type of SM, key measures, conclusions/recommendations, and ethics statements are shown in Multimedia Appendix 2. The studies (n=671) that were excluded are shown in Multimedia Appendix 3, along with the reasons for their exclusion.

### Characteristics of the Reviewed Studies

The studies were conducted in 40 countries, the majority being based in the United States (n=35), Canada (n=13), Australia (n=10), and the United Kingdom (n=10). Other countries with one or two studies were Brazil, China, Greece, Dominican Republic, Germany, Hong Kong, India, Ireland, Italy, Kingdom of Saudi Arabia, Mexico, New Zealand, Nigeria, Pakistan, Philippines, Singapore, South Africa, South Korea, Spain, Taiwan, Thailand, Turkey, and the United Arab Emirates (Multimedia Appendix 2). A total of 8 studies were conducted in several countries simultaneously [32-39]. Participants in the reviewed studies were from diverse health care professions (Table 1).

Medical health professionals were involved in about three-quarters of the studies. On several occasions, more than one health care profession was involved in the evaluated studies. Regarding the educational level of targeted HCPs, 35 studies investigated students [32,40-73]; 41 studies investigated residents or practicing HCPs [34-39,74-108]; 5 studies investigated deans or directors of programs [109-113]; and 7 studies investigated several educational levels of HCPs, students, residents, faculty members, or practicing HCPs [33,114-119].

Table 2 describes the types of SM/SNSs studied; the majority of the studies were unspecific, studying use of any type of SM or SNS. Only Facebook or “all SMs/SNSs with specific reference to Facebook” was analyzed in one-third of the studies (Multimedia Appendix 2). Twitter [38,44,80,91,110], Instagram [101], and YouTube [37] were specifically targeted SM/SNSs in 7 studies.

**Table 1:** Types of health care professions included in the reviewed studies.

Health care profession		Studies (N=88), n^a^
**Medical**		
	Deans and directors	2
	Faculty	6
	Specialists	11
	Doctors (general)	8
	Residents	15
	Students	23
**Dental**		
	Program directors	2
	Faculty	1
	Doctors (general)	1
	Students	5
**Nursing**		
	Deans and directors	1
	Faculty	2
	Nurses	3
	Students	8
**Pharmacy**		
	Pharmacists	2
	Students	4
Other health care professionals^b^		4

^a^A study could include more than one type of health care professional.

^b^Other health care professionals are physiotherapists, physician assistant students, and osteopathic medicine students.

**Table 2:** Types of social media or social networking sites.

Social media/social networking site	Studies, n
Unspecific (any type of social media/social networking sites)	59
Facebook	21
All social media sites with specific reference to Facebook	1
All social media sites with specific reference to Facebook and Twitter	3
Instagram	1
Twitter	2
YouTube	1

### Assessing the Quality of Studies

Overall, the quality of the studies was satisfactory. Most of the reviewed studies met the criteria in checklists (Multimedia Appendix 4). All studies were exploratory in nature, and the findings were descriptive. Among 88 studies, 49 were quantitative [32, 33, 35, 38-43, 51-53, 55, 59-61, 64, 66-69, 71, 72, 74, 75, 77-80, 82, 85, 88, 89, 91, 94-96, 98, 100, 102, 105-108, 110, 113, 114, 116, 118], 12 were qualitative [34, 36, 44, 45, 49, 57, 73, 76, 92, 93, 101, 104], and 27 used mixed methods [35, 37, 46-48, 50, 54, 56, 58, 62, 63, 65, 70, 81, 83, 84, 86, 87, 90, 97, 99, 103, 109, 111, 112, 115, 117]. Most studies used surveys (n=64) [32, 33, 35, 38-43, 47, 48, 51-55, 57, 59-62, 64-72, 74, 75, 77-82, 85, 88, 89, 91, 94-98, 100, 102, 103, 106-119]. The questionnaires used in surveys were mostly developed by researchers. Of these survey studies, only about one-third had a response rate of 50% or greater, and 9 studies did not explicitly report a response rate [39,53,60,63,65,67,72,75,96]. In the mixed methods studies, dominantly, content or thematic analyses were used. A total of 11 studies conducted in-depth or semistructured interviews [34,36,44,49,58,65,73,76,97,104,117], and 6 studies used focus groups [45,49,58,70,112,117]. Most studies included (n=77) had clear ethical statements within the paper either stating ethical board approval or exemption, and 11 studies did not explicitly report an ethical statement [37-39,53,54,72,74,90,105,108,117].

### SM Use Patterns Among Health Care Professionals

Studies that assessed SM use among different types of HCPs found high use among students, from 66.9% to 98.7% [40,43,50,51,60,61,64-66,68-70,102]; the highest with 98.7% using Facebook at least once a week was established among dental students. Lower rates of use were seen in practicing HCPs, physicians of different specialties, and program directors (PDs) or faculty, mostly ranging from 50% to 80% [35,39,74,80,83,85,86,88,89,91,96,105,106,108,110,113,114]. The exceptions were 3 studies: family medicine residents and physicians in Saudi Arabia where 95.4% of participants reported having an SM account and checking them at least once a day [79], 93.4% of medical doctors in a Singapore hospital [100], and 100% of Chinese registered nurses owned an SM account [107].

Several studies demonstrated a “generation gap” in SM use, where students are more likely than faculty to use SM [114,115,117,118]. A linear relationship between increasing age and decreasing SM use was also found among physicians of the same specialty or other HCPs [85,91,94,95,100,106].

Significant gender differences were established in several studies [71,79,91,95,105,107].

Irfan et al’s [79] study showed females using SM more for professional purposes, and Wang et al’s [107] study, where the study population was registered nurses, was similar. In Patel et al’s [91] study where a subgroup analysis on Twitter use for professional purposes revealed a significant gender difference: only one in four users was a female radiologist and only 14% of highly active users were female. Gender difference was established also in Gupta et al’s [62] content study of Facebook profiles (in favor of male medical students, ranging from 73.5% to 96.4%) [62], and 98.8% of all participants were males in a study about orthopedic surgeons [105].

Studies showed privacy settings deployment from 71% to 97% among HCPs [40,42,65,66,68,96,100,115,117,118]. Only 4 studies explicitly stated the percentage of students who had set their Facebook account on private: 37% of pharmacy students [40], 83.6% and 91% of dental students [64,65], and 71% of medical students [42]. More students than faculty used privacy features [115,118]. Results from a study among doctors in Singapore suggest that there is a knowledge deficit in terms of understanding of the privacy settings of SM accounts. From 30% to 55% of the respondents had an incorrect understanding of their SM account settings despite 95.5% claiming that they were aware that the institution had a SM policy [100]. Use of real names was investigated in 2 studies; in both, the vast majority of HCPs used their own names on Facebook [51,117], but on Twitter (45%) and Instagram (54%), far fewer dental students presented themselves with real names [51].

Studies also investigated the purpose of SM use, whether participants mixed professional and personal information and activities on SM sites (blended profiles) or adopted a separation strategy where professional information and activities were clearly separated from personal ones (dual citizenship) [34,39,69,78,79,91,96,97,106]. In Duke et al’s [115] study, significant differences were established between nursing students’ and faculties’ purpose of use, where almost twice as many students used SM for educational purposes than did faculty (58.5% vs 27.6%; *P*<.001), and almost 96% of students used SM to talk about academic-related problems compared to only 28% of faculty who did so (*P*<.001). Irfan et al [79] investigated family medicine residents and physicians use of SM not only for personal purposes (76%) or professional reasons (26%); they have determined that participants also use SM for general education (46%) and in a smaller proportion (18.2%) for continuing medical education [79]. A study among South African nurses established that they experience difficulty in separating their professional and personal lives when using SM [73].

In terms of themes covered in the reviewed studies, we have focused on two major themes: benefits and dangers of SM on e-professionalism of HCPs. This scoping review also recognizes an evaluation of effects of existing approaches on promoting e-professionalism and barriers that influence HCPs use of SM in the context of e-professionalism.

### Benefits of Social Media on E-Professionalism of Health Care Professionals

For the selected studies, there are three recognized benefits of SM on e-professionalism of HCPs: (1) professional networking and collaboration, (2) professional education and training, and (3) patient education and health promotion.

#### Professional Networking and Collaboration

The benefits of SM on e-professionalism of HCPs can be seen as improvements of established networks or possibilities for collaboration through SM sites [33, 34, 36, 38, 39, 44-46, 53, 69, 72, 76, 78, 79, 92, 97, 106-108, 117]. Besides providing the opportunity for connecting with others and sharing experiences [38,39,46,53,69,72,78,106-108], SM have enabled the creation of communities for support. This enables students to help each other in studying and interacting with faculty who can provide advice, encouragement, and virtual mentorship [44]. Chretien et al [44] describe two roles that medical students use via SM, first, as access to information and, second, as a voice, a platform for advocacy, and an opportunity to state attitudes and opinions. SM is also where they gained control of their digital footprint and a sense of equalization within the medical hierarchy. SM provide for students, residents, and faculty a good discussion medium and an engaging way to get high-quality current information [79,117]. Professional networking and collaborations on SM enable the development and building of professional identities for health care professions [34,38,39,45,67,73,104]. Some studies emphasize SM benefits of peer-to-peer advising or learning, provision of emotional support, and identifying approaches through which physicians establish interpersonal trust on SM [36,45,92].

#### Professional Education and Training

Several studies have demonstrated students’ use of SM for acquiring knowledge, to gain access to information from experts with whom they otherwise would not be able to connect with, or for creating communities that can then be used as means for supportive, professional, and social learning [44,45,58,66].

A survey among US surgeons indicated that 70% of respondents believe SM benefits professional development, similar to findings among Chinese urologist where 52.7% believe that SM provides a platform for surgical or medical education [95,106].

Canadian urologists (59%) consider SM as a simple information repository that is likely to increase in the continuing professional development space [95]. About 80% of HCPs from Saudi Arabia agreed with the benefits of using SM in health care services and considered that the use of these technologies in the provision of health services improves their professional knowledge and that SM can be a useful tool by which physicians may promote their services [75].

In Duke et al’s [115] study, significant differences were established between nursing students’ and faculties’ purpose of use, where almost twice as many students used SM for educational purposes than did faculty (58.5% vs 27.6%; *P*<.001), and almost 96% of students used SM to talk about academic-related problems compared to only 28% of faculty who did so (*P*<.001). Irfan et al [79] investigated family medicine residents’ and physicians’ use of SM for not only personal purposes (76.0%) or professional reasons (26.0%); they have determined that participants use SM also for general education (46.0%) and, in a smaller proportion, for continuing medical education (18.2%).

#### Patient Education and Health Promotion

Positive professional behaviors and attitudes regarding patient education and health promotion were also reported [34,37,75,97,106]. George et al [47] investigated US medical students’ attitudes about what positive role SM can play in improving communication with patients. A total of 44% of respondents stated they should and would react if a patient sought their medical advice via Facebook. Some students acknowledged the potential usefulness of SM in medical practice, patient education, health promotion, and interpersonal communication, if applied in a safe and responsible manner [47]. The thematic analysis of pharmacists’ semistructured interviews recognized addressing unprofessional posts made by peers as positive online behavior. Another positive professional activity was the use of SM to educate society in general about the role that pharmacists play in the health care system, their clinical roles, and how they can promote quality care for patients [34]. More than half of the HCPs in a cross-sectional study in Saudi Arabia agreed with the benefits of using SNSs in health care services as a suitable tool for patient education and raising public health awareness [75]. The results of the study among Saudi Arabian orthopedic surgeons showed that they are more likely to post online for the sake of sharing general medical knowledge as opposed to giving specific treatment advice. Most of them were open to the possibilities of using SM more with their patients for the sake of education, knowledge sharing, and improving patient outcomes [97]. In terms of communication with patients, in Long et al’s [106] study, the majority of urologists thought SM had improved efficiency in patient education (65.4%) and patient communication (55.1%).

### Dangers of Social Media on E-Professionalism of Health Care Professionals

For the selected studies, there are five recognized dangers of SM on e-professionalism of HCPs: (1) loosening accountability, (2) compromising confidentiality, (3) blurred professional boundaries, (4) depiction of unprofessional behavior, and (5) legal issues.

#### Loosening Accountability

According to some studies in this review, loosening accountability can be seen as a danger to e-professionalism from two points of view: eroding public trust by providing poor quality of information on SM [39,106,117] and damaging to the professional image [43, 45, 51, 56, 57, 59, 64, 66, 68, 70, 73, 102, 106, 112].

Potential damage to the professional image has been depicted by students as concerns about repercussions of their posts on career development or future employment, since employers are checking SM profiles of candidates [43, 45, 51, 56, 57, 59, 66, 68, 70].

Students are concerned about the extent of representation of the students’ character on SM; they edit profiles before interviews or career fairs [57,70] or intend to review or modify their profiles when they become qualified [45,51]. As students get closer to graduation, they are more concerned about future employment opportunities and their professional career. In addition, it has been reported that there is more awareness of online responsibilities as students progress through their program because employers can, and at times do, use SM profiles to make hiring decisions [56].

Three reviewed studies investigated PDs’ (medical and dental) attitudes about the use of SM for admission criteria [109,110,112]. In a study that evaluated how SM is being used in dental hygiene programs admissions and policy, only 4% of programs evaluated a potential student’s internet presence, mostly by searching on Facebook. Of those respondents that do not evaluate internet presence in applicants, more than half are not considering adding this to the admissions criteria (57.2%). Others are considering it (39.1%), and a small number (3.6%) plan to implement this in the future [109]. Use of SM is higher among medical PDs, and they more often view the online behavior of residency applicants, surgical residents, and faculty surgeons [110]. Among general surgery PDs, 18% reported visiting the SM profiles of medical students applying for surgical residency. Overall, 11% of PDs reported lowering the rank or completely removing a residency applicant from the rank order list because of online behavior [110].

#### Compromising Confidentiality

Being both an ethical and potentially legal issue, many of the studies have investigated attitudes toward compromising confidentiality, concerns that HCPs have about use of SM, patient privacy, and violations of Health Insurance Portability and Accountability Act (HIPAA) standards as a separate problem. Breaches of patient privacy was a concern for many different types of HCPs [34,43,47,57,91,94,97,107,111,119]. Bagley et al’s [41] results showed that the frequency of a student’s updates of a Facebook status appears to be associated with a risk of violating HIPAA online. Similar findings were made in Wejis et al’s [96] study. Greater disclosure on Facebook was associated with lesser awareness of the consequences of posting information on Facebook, a greater need for popularity, a higher level of self-esteem, a greater number of Facebook friends, and a higher frequency of signing in to Facebook [96]. In a study among nursing students, perceptions of confidentiality existed on the level of knowledge; all students knew that posting patient names or pictures was a breach of confidentiality. However, 34% were aware of other students who had breached patient confidentiality on Facebook [43]. “Cognitive dissonance,” a disconnect between what they thought they *would* do versus what they thought they *should* do was also reported by George et al [47].

Examples of compromising confidentiality and breaches of patient privacy were reported in several studies. In Long et al’s [106] study among Chinese urologists, nearly half of the respondents had experience posting information or pictures of patients’ SM, but only 5% of them sought their patients’ consent before posting [106]. In an exploratory qualitative study among nursing students in South Africa, students admitted that there is no responsible use of SM. They have stated that each of them perceives responsible use of SM differently. They took pictures, recorded video and audio clips of patients and of clinical interactions involving patients, and posted this information on SM compromising confidentiality [73]. In Wang et al’s [107] study, 13.4% of Chinese registered nurses (n=88) confessed that they had “sometimes” posted anonymous patient information on SM.

#### Blurred Professional Boundaries

Traditional boundaries are blurred on many levels by online interactions. Blurred boundaries between professional and personal spheres of SM use [34, 39, 47, 51, 69, 78, 79, 91, 96, 97, 106], with concerns about exposure of one’s private life or separating private and professional profiles have been presented in numerous studies of this review.

Several studies in this review investigated the purpose of SM use and whether participants mixed professional and personal information and activities on SM sites (blended profiles) or adopted a separation strategy where professional information and activities were clearly separated from personal ones (dual citizenship) [34,78,79,91,96].

Numerous studies document blurred boundaries between patient and HCPs, and between students and faculty [40, 43, 47, 59, 64, 65, 68, 70, 73, 75, 78, 82, 85, 98, 100, 105, 107, 114, 117]. Medical students have different attitudes regarding online interaction with a patient. “Friending a patient” is generally not acceptable nor endorsed; a wide range of opinions have been observed concerning this issue, ranging from one-third for medical students in Brazil [59] that find this unacceptable to 92% for senior medical students in New Zealand [70].

Among physicians, the majority have legal concerns about communicating with patients through SM [78,105,117]. In Fuoco and Leveridge’s [78] study about attitudes toward and use of SM among urologists, online patient interaction was endorsed by only 14% of urologists. Even though 56% of urologists agreed that SM integration in medical practice will be “impossible” due to privacy and boundary issues, 73% felt that online interaction with patients would become unavoidable in the future, especially for those in practice [78].

Students were anxious about the possibility their teachers could read about their personal life on SM. Dental students are ambivalent toward “friending” a faculty member [65]. From pharmacy students’ perspectives, an active user is generally open to “friending” the outside world. However, the majority were still reluctant to “friend” faculty members at their school. Students have beliefs that student-faculty interactions should remain professional, and SM sites are not appropriate venues for such professional communication [40]. Academic faculty members were worried that connecting via SM with students or residents would blur the boundaries of the teacher-student relationship. In Jafarey et al’s [117] study, almost half of faculty members found it inappropriate to friend a current student, and friending patients was not acceptable for 70% of respondents, with major differences found in age groups; it was acceptable to friend patients to 31% of trainees and 62% of students compared to only 5% of faculty. In a similar linear progression, younger age associated with more openness to being friends with patients was also demonstrated by Klee et al [82]. Two-thirds of family medicine residents and half of practicing physician respondents believed it was not ethical to be SM friends with patients.

Brisson et al [114] found that faculty were more likely than students to have been approached by patients on SNSs (53% vs 3%). Karveleas et al’s [64] study showed a significantly higher percentage of fifth-year dental students (48.3%) compared to fourth-year students (20.6%; *P*<.001) who had received a Facebook friend request from one or more patients [64].

#### Depiction of Unprofessional Behavior

Numerous studies in this review have tried to assess the extent of unprofessional behavior posted by HCPs themselves or seen to be posted by their peers. Although there is no uniform consensus on what constitutes unprofessional behavior, studies most frequently associated it with online content pertaining to alcohol intoxication; substance or illegal drug use, nudity, and sexuality; demeaning content about patients, peers, educators, clinical sites, or the profession as a whole; discriminatory content; profanity; and aggressive/bullying content toward coworkers. Surveys that captured students’ self-report of posted unprofessional behavior reported witnessing the investigated examples with varying frequencies [32, 42, 43, 55, 59, 62, 64, 73, 114, 118]. Among Brazilian medical students, frequencies ranged from 13.7% for “violation of patient’s privacy” to 85.4 % for “photos depicting consumption of alcoholic beverages” [59]. Posting of unprofessional content was highly prevalent among medical students in Australia despite understanding that this might be considered inappropriate and despite awareness of professionalism guidelines. A total of 34.7% of students reported unprofessional content (eg, evidence of being intoxicated 34.2%, illegal drug use 1.6%, posting patient information 1.6%, and depictions of an illegal act 1.1%) [36]. In Kenny and Johnson’s [51] study among dental students, 34% had questionable content on their profile, while 3% had definite violations of professionalism on their profile and 25% had unprofessional photographs on their profile including alcohol and different levels of nudity. Of those with unprofessional photographs, 52% had a documented affiliation with the dental school also visible on their profile [56]. In another study among dental students by Karveleas et al [64], unprofessional content had been posted by most students. A total of 71.7% of students had posted pictures from holidays, 41.5% moments in nightclubs, and 26.2% photographs wearing swimwear or underwear. Alcohol consumption and smoking were published by 19.1% and 5.5% of responders, respectively, while 0.4% of responders admitted having posted photographs of themselves using illegal drugs [64].

An international survey among health science students, from 8 universities in 7 countries, registered that a significant number of students (20.5%) across all health science disciplines self-reported sharing clinical images inappropriately [32]. Furthermore, medical students who observed unprofessional behaviors were more likely to participate in such behaviors [55], and the phenomenon of “distancing” was described among nursing students, while the existence of unwise posting on SNSs was widely acknowledged, students tended to attribute such behavior to others [43].

Age difference in the terms “older and wiser,” meaning more cautious about posting unprofessional behavior online, was proven in studies comparing students’ and faculties’ online behavior. Medical students were more likely than faculty to display content they would not want patients to see (57% vs 27%), report seeing inappropriate content on colleagues’ SNS profiles (64% vs 42%), and ignore harmful postings by colleagues (25% vs 7%) [114]. Medical students in Kitsis et al’s [118] study reported the self-posting of profanity, depiction of intoxication, and sexually suggestive material more often than faculty (*P*<.001). Medical students and faculty both reported peers posting unprofessional content significantly more often than self-posting [118].

Studies that assessed the online unprofessional behavior of residents or practicing HCPs were dominantly among different physicians’ specialties (emergency medicine [EM], public health professionals, surgical residents or practicing surgeons, urology residents or practicing urologists, different residencies/specialties) [77, 80, 83, 84, 86, 87, 89, 96, 99, 100, 119], with 1 study investigating nurses [107] and 1 investigating pharmacists [34].

Soares et al’s [119] study compared EM trainees’ and faculties’ perceptions of unprofessional SM behaviors to those of state medical board directors from a prior published study [120]. They found that themes involving patient information, inappropriate communication, and discriminatory speech elicited similar probabilities of anticipated investigation by both EM and state medical board directors, compared to published data. However, compared with state medical board directors, EM physicians were less likely to anticipate that themes involving alcohol and disrespectful speech would be investigated. A study to assess changes in unprofessional content on urologists’ SM was done by Koo et al [83]. Comparing the cohort in practice versus the cohort at the completion of residency, there were no significant differences in how many urologists had public Facebook accounts (70% vs 71%) or whose accounts had concerning content (43% vs 40%). Examples of concerning content included images and references to intoxication, explicit profanity, and offensive comments about patients. The presence of unprofessional content at the completion of residency strongly predicted having unprofessional content later in practice. A similar comparison was made among surgical residents and practicing surgeons [86,87]. In a study among surgical residents, 14.1% had potentially unprofessional content, and 12.2% had clearly unprofessional content. Binge drinking, sexually suggestive photos, and HIPAA violations were the most commonly found variables in the clearly unprofessional group [86]. Among attending surgeons, 10.3% had potentially unprofessional content, and 5.1% had clearly unprofessional content. Inappropriate language and sexually suggestive material were the most commonly found variables in the clearly unprofessional group [87]. Loo et al’s [99] study among faculty and residents in Singapore suggested that doctors within the same residency do not necessarily have a uniform set of professional priorities regarding professionalism on SM. Data from Kesselheim et al’s [80] study on pediatric residents clearly demonstrates “cognitive dissonance” in residents’ approach to lapses in professionalism while using SM. More than half of the responding residents rated posting of online comments about the workplace as “completely inappropriate,” yet a similar proportion estimate that residents engage in this behavior at least monthly [80]. Among nurses in China, 7.6% reported that they had “sometimes” posted identifiable patient information on SM. When asked about colleagues’ online professionalism, half (50.3%) of the participants indicated that they had “sometimes” witnessed their colleagues’ inappropriate SM posts and 49.5% reported “never” [107].

Among pharmacists, examples of perceived unprofessional behaviors included revealing details of personal life and activities; open complaints about the pharmacy sector, coworkers, physicians, and patients; inappropriate description of pharmacists’ roles and activities; and breaches of patient confidentiality [34].

#### Legal Issues and Disciplinary Consequences

Unprofessional behavior on SM of HCPs can have legal consequences, potentially affecting credibility and licensure. Several studies have emphasized this issue or reflected on disciplinary legal consequences if SM are used inappropriately [61,64,78,90,111,113,115,116].

Fuoco and Leveridge [78] raised the controversy of whether medical regulatory bodies should monitor the SM activities of HCPs. In all, 94.6% of respondents agreed that physicians need to exercise caution in personal SM posting, although 57% felt that medical regulatory bodies should “stay out of [their] personal SM activities,” especially those in practice for less than 10 years. Most urologists agreed that care should be taken in posting on SM sites, as unprofessional posts can put one at risk of discipline, so medicolegal guidance would be beneficial in this aspect as well. Duke et al [115] emphasized that use of SM platforms, while potentially beneficial, can have professional and legal implications if not used appropriately in both personal and academic use. Faculty and students need to be aware that this could negatively impact their professional image and the nursing profession [115].

In Great Britain since 2013, all General Dental Council (GDC) registrants’ online activities have been regulated by the GDC’s SM guidelines. Failure to comply with these guidelines results in a Fitness to Practice (FtP) complaint being investigated. Documentary analysis of FtP cases from September 2013 to June 2016 revealed that 6 complaints in relation to SM were investigated. A total of 2.4% of FtP cases published on the GDC website during that period were related to breaches of the SM guidelines. All of the cases investigated were proven and upheld. Most of those named in the complaints were dental nurses, and the most common type of complaint was inappropriate Facebook comments [90]. Staud and Kearny’s [113] study identified how online SM behaviors influence the licensure and enforcement practices of dental professionals. Dental boards are aware of potential online unprofessional behaviors and have implemented various consequences. Dental boards should consider developing policies to address potential online unprofessional behavior to protect the public that they serve [113]. In a recent study among Greek dental students, 75.3% of responders admitted not being aware whether the behavior of dentists on SM could result in legal sanctions [64].

Garg et al [116] conducted a survey of individual and institutional risks associated with the use of SM among residents and faculty in EM. EM residents and faculty members cause and encounter high-risk-to-professionalism events frequently while using SM; these events present significant risks to the individuals responsible and their associated institution. Some of the observations and occurrences documented in that study fall within the scope of HIPAA and put individuals and institutions at legal risk. The authors emphasize that, in addition to federal ramifications for medical institutions in regard to unprofessional conduct on SM by employees, the individuals responsible for the high-risk-to-professionalism events face state licensing consequences.

### Evaluation of Existing Approaches’ Effects on Promoting E-Professionalism

A total of 10 studies have tried to assess effectiveness of educational sessions or workshops incorporated in students’ or residents’ curriculum [46,48,52,54,55,60,63,81,89,103], and 1 study assessed the effects of formal SM instruction and policy on residents’ ability to navigate case-based scenarios about online behavior in the context of professional medicine [88]. In 4 studies that included medical students as participants [46,48,60,63], educational interventions were positively accepted by students and showed a positive impact on the way they view themselves and their use of SM.

Flickinger et al [46] stated that medical educators have an opportunity not only to provide valuable guidance to students in using SM wisely but also to promote the development of professional identities by implementing SM interventions into the medical curricula [46]. In a cohort study by Gomes et al [48], 94% of medical students reported some increase in awareness, and 64% made changes to their SM behavior due to the session, reflecting the longer-term impact. Walton et al [60] preformed an exploratory pre-post study to examine the internet presence of a Canadian medical school graduating class by scanning students’ public profiles on Facebook. They incorporated this information into an educational activity (3-hour long session) addressing professionalism and SM, and evaluated the impact of this activity on students’ SM behavior. Repeated searches for all class members 1 month following the educational intervention revealed that many students had changed their privacy settings to further restrict public access to information on their Facebook accounts. Fewer overall students could be found by any search strategy, and in particular, there was a significant decrease in the proportion that could be found using only a simple name search. Significantly, fewer students displayed personal information or friends lists. Finally, there was a significant reduction in the number of students who openly displayed large numbers of personal photographs [60].

A similar positive effect of educational intervention was described among nursing students by Marnocha et al [52]. The study assessed effects of a peer-facilitated SM education session on changes in attitudes and knowledge among recently admitted prelicensure nursing students. Participants described plans to use a more reflective, cautious, and accountable use of SM after the intervention. Uncertain or unprofessional attitudes and knowledge showed significant improvements after the intervention [52].

One study did not find a positive correlation between educational interventions and the impact on e-professionalism among students [55]. Medical students received two 45-minute educational sessions on digital professionalism. Findings of this study suggest that isolated sessions on professionalism are not sufficient to sustain perceptions and behaviors of professionalism. Their results reflect an erosion of professionalism related to information security that occurred despite medical school and hospital-based teaching sessions to promote digital professionalism. According to Mostaghimi et al’s [55] study, true alteration of trainee behavior will require a cultural shift that includes continual education; better role models; and frequent reminders for faculty, house staff, students, and staff.

A study conducted among pharmacy students showed that they are active users of peer-mediated SM learning groups. Pharmacy students have reservations regarding online professionalism and doubt the place of SM in education that includes the teacher [54].

In 3 studies that assessed the effectiveness of educational sessions on residents’ perception of e-professionalism [81,89,103], the positive impact was also determined. In Khandelwal et al’s [81] study, a postworkshop survey revealed that the postgraduate trainees perceived significant improvement in their understanding of e-professionalism. Compared with the preworkshop phase, residents were more comfortable defining professionalism, recognizing attributes of professionalism, describing the social contract, understanding the role of the code of conduct, and applying principles of professionalism to challenging scenarios [81]. Similar findings were presented in Mohiuddin et al’s [89] study where reflective practice-based sessions regarding the impact of SM on professionalism in surgery were well favored by the residents. Participants reported having an increased awareness to protect patient privacy and use SM more professionally [89]. Robertson et al [103] described a SM training program aimed to provide medical residents with academic and practical knowledge regarding the effective use of SM. Participants’ knowledge of SM policies increased as a result of the SM training. They have also increased the ability to identify potentially inappropriate media interactions and to identify appropriate responses to such interactions, and they gained an understanding of how their actions on SM affect others [103].

One study aimed to determine the effects of formal SM instruction and policy on residents’ ability to navigate case-based scenarios about online behavior in the context of professional medicine [88]. Prior SM instruction or familiarity with an SM policy were associated with improved performance on case-based questions regarding online professionalism.

### Barriers to Using Social Media for Health Care Professionals

Analyzing our review sample, we have recognized that some papers highlighted important aspects of barriers that influence HCPs use of SM in the context of e-professionalism. These barriers are lack of time or time constraint, lack of knowledge or technical skills, lack of previous education or supportive institutional SM policies, ignorance to existing SM policies, and problem developing and sustaining mutual trust on SM. HCPs perceived them less as risks and more as something that keeps them away from using SM, either at all, more often, or with more quality. Lack of free time or time constraint was often recognized as a barrier [39,69,74,79,91,93,96], as well as lack of knowledge or technical skills for use of SM [33,39,76,79,93,118]. The majority of these studies that recognize the lack of time or lack of knowledge as barriers have respondents on the level of practicing HCPs. By age distribution, representatives of “millennials” or “generation Z” were not included as study participants. As shown in Adilaman et al’s [74] study, this demonstrates a significant gap in SM use between younger users and mid- to late-career users. This study also found that midcareer physicians (aged 45-54 years) had statistically significantly more hesitations around joining medically geared SM sites for professional purposes, compared with those aged 25-34 years [74]. In a qualitative study among physicians by Campbell et al [76], participants expressed many levels of uncertainty about their preparedness, their impact, the potential for repercussions, and the future of physicians’ presence on SM. Participants described feeling unprepared when they started using SM. Many participants described concerns such as lacking knowledge about how to use certain SM platforms versus others. Several participants felt that they were “digital immigrants” [76].

A lack of previous education about SM was emphasized in several studies [33,98,102].

A lack of SM policies was also recognized as a barrier, either as a lack of models/guidelines in how to conduct themselves online in their role as physicians, which is manifested as fear of saying the wrong thing online [76] or related mainly to being unclear about whether they are supported by their employer and professional bodies [93,97]. Contrary to that finding, even if institutions have SM policies or guidelines, HCPs acknowledged reluctant behavior regarding existing SM policies [78,85,114] or ignorance to their existence [61,64,65,100]. A lack of awareness of existing institutional SM policies was also observed for physiotherapists; 41.6% were not aware whether there was one or not [85], and half of the medical students and faculty were unaware of existing institutional SM guidelines [114].

Panahi et al [36] recognized the problem of developing and sustaining mutual trust as one of the main barriers to knowledge sharing on SM platforms [36]. Physicians trust their peers on SM in a slightly different way than in face-to-face communication. The study found that the majority of participants established trust on SM mainly through previous personal interaction, authenticity and relevancy of voice, professional standing, consistency of communication, peer recommendation, and nonanonymous and moderated sites.

## Discussion

### Principal Findings

A scoping review method was used to capture the latest current evidence on e-professionalism of HCPs. The 88 studies included in this scoping review cover a broad spectrum of the benefits and dangers of SM on e-professionalism for HCPs, alongside barriers perceived as threats for the limitation of SM use in the context of e-professionalism and effects of existing approaches on promoting e-professionalism. This review includes multi-perspective views from various health care professions (medical, dental, nursing, pharmacy, and physiotherapy) and from various generations of HCPs (students, residents, practicing HCPs, faculty members, and PDs/deans). Overall, the quality of the studies was satisfactory. All studies were exploratory in nature, and the findings were descriptive. Medical health professionals were involved in about three-quarters of the studies. The majority of the studies were unspecific, studying use of any type of SM or SNSs. Only Facebook or “all SM/SNSs with specific reference to Facebook” was analyzed in more than one-third of the studies. Twitter [38,44,80,91,110], Instagram [101], and YouTube [37] were specifically targeted SM/SNSs in 7 studies.

#### Benefits of Social Media on E-Professionalism of Health Care Professionals

Benefits of SM on e-professionalism of HCPs can be seen as improvements of established networks or possibilities for collaboration through SM sites [33, 34, 36, 38, 39, 44-46, 53, 69, 72, 76, 78, 79, 92, 97, 106-108, 117]. Besides providing the opportunity for connecting with others and sharing experiences [38,39,46,53,69,72,78,106-108], SM have enabled the creation of communities for support. The benefits of SM on e-professionalism of HCPs, identified in this scoping review as professional networking and collaboration, have been documented in previous research for physicians [121-123], nursing profession [124,125], or other HCPs [126-129].

The benefits of peer advice, learning from peers, provision of emotional support, and identifying approaches through which physicians establish interpersonal trust on SM [36,45,92] are novel insights into the domain of e-professionalism of HCPs on SM.

Professional networking and collaborations on SM enable the development and building of professional identities for health care professions [34,38,39,45,67,73,104]. Professional identity formation among medical students now entails consideration by students about whether and how they can continue to use SM as physicians. Ruan et al’s [104] study tried to define the properties and development of the digital self and its interactions with the current professional identity development theory. SM introduces new features to professional identity in the digital world. The formation of digital identity, its development, and its reconciliation with other identities were features described, and educational institutions should give more importance to navigating professional identity development. According to Cruess et al [130], students may develop “identity dissonance” when components of their identity as physicians conflict with their identity as laypersons. Research regarding identity development in SM has been primarily confined to electronic professionalism through best practice guidelines. Evolving the possibilities of SM allows HCPs to reach a large audience and can act to increase their popularity among colleagues and patients [69]. SM also creates space for self-presentation and self-promotion that has already been embraced by some HCPs, enabling them to become microcelebrities [131].

Several studies have demonstrated students’ use of SM for acquiring knowledge, for gaining access to information from experts with whom they otherwise would not be able to connect, or for creating communities that can then be used as a means for supportive, professional, and social learning [44,45,58,66].

A number of studies have been conducted to investigate the ways in which health care students informally use SM for educational purposes [132]. The results identified efficient communication with educators, peer collaboration, and small group learning and sharing resources as key strengths [133]. SM has been proven to be used for educational purposes at medical schools, for example, to complement university courses [134,135]. SNSs can facilitate efficient communication, interactions, and connections among health professionals in education and training, with limitations identified as technical knowledge, professionalism, and risks of data protection [10]. Students’ use of SM for health education is overwhelmingly higher in the last few years, with almost the same proportion using SM often or always [69,136]. Our findings are consistent with previous research.

#### Dangers of Social Media on E-Professionalism of Health Care Professionals

According to some studies in this review, loosening accountability can be seen as a danger on e-professionalism from two points of view, eroding public trust by providing poor quality of information on SM [39,106,117] and damage to professional image [43, 45, 51, 56, 57, 59, 64, 66, 68, 70, 73, 102, 106, 112].

Potential damage to professional image has been depicted by students as a concern about repercussions of their posts, on career development, or on future employment since employers are checking SM profiles of candidates [43, 45, 51, 56, 57, 59, 66, 68, 70]. In addition, it has been reported that there is more awareness of online responsibilities as students progress through their program because employers can, and at times do, use SM profiles to make hiring decisions [56,137]. Three reviewed studies investigated PDs’ (medical and dental) attitudes about use of SM for admission criteria [109,110,112]. Students should be concerned about the level of professionalism presented on their profiles. Information available on SM has been already used regarding admissions to medical or nursing programs, selection for residence, or employment for over 8 years [137,138]. In 2016, the Mayo Clinic announced that it will take scholarly SM activity into account when considering academic promotion [139]. With time, it is reasonable to expect that more programs, schools, or any kind of potential employers of HCPs will use this “screening SM profiles” approach more often in the admissions process.

Compromising confidentiality concerns were described in numerous studies in this review [34, 43, 47, 57, 91, 94, 97, 107, 111, 119], especially about breaches of patient privacy or possible risks of violating HIPAA online. As previous research shows, the public availability of information on patients and physicians represents a threat to privacy [140-142], with the potential for a negative impact on patient-physician relationships [143,144]. Students and residents have a “cognitive dissonance” approach to lapses in their professionalism while using SM. It is a disconnect between what they thought they *would* do versus what they thought they *should* [47,80]. This inconsistency between attitudes and actions has been observed also elsewhere [145,146].

Traditional boundaries are blurred on many levels by online interactions. Blurred boundaries between professional and personal spheres of SM use [34, 39, 47, 51, 69, 78, 79, 91, 96, 97, 106], with concerns about exposure of one’s private life, presenting details of personal life, or separating private and professional profiles, have been presented in numerous studies in this review. The recommendation that health professionals maintain a separate account with a different name, a “dual citizen approach,” that maintains online professional and private identities by creating separate online profiles was introduced in 2011 by Mostaghimi and Crotty [147]. Surprisingly this issue is still prevalent. Several studies in this review investigated the purpose of SM use, whether participants mixed professional and personal information and activities on SM sites (blended profiles) or adopted a separation strategy where professional information and activities were clearly separated from personal ones (dual citizenship) [34,78,79,91,96]. Recent research shows that, for some HCPs, the risk of using SM is still a concern for the exposure of one’s private life [10,39,148].

Boundaries are blurred between patients and HCPs, and between students and faculty [40, 43, 47, 59, 64, 65, 68, 70, 73, 75, 78, 82, 85, 98, 100, 105, 107, 114, 117].

Although online interaction with a patient is generally not acceptable nor endorsed, a wide range of opinions have been observed concerning this issue, ranging from one-third for medical students in Brazil [59] that find this unacceptable to 92% for senior medical students in New Zealand [70]. This disproportion in range could be explained by cultural and age differences. Some studies have demonstrated generation gap differences in friending patients, with younger age being associated with more openness to be friends with a patient [82,117]. Both students and faculty are worried that connecting via SM would blur the boundaries of the teacher-student relationship, also recognized in other studies [148,149].

Chester et al’s [70] study in this review addresses a deficit in data and knowledge regarding patient-targeted Googling. This study provides a comprehensive understanding of patient-targeted Googling in concert with SNS use among senior New Zealand medical students. Results of this study show that 16.7% of respondents had conducted patient-targeted Googling. There is some evidence of an association between SNS use and likelihood of patient-targeted Googling, with high SNS users more likely to conduct patient-targeted Googling, but as the authors acknowledge, their observations were made on a small number of observations. Previous research in the United States showed that 2.3% of medical students had visited a patient’s profile on an online social network [150].

Various concerns about potential professionalism implications [151-153] exist, particularly related to breaches of patient confidentiality, professional boundaries, and depiction of unprofessional behaviors. Chretien and Tuck’s [14] review of online professionalism studies found that themes involving patient identifying images, inappropriate communications, and discriminatory language were consistently regarded as most inappropriate, whereas derogatory speech, images of alcohol, and partial nudity were considered only moderate to least inappropriate. Numerous studies in this review have tried to assess the extent of unprofessional behavior, posted by HCPs themselves or seen to be posted by their peers. Surveys that captured students’ self-report of posted unprofessional behavior (eg, evidence of being intoxicated, illegal drug use, posting patient information, sharing clinical images inappropriately, and depictions of an illegal act) reported witnessing the investigated examples with varying frequencies [32,42,43,55,59,62,64,73,114,118].

Age difference in the term “older and wiser,” meaning more cautious about posting unprofessional behavior online, was proven in studies comparing students’ and faculties’ online behavior [114,118]. A similar comparison was made among surgical residents and practicing surgeons with a decreasing percentage of unprofessional content among attending surgeons [86,87]. An interesting paradoxical observation from Kitsis et al’s [118] study is that, although students seemed more concerned than faculty about their professional images, their online behavior did not reflect this concern. Medical students reported that they considered their online presence to be unprofessional four times more often than faculty. In view of these findings, one might expect medical students to monitor their online presence regularly. Surprisingly, they rarely reported self-monitoring and at a rate similar to the faculty. This study shows that medical students’ posting of unprofessional material does not decrease during medical school and that medical students self-post and notice peers’ unprofessional posts more often than faculty do [118].

Other studies have determined important differences exist in perceptions of inappropriate SM behavior among various stakeholders. Medical students often regard themes of speech, alcohol, and dress as components of online ‘‘social identity’’ rather than potential unprofessional behavior [154,155]. In contrast, patients, supervisors, and regulatory groups demonstrate more conservative views. An online survey using mock Facebook profiles found that, compared to university students, faculty and members of the public rated images significantly less appropriate [156]. Survey results showed that among students there is little consensus on what constitutes unprofessional behavior beyond the US HIPAA violations, and students have felt that posting inappropriate material on personal SM sites was “unavoidable” [156].

It seems that consensus about what constitutes unprofessional behavior, even evoked as a question since Chretien et al’s [155] study in 2010, has still not been reached. There are numerous studies with examples of definitions of unprofessional behavior on SM [42,110,157,158]. Although there is no uniform consensus on what constitutes unprofessional behavior, studies most frequently associated it with online content pertaining to alcohol intoxication; substance or illegal drug use, nudity, and sexuality; demeaning content about patients, peers, educators, clinical sites, or the profession as a whole; discriminatory content; profanity; and aggressive/bullying content toward coworkers. Karveleas et al’s [64] study among dental students showed that students’ perceptions of and attitudes toward e-professionalism is complicated and contradictory. In their study, posting holiday pictures or wearing swimwear was categorized as unprofessional. Are these depictions of behaviors and situations unprofessional? What constitutes “potentially unprofessional behavior” has made quite a stir recently in medical scientific circles and the medical population in general.

In December 2019, a paper by Hardouin et al [159] was published investigating open, publicly available Facebook profiles of young vascular surgeons for unprofessional posts (text, images, or video content). The paper used a coding matrix, previously developed and used in other studies, for content analysis [83,84,87]. There were two distinct categories depicting e-professionalism of found content: “clearly unprofessional” and “potentially unprofessional.” Three male researchers created new anonymous Facebook profiles and screened through the available data. In the “potentially unprofessional” category, images of trainees in swimwear (bikinis) screened in the research were included. This sparked controversy primarily on Twitter but also on other SM sites and mainstream media about the objectivity and bias of the researchers, reviewers, and editors, creating a hashtag #medbikini [160]. A substantial number of HCPs participated in the outraged reaction to branding posting such images or videos in bikinis as a possible sign of unprofessional behavior. They posted this content with #medbikini and their disapproval of such a label and referred to the gender bias of the researchers, questioning possibly outdated norms of behavior for HCPs [161]. This ultimately led to the retraction of the paper and publication of a “Retraction notice” by the editors of the Journal of Vascular Surgery [162].

In a recently published paper by Pronk et al [163] that studied all levels of medical professionals (students, residents, and specialists), the authors found that all investigated groups perceived information or pictures to be unprofessional related to alcohol abuse, partying, and sexually suggestive posts, creating a dissonance between the #medbiniki movement’s perception of professionalism and collected data [163]. However, they argue that some of the participants’ opinions could have changed due to the debate initiated by the #medbikini movement, which occurred after their data collection.

Another recent study by Meira et al [164] investigating professionalism perception of orthodontist through exposure of laypeople, dental students, and dentists to images usually found on Instagram found that images related to social and family relationships were associated with lower scores regarding the perception of professional credibility for all groups [164]. They argued that their results indicate that personal images, possibly because they are not related to the professional context, contribute little toward the professional image of orthodontists on Instagram.

Unprofessional behavior on SM of HCPs can have legal consequences, potentially affecting credibility and licensure. Several studies have emphasized this issue or reflected on possible professional consequences if SM are used inappropriately [61,64,78,90,111,113,115,116].

Previous research has described associations of specific SM behaviors with the risk of investigation and subsequent disciplinary action by regulatory agencies by state medical boards and reported that online violations of professionalism by physicians were quite common and often led to disciplinary actions [120,165]. The consequences in the breach of privacy in the nursing profession can be severe and may lead to civil or criminal penalties [166]. Recent studies have also recognized that consequences of unprofessional online SM use can result in expulsion, lawsuits, job loss, and permanently damaged professional reputations [167]. This can also result in inaction or lack of use of SM for beneficial purposes, as the fear of legal issues can hinder use. This was recognized in a recent study by Al-Khalifa et al [168] where on a population of Saudi Arabian dentist only 41% of them were inclined to give online consultations. They argued the rest were possibly fearing potential legal ramifications. In an age of social distancing due to COVID-19, this could lead to patients not receiving information or care that they need and could have possibly gotten through online contact.

#### Recommendations for E-Professionalism Curriculum Changes

Ten years ago, many schools lacked policies specific to SM use [151], but nowadays schools have developed specific guidelines [169,170]. Guidelines are also available from numerous professional societies [171-178], and a recent review about available guidelines from nine medical international bodies has been published [179]. Previous work on health care education interventions and experiences has noted how learners may be motivated to reduce the hazards of SM, revise SM confidentiality settings, or even terminate SM involvement upon realizing that online postings may have an enduring presence [180,181].

Effectiveness of educational interventions about e-professionalism or impact of existing SM policies has been recognized in this review, since numerous studies explored educational interventions for promoting e-professionalism [46,48,52,54,55,60,63,81,89,103].

On an educational level for students, recommendations are to include a variety of e-professionalism topics into a curriculum to provide students with a clear picture of what constitutes professional violations on SM and assist them in distinguishing between personal and professional personas online [42,43,47,49,50,53,54,64,103,111,114]. Hsieh et al’s [63] study demonstrates the possibility of how SM can be used as a learning platform for professionalism, enabling students a virtual space in which to share positive examples that reflect the authentic experience in a clinical environment. Our previous findings demonstrate that the perception of unprofessional behavior varies among HCPs, mostly associated with age of the participants [86,109,114,118]. Similar findings were confirmed also for health science students who struggle with the concepts associated with professionalism [182]. Teaching professionalism in general offers challenges for educators, and these challenges are amplified when the topic moves into cyberspace, where students are digital natives and faculty are generally digital immigrants [136]. Several studies in this review have recognized the need to include students in the development of guidelines [47] or to assist in education with somebody of their age group, providing personal experiences and more of a “nonauthoritative” approach [49].

O’Sullivan et al [32] have also recognized the importance of schools using an evidence-based approach to policy creation and to involve students in the process of the creation of these policies. A recent study by Wissinger and Stiegler [183] also highlights the importance of formal integration of e-professionalism into the health care curricula to prepare students for situations they will face once employed. By placing the responsibility of learning e-professionalism inside the walls of academia, students are prepared to take control of their online identity and craft a persona that represents their professional image [167]. As Chretien and Kind [183] described it, a victory for online professionalism would be providing trainees with tools and guidance needed to ascend on the SM hierarchy pyramid of needs, from public trust to discovery.

Similar recommendations were described in this review for residents, with important issues that must be addressed during curriculum development: integrate trainees as educators, encourage peer-to-peer regulation, and provide opportunity for reflection. Effective educational interventions for teaching online professionalism must include the skills necessary for residents not only to recognize inappropriate behavior on SM but also to learn how to address it themselves [80,103]. There is a qualitative distinction between disseminating guidelines and formally integrating SM instruction into medical curricula, which should become imperative for HCP education, undergraduate or graduate level, or continuing medical education [42]. Similar conclusions were made in a systematic review of SM in residency [184]. Economides et al’s [184] review depicts evolving perceptions and a paradigm shift, where a growing body of literature is now focusing on promoting responsible SM use, examining how SM training can enhance professional growth and academic scholarship. As the tone of the dialogue shifts from trepidation to interest or even to enthusiasm, it is clear that there is a need for formalized standards and education on SM use established within the trainee’s curriculum.

#### Barriers to Social Media Use for Health Care Professionals

Analyzing our review sample, we have recognized that some papers highlighted important aspects of barriers that influence HCPs use of SM in context of e-professionalism. A lack of free time or time constraint was often recognized as a barrier [39,69,74,79,91,93,96], as well as a lack of knowledge or technical skills for use of SM [33,39,76,79,93,118]. Studies in this review with “lack of time” or “lack of knowledge” barriers had respondents that were practicing HCPs and older HCPs; representatives of “millennials” or “generation Z” were not included as study participants. This demonstrates a significant gap in SM use between younger users and mid- to late-career users [74,82]. This is consistent with previous research demonstrating that in addition to the practical barriers to adoption of SM in the professional realm, a generation gap exists, with millennials using SM for contact and information far more frequently than members of generation X and baby boomers [150]. Similar results can be found in Chan et al’s [10] systematic review where identified positive predictors of use of SNSs for professional purposes were younger age (20-39 years), fewer years of professional experience (0-10 years), and lower rank, such as residents.

Our results show that even though a lack of SM policies was recognized as a barrier, even if institutions have SM policies or guidelines, HCPs acknowledged reluctant behavior regarding existing SM policies [78,85,114] or ignorance to their existence [61,64,65,100]. This should be considered as a warning to increase awareness on this matter, as SM will continue to be increasingly ubiquitous and integrated in health care. As Parsi and Elster [185] note, “if we fail to engage this technology constructively, we will lose an important opportunity to expand the application of medical professionalism within contemporary society.” Since the SM world is changing so fast, adopting novel approaches to existing SM policies becomes essential. As Kerr et al [101] suggest, it is imperative for nursing education, professional regulatory bodies, and employers to develop more robust and dynamic policies and guidelines related to the appropriate use of SM within the profession, especially with the growing presence of web-based HCP microcelebrities [131].

### Comparison With Prior Work

Compared to other literature reviews published on related topics, this scoping review is the first to capture original research about e-professionalism in terms of methods, subjects, and themes since Chretien and Tuck [14], who conducted a synthetic review to characterize the original peer-reviewed research on online professionalism of medical students, residents, or physicians. The review included 32 studies and recognized general areas of online professionalism (use and privacy, assessment of unprofessional behavior, consensus-gathering of what constitutes unprofessional or inappropriate behaviors, and education and policies) with no clear separation between challenges or benefits and addressed only online professionalism of medical students, residents, or physicians. Other reviews presented a full spectrum of SM-related challenges and opportunities in the context of medical professionalism of diverse types of HCPs [15,16] or in the context of SM as an emerging tool in education [132,186], but these studies were conducted several years ago.

Similar conclusions were made in other research. Although there exist concerns about misuse of SM and violation of e-professionalism by HCPs, SM can also be used constructively as a tool for professional development; as a means of accessing information, marketing practices and services, job opportunities; and as a means of sharing or adding opinions on issues of interest to HCPs and to other like-minded individuals online [44,187].

Ventola [121] recognized benefits, risks, and best practices for HCPs. He concluded that SM can provide considerable benefits in professional networking or collaboration, professional education, patient’s care, education, and health programs. All these benefits of SM were recognized in this scoping review as well. According to Ventola [121], there are some risks related to poor quality of information, damage to professional image, breaches of patient privacy, violation of the patient-HCP boundary, and licensing and legal issues, which was also recognized in this scoping review. Likewise, risks such as privacy and accuracy of information, compromising confidentiality, eroding public trust, and loosening accountability were presented in previous research [156,188,189].

### Gaps in the Literature and Potential Areas for Further Research

This review demonstrates dominance of Facebook in research done so far. With the rapid evolution of SM, future insights should be more oriented toward new and emerging SM sites. Instagram has gained an enormous following with new features like “Stories” and “Reels” within the SM itself, which are completely scientifically unexplored. Snapchat and TikTok have also gained a substantial following, especially among the student population. TikTok did not even exist in 2014; nowadays, TikTok has 689 million users worldwide [20]. They function on a completely different set of parameters, being that the content is time limited. New research should consider how to approach the youngest generation of HCPs who are using these SM sites and how to design a novel study methodology to gain insight, due to the time limitation of the content.

Gaining popularity on SM is not only reserved for adolescents and young adults. Creation of influencers who are HCPs can affect perception of the professional image, either positively or negatively. This has been rarely analyzed so far.

Geographical locations may affect the generalization of findings in research on SM use. Asian countries have regionally oriented SM and SNSs like WeChat not used in European countries or the United States. Cultural differences should also be considered.

The curriculum implementation of SM guidelines and educational efforts are also there to be evaluated. As the diversity of such actions is apparent, efficiency is key in getting the proper message to a generation of “millennials” with a short attention span.

The COVID-19 pandemic has caused much of the world’s population to isolate itself and many of us to shift our lives to digital tech platforms, especially SM and SNSs, all experiencing strong growth. Previous research has shown that more people are relying on SM to find and share health information during times of crisis. Future research should investigate how the pandemic affected our e-professional behavior. We are experiencing an unprecedented time in health care and education due to the COVID-19 pandemic, so the use of SM in patient/HCP communication and student education should also be explored in more detail.

SM can also be used in marketing and self-promotion [190]. Dental medicine is much more open to such actions, with medical professionals showing a lack of interest and being more worried about legal ramifications. Research into the reasons of such divergence and insight into the negative attitudes is essential for creating a platform for implementing SM in a positive and professional manner.

Findings of this research confirm the dominance of medical students or physicians as a study population of HCPs in the context of e-professionalism. Future research could be done to further investigate other types of HCPs with an emphasis on the specifics of each profession regarding their SM potential and use. Comparison among different types of HCPs would add novel insights to the field of e-professionalism.

### Limitations of the Review

We acknowledge that scoping reviews have several limitations, but a scoping review allowed us to gain a wide-ranging understanding of the impact of SM on e-professionalism of HCPs. Research into SM is rapidly growing, and this scoping review is a snapshot of the latest current evidence on e-professionalism of HCPs.

There might be a selection bias (failure to search in additional potentially relevant databases to which the university does not have access) and a publication bias (we only searched in 3 databases, we did not extensively search for gray literature, and our search was limited to the English language). All studies were exploratory in nature, and the findings were descriptive. The questionnaires adopted in the surveys were mostly developed by the researchers, where validation mostly was not done. Research on SM is growing so fast that evidence may have been published in electronic media or platforms not indexed through the academic databases. Thus, findings in this review are limited to research published in traditional peer-reviewed journals only.

### Conclusions

A scoping review was conducted that included 88 studies, offering current evidence on e-professionalism of HCPs. Almost all studies were found to be of adequate quality. Findings in reviewed studies indicate the existence of both benefits of SM on e-professionalism such as professional networking and collaboration, training, and education, and, on the other hand, the dangers of SM, such as loosening of accountability, compromising doctor-patient confidentiality, blurred professional boundaries, depiction of unprofessional behavior on SM, and legal consequences.

Even though there are some barriers recognized, this review has highlighted existing recommendations for including e-professionalism in educational curricula of HCPs. Based on all evidence provided, this review provided new insights and guides for future research on this area. There is a clear need for robust research to investigate new emerging SM platforms, the efficiency of guidelines and educational interventions, and the specifics of each profession regarding their SM potential and use.

## Appendix

Multimedia Appendix 1 Search strategy.

Multimedia Appendix 2 Summary of the reviewed studies (n=88).

Multimedia Appendix 3 List of the excluded studies and the reasons for exclusion.

Multimedia Appendix 4 Quality of the reviewed studies.

## Abbreviations

EM: emergency medicine
FtP: Fitness to Practice
GDC: General Dental Council
HCP: health care professional
HIPAA: Health Insurance Portability and Accountability Act
IRR: interrater reliability
MeSH: Medical Subject Headings
PD: program director
PRISMA-ScR: Preferred Reporting Items for Systematic Reviews and Meta-Analyses Extension for Scoping Reviews
SM: social media
SNS: social networking site
