# Supplementary material for: Dangers and Benefits of Social Media on E-Professionalism of Health Care Professionals: Scoping Review
Source: J Med Internet Res. 2021 Nov 17;23(11):e25770. doi: 10.2196/25770 (PMC8663533; doi:10.2196/25770)
Supplement: Multimedia Appendix 1 [file jmir_v23i11e25770_app1.pdf]

Search strategies:

Searches conducted in January 2021

Search strategy used in CINAHL (EBSCO)

The Cumulative Index to Nursing and Allied Health Literature

Interface-EBSCOhost Research Databases

Search Screen-Advanced Search Database – CINAHL Plus with Full Text

Limiters/expanders: English; time: 01.11.2014.-31.12.2020.

#	Query	Results
S1	(MH "professionalism")	2,597
S2	TI (professionalism)	678
S3	AB (professionalism)	1,567
S4	S1 OR S2 OR S3	3,710
S5	(MH "Social Media")	11,590
S6	(MH "Internet")	13,535
S7	TI (social media OR social network* OR Internet)	8,687
S8	AB (social media OR social network* OR Internet)	22,066
S9	S5 OR S6 OR S7 OR S8	38,796
S10	TI (Facebook OR Twitter* OR Tweet* OR Instagram OR TikTok)	2,835
S11	AB (Facebook OR Twitter* OR Tweet* OR Instagram OR TikTok)	4,574
S12	S10 OR S11	5,628
S13	S4 AND (S9 OR S12)	330

Search strategy used in PubMed and Scopus

Limiters/expanders: English; time: 01.11.2014-31.12.2020.

**DATABASE**	**SEARCH ENGINE**	**OUTPUT**
PubMed	professionalism[Title/Abstract] AND social media[Title/Abstract]<br>*Filters applied: English, from 2014/11/1 - 2020/12/31.*	191
PubMed	professionalism[Title/Abstract] AND social networks[Title/Abstract]<br>*Filters applied: English, from 2014/11/1 - 2020/12/31.*	7
PubMed	professionalism[Title/Abstract] AND Internet[Title/Abstract]<br>*Filters applied: English, from 2014/11/1 - 2020/12/31.*	65
PubMed	professionalism[Title/Abstract] AND Facebook[Title/Abstract]<br>*Filters applied: English, from 2014/11/1 - 2020/12/31.*	41
PubMed	professionalism[Title/Abstract] AND Twitter[Title/Abstract]<br>*Filters applied: English, from 2014/11/1 - 2020/12/31.*	37
PubMed	professionalism[Title/Abstract] AND Instagram[Title/Abstract]<br>*Filters applied: English, from 2014/11/1 - 2020/12/31.*	8
PubMed	professionalism[Title/Abstract] AND TikTok[Title/Abstract]<br>*Filters applied: English, from 2014/11/1 - 2020/12/31.*	0
PubMed	"professionalism"[MeSH Terms] AND "social media"[MeSH Terms]<br>*Filters applied: English, from 2014/11/1 - 2020/12/31.*	84
PubMed	"professionalism"[MeSH Terms] AND "Internet“[MeSH Terms]<br>*Filters applied: English, from 2014/11/1 - 2020/12/31.*	102
PubMed	professionalism[Other Term] AND (social media[Other Term] OR social networks[Other Term] OR Internet[Other Term] OR Facebook[Other Term] OR Twitter[Other Term] OR Instagram[Other Term] OR TikTok[Other Term])<br>*Filters applied: English, from 2014/11/1 - 2020/12/31.*	65
Scopus	TITLE-ABS-KEY (professionalism) AND TITLE-ABS-KEY ("social media") AND (LIMIT-TO (SUBJAREA , "MEDI") OR LIMIT-TO (SUBJAREA , "NURS") OR LIMIT-TO (SUBJAREA , "HEAL") OR LIMIT-TO (SUBJAREA, *"PHAR"*)) AND (PUBYEAR > 2013 AND PUBYEAR < 2021) AND (LIMIT-TO (LANGUAGE , "English"))	328
Scopus	TITLE-ABS-KEY (*professionalism*) AND TITLE-ABS-KEY (*internet*) AND (LIMIT-TO (SUBJAREA , *"MEDI"*) OR LIMIT-TO (SUBJAREA , *"NURS"*) OR LIMIT-TO (SUBJAREA , *"HEAL"*) OR LIMIT-TO (SUBJAREA, *"PHAR"*)) AND (PUBYEAR > 2013 AND PUBYEAR < 2021) AND (LIMIT-TO (LANGUAGE , *"English"*))	161
Scopus	TITLE-ABS-KEY (*professionalism*) AND TITLE-ABS-KEY (*"social networks"*) AND (LIMIT-TO (SUBJAREA , *"MEDI"*) OR LIMIT-TO (SUBJAREA , *"NURS"*) OR LIMIT-TO (SUBJAREA , *"HEAL"*) OR LIMIT-TO (SUBJAREA, *"PHAR"*)) AND (PUBYEAR > 2013 AND PUBYEAR < 2021) AND (LIMIT-TO (LANGUAGE , *"English"*))	84
Scopus	TITLE-ABS-KEY (*professionalism*) AND TITLE-ABS-KEY (*Facebook*) AND (LIMIT-TO (SUBJAREA , *"MEDI"*) OR LIMIT-TO (SUBJAREA , *"NURS"*) OR LIMIT-TO (SUBJAREA , *"HEAL"*) OR LIMIT-TO (SUBJAREA, *"PHAR"*)) AND (PUBYEAR > 2013 AND PUBYEAR < 2021) AND (LIMIT-TO (LANGUAGE , *"English"*))	53
Scopus	TITLE-ABS-KEY (*professionalism*) AND TITLE-ABS-KEY (*Instagram*) AND (LIMIT-TO (SUBJAREA , *"MEDI"*) OR LIMIT-TO (SUBJAREA , *"NURS"*) OR LIMIT-TO (SUBJAREA , *"HEAL"*) OR	12

	LIMIT-TO (SUBJAREA, *"PHAR"*)) AND (PUBYEAR > 2013 AND PUBYEAR < 2021) AND (LIMIT-TO (LANGUAGE , *"English"*))	
Scopus	TITLE-ABS-KEY (*professionalism*) AND TITLE-ABS-KEY (*twitter*) AND (LIMIT-TO (SUBJAREA , *"MEDI"*) OR LIMIT-TO (SUBJAREA , *"NURS"*) OR LIMIT-TO (SUBJAREA , *"HEAL"*) OR LIMIT-TO (SUBJAREA, *"PHAR"*)) AND (PUBYEAR > 2013 AND PUBYEAR < 2021) AND (LIMIT-TO (LANGUAGE , *"English"*))	51
Scopus	TITLE-ABS-KEY (*professionalism*) AND TITLE-ABS-KEY (*TikTok*) AND (LIMIT-TO (SUBJAREA , *"MEDI"*) OR LIMIT-TO (SUBJAREA , *"NURS"*) OR LIMIT-TO (SUBJAREA , *"HEAL"*) OR LIMIT-TO (SUBJAREA, *"PHAR"*)) AND (PUBYEAR > 2013 AND PUBYEAR < 2021) AND (LIMIT-TO (LANGUAGE , *"English"*))	0
