# Supplementary material for: Dangers and Benefits of Social Media on E-Professionalism of Health Care Professionals: Scoping Review
Source: J Med Internet Res. 2021 Nov 17;23(11):e25770. doi: 10.2196/25770 (PMC8663533; doi:10.2196/25770)
Supplement: Multimedia Appendix 2 [file jmir_v23i11e25770_app2.pdf]

Study (Year)	Study Objective	Study design	Data Analysis Method	Study population (Sample/ Participants)	Country of origin	Type of Social Media (SM)	Key measures	Conclusions/ Recommendations	Ethics statement
**Undergraduate HCPs (n=35)**									
Chretien, Tuck, Simon, Singh, Kind. 2015 [1]	To explore how and why medical students use Twitter for professional development.	Digital ethnography (qualitative)	i. Content analysis of tweets<br><br>ii. Semi-structured interviews with „super users "<br><br>iii. Network analysis	i. Postings (tweets) of 31 medical students<br><br>ii. Semi-structured interviews with 10 medical students „super users"	USA	Twitter	Content of tweets, number of tweets, number of followers, number followed content analysis for URLs, retweets, and hashtags, characteristics of the network	Twitter can serve as a professional tool that supplements traditional education. Twitter provided value in two major domains: access and voice.	Yes, approved
Marnocha, Marnocha, Cleveland, Lambie, Limberg, Wnuk. 2017 [2]	To (1) address online professionalism knowledge and attitudes within a class of newly admitted nursing students and (2) examine the effects of a peer-facilitated educational intervention intended to improve awareness and understanding of such professionalism.	Longitudinal, observational study (quantitative)	Descriptive analysis	72 nursing students (RR 100%)	USA	All types of SM	Usage of SM sites, daily hours on SM, personal significance of SM, knowledge about university SM policy, assessment of risky online postings, SM professionalism principles, SM knowledge	Uncertain or incorrect attitudes and knowledge showed significant improvements after the intervention. Such interventions may enhance cyber-professionalism in future student cohorts and warrant further exploration.	Yes, approved
Nason, Byrne, Nason, O'Connell. 2018 [3]	To assess the level of online professionalism on Facebook profiles available for public viewing of students from a dental school	Mixed methods (qualitative and quantitative)	i. Content analysis of Facebook profiles<br><br>ii. Descriptive analysis	287 students in the dental school (RR 62%)	Ireland	Facebook	Presence of profile, privacy settings, personal information, identifying patient information, photo content, rating of professionalism	The use of Facebook is prevalent amongst dental students. Despite the use of standard security settings, a concerning level of unprofessional content was visible. The dental school needs to consider an educational module regarding the management of personal profiles in a professional setting.	Yes, approved
Flickinger, O'Hagan, Chisolm. 2015 [4]	To develop a curriculum to improve knowledge and skills regarding	Mixed methods (quantitative	i. Descriptive analysis	i. 72 medical students (RR 30%)	USA	All types of SM	i. Students' SM use, rationale for and frequency of use, and concerns.	Most medical students surveyed reported using SM and identified privacy and personal-professional	Yes, approved

	professionalism of SM use by medical students.	and qualitative).	ii. Qualitatively via participant feedback<br><br>iii. Quantitatively and qualitatively via personal growth scales, participant feedback, and analysis of blog themes.	ii. 91 medical students completed the post workshop evaluation (RR 76%)<br><br>iii. SM based curriculum was evaluated by a small-scale pilot of 11 students			ii. Workshop-format curriculum was designed and piloted for preclinical students to gain foundational knowledge of online professionalism<br><br>iii. A complementary longitudinal SM-based curriculum was designed and piloted for clinical students to promote both medical humanism and professionalism	boundaries as areas of concern. The workshop format and SM-based curricula were well received by students whose formative feedback will inform the refinement and further development of efforts to promote professionalism among medical students.	
George, Navarro, Stazyk, Clark, Green. 2014 [5]	To explore how students use Facebook, with a particular emphasis on how they think they and their peers should (and would) act in response to salient ethical dilemmas.	Mixed methods Cross-sectional survey (quantitative and qualitative)	i. Descriptive analysis<br><br>ii. Thematic analysis	2,109 medical students (RR 30%)	USA	Facebook	SM use patterns and preferences, ethical dilemmas in the use of SM, privacy, the patient–physician relationship (“friending” patients and seeking students' advice via SM) and students’ relationships with peers and colleagues	Confirms previous research demonstrating ubiquitous use of Facebook among students, while suggesting that students are aware of potential ethical pitfalls of Facebook misuse.	Yes, exempted
Henning, Hawken, MacDonald, McKimm, Brown, Moriarty, et al. 2017 [6]	To establish the most effective approach and type of educational intervention for health professional students, to enable them to maintain a professionally safe online presence.	Exploratory qualitative study (qualitative)	i. Content analysis of interviews and focus groups<br><br>ii. Thematic analysis of interviews and focus groups	57 nursing, medical and paramedical students (RR not stated)	Australia	Facebook	The use of SM, mainly with specific reference to Facebook (but relevant to the use of other SM sites), students’ preferences for educational approaches for training about the use of Facebook	Supports the need for an educational intervention to assist health professional students to navigate SM sites safely and in a manner appropriate to their future roles as health professionals.	Yes, approved
Nicolai, Schmidbauer, Gradel, Ferch, Antón, Hoppe et al. 2017 [7]	To (1) identify the role of study-related Facebook group use, (2) characterize medical students that use or avoid using Facebook groups (demographics, participation pattern, and motivation), and (3) analyze student posting behavior, covered topics, dynamics, and limitations in Facebook	Mixed methods explorative study (quantitative and qualitative)	i. Thematic and content analysis of posts<br><br>ii. Semi-structured interviews<br><br>iii. Focus groups among Facebook users<br><br>iv. Coding scheme for studying the	i. 950 medical students in preclinical year 1 (1,168 posts) and 966 medical students in preclinical year 2 (1,246 posts)<br><br>ii. 4 students<br><br>iii. 21 students	Germany	Facebook	Motivation to join in Facebook groups, characterization of medical students involved in Facebook groups, participation pattern, posting behavior, efficiency, identification of the type of Facebook group user.	Facebook groups are an essential part of the learning environment for most medical students. For the moment, the medical faculty has no active involvement in these groups and therefore no influence on accuracy of information, professionalism, and ethical issues. Nevertheless, faculty	Yes, exempted

	groups with regards to educational usage		frequency and distribution of posts (quantification of qualitative data)					could in the future benefit by extracting relevant information, identifying common problems, and understanding semester-related dynamics.	
Ferguson, Di Giacomo, Saliba, Green, Wyllie, Moorley et al. 2016 [8]	To explore first year nursing student experiences with social media in supporting student transition and engagement into higher education.	Exploratory qualitative study (qualitative)	Thematic content analysis of focus groups	10 nursing students (in 3 focus groups)	Australia	All types of SM	Experiences of transition and engagement of first year student nurses using SM at university with peers and academic staff	Study has demonstrated the importance of SM in supporting informal peer–peer learning and support, augmenting online and offline relationships, and building professional identity as a nurse.	Yes, approved
Ness, Sheehan, Snyder. 2014 [9]	To (1) characterize students' views and opinions of professionalism on popular social media sites and (2) compare responses about social media behavior among students in different groups.	Cross-sectional survey (qualitative)	Thematic analysis of online survey with open-ended questions	212 graduating student pharmacists (RR 41%)	USA	All types of SM	Reasons for not having a SM profile, definition of e-professionalism, accountability for illegal or unprofessional acts discovered through SM, opinions regarding employers researching and considering candidates' SM profiles when making hiring decisions, use of privacy settings, SM profile representation of students as individuals and professionals, SM's effect on public opinion of students as health care professionals, inappropriate information posted, profile edits before interviews or career fairs	Major overarching themes identified were separation of personal and professional lives, how accountability for actions should vary by severity, and the extent of representation of the students' character on SM. Identified themes provided important insights into the ways in which student pharmacists view SM and use this widely accessible means of personal communication.	Yes, approved
Bagley, Di Giacinto, Lawyer, Anderson. 2014 [10]	To (1) document how students enrolled in medical programs are using Facebook and (2) assess the students' general understanding of what constitutes Health Insurance Portability and Accountability Act (HIPAA) violations on Facebook.	Cross-sectional survey (quantitative)	Descriptive analysis	137 students of medical programs, including 10 incomplete surveys (RR 4,48%)	USA	Facebook	How often students access Facebook, update status information, or post photographs online; HIPAA violation scenarios	Frequency that a student updates a Facebook status appears to be associated with a risk of violating HIPAA online. Further research is needed to identify what additional characteristics put students at risk for violating HIPAA so that interventions may be	Yes, approved

								developed to prevent students from behaving unprofessionally or unlawfully through SM.	
Barnable, Cunning, Parcon. 2018 [11]	To assess nursing students’ Facebook activity (how often and how long) and their perceptions about accountability, confidentiality, and e-professionalism in relation to Facebook.	Cross-sectional survey (quantitative)	Descriptive analysis	97 nursing students (RR 46%)	Canada	Facebook	Accountability, e-professionalism and confidentiality related to students’ Facebook use	The use of Facebook is prevalent among nursing students. Despite the use of privacy settings and students receiving education about professionalism, unprofessional content is still visible. Students need to be aware of their professional responsibility when using Facebook. The school of nursing should consider revising and reinforcing the education students receive about unprofessional online behavior on Facebook.	Yes, approved
Rocha, de Castro. 2014 [12]	To (1) determine the frequency with which students from a Brazilian Medical School come across ten given examples of unprofessional online behavior by medical students or physicians, and (2) gather the opinions of participants regarding the appropriateness of these behaviors.	Cross-sectional survey (quantitative)	Descriptive analysis	336 medical students (RR 96%)	Brazil	All types of SM	Presence of profile, unprofessional online behavior, potential implications of posts in SM	Medical students are witnessing a high frequency of unprofessional online behavior by their peers and physicians. Most investigated behaviors were considered inappropriate, especially if carried out by physicians.	Yes, approved
Alkhateeb, Alameddine, Attarabeen, Latif, Osolin, Khanfar, Al-Rousaz. 2015 [13]	To (1) evaluate pharmacy students' adoption and behavior related SM in general, and to Facebook in particular; (2) explore faculty's adoption of Facebook from students' perspectives, as well as	Cross-sectional survey (quantitative)	Descriptive analysis	193 pharmacy students (RR 99%)	USA	Facebook	Pharmacy students adoption of SNS, types of Facebook usage by pharmacy students, students adoption of Facebook	Pharmacy students are mainly active on Facebook, yet they have selective online presence and interaction. From students' perspectives, an active user is generally open to "friending" the outside world. However, the majority is still	Yes, approved

	students' willingness, to "friend" their faculty.							reluctant to "friend" faculty members at their school of pharmacy. Most students choose to use Facebook for social purposes rather than for educational use.	
Mawdsley. 2015 [14]	To (1) measure students' perceptions of SM in education and (2) consider how SM may be incorporated as a complementary learning tool.	Mixed methods (quantitative and qualitative)	i. Descriptive analysis<br><br>ii. Thematic analysis	196 pharmacy undergraduate students (RR 31%)	UK	All types of SM	Privacy and confidentiality, access to technology, peer socialization, enforcement of SM, acceptance of SM, past experience with SM use	Pharmacy students are active users of peer-mediated SM learning groups. Students have reservations regarding online professionalism and doubt the place of SM in education that includes the teacher. Desire for favorable perceptions on professionalism, and lack of experience with SM as a teaching method, negatively impacts the genuine use of SM for educational purposes.	None stated
Mather, Cummings, Nichols. 2016 [15]	To explore first and final year undergraduate student use of SM to understand how it was utilized by them during their course	Cross-sectional survey (quantitative)	Descriptive analysis	126 nursing students<br>(RR not stated)	Australia	All types of SM	Primary source of information, frequency and patterns of usage of specific SM sites including Facebook, Twitter and LinkedIn	The majority of nursing students use SM for a range of purposes and in a range of locations, indicating a need to update nursing curricula to enable learning about appropriate use of these platforms. Classroom and simulation activities should be used to model appropriate use of SM. Teaching undergraduate students SM etiquette in a safe environment can ensure the next generation of health professionals develop and promote positive professional identity and professionalism regarding the use of digital technologies and SM.	None stated

Kenny, Johnson. 2016 [16]	To (1) examine dental student attitudes towards professional behavior on social media, (2) establish the extent and nature of social media use and exposure to potentially unprofessional behaviors.	Cross-sectional survey (quantitative)	Descriptive analysis	155 dental students (RR 90%)	Wales	All types of SM	Frequencies of SM use, most used privacy settings, usage of real name, professionalism behavior statements, photo content	This study highlights the need for SM training for all dental undergraduates, as SM use is widespread.	Yes, approved
Jawaid, Khan, Bhutto. 2014 [17]	To understand the frequency and ways in which Facebook is used by the medical students in Pakistan.	Mixed methods (quantitative and qualitative)	i. Descriptive content analysis of Facebook profiles<br><br>ii. In depth qualitative content analysis of Facebook profiles	i. 535 medical students<br><br>ii. 10 medical students	Pakistan	Facebook	Presence of profile, privacy settings, personal information, personal views	The educators must encourage the active discussions about Social platform and have training session of students about proper use of these sites so that the students can have better professional life in future.	Yes, approved
Gomes Butera, Chretien, Kind. 2017 [18]	To (1) raise awareness of inappropriate SM behavior and possible ramifications but also (2) draw attention to the increasing numbers of medical professionals utilizing SM in positive ways.	Cohort mixed methods study (qualitative and quantitative)	i. Descriptive analysis<br><br>ii. Qualitative thematic data analysis	i. 313 medical students (RR 62%)<br>- 1st cohort 62 students (RR 36%)<br>- 2nd cohort 125 students (RR74%)<br>- 3rd cohort 126 students (RR 76%)<br><br>ii. Follow-up (3rd cohort) 76 students (RR 44%)	USA	All types of SM	On-line presence, professional behavior on SM, changes of SM identities, guidelines on social media, impact of SM educational session	Discussion of online professionalism should not be limited to negative inappropriate behaviors and subsequent ramifications but must include examples of positive professional use. A panel of mentors sharing their experience in leveraging SM for professional purposes provides students with needed role models and should be a part of the session.	Yes, exempted
Barlow, Morrison, Stephens, Jenkins, Bailey, Pilcher. 2015 [19]	To (1) describe the SM usage patterns of medical students and (2) identify factors associated with their posting of unprofessional content on SM	Cross-sectional survey (quantitative)	Descriptive analysis	880 medical students (RR 86%)	Australia	All types of SM	Use of SM sites, prevalence of unprofessional online behavior, guidelines on SM	Posting of unprofessional content was highly prevalent despite understanding that this might be considered inappropriate, and despite awareness of professionalism guidelines. Medical	Yes, approved

								educators should consider approaches to this problem that involve more than simply providing guidelines or policies, and students should be regularly prompted to evaluate and moderate their own online behavior.	
Walton, White, Ross. 2015 [20]	To (1) examine the presence of a graduating class of Canadian medical students on a popular SM site (Facebook) and (2) develop and then evaluate the impact of an educational intervention focused on professionalism and SM on student behavior.	Exploratory cohort (pre and post) study (quantitative)	Descriptive analysis	121 medical students (RR not stated)	Canada	Facebook	Existence of SM profile, availability of personal information, visible comments and posts, publicly visible photographs, nature of postings, privacy settings, unprofessional content	Even within a cohort of senior medical students who are very familiar with technology, many individuals are not as vigilant about their privacy settings on SNS. Reflecting this information back to them as part of an educational intervention proved to be engaging, popular, and ultimately a highly effective strategy for both opening a dialogue and changing student behavior.	Yes, approved
Mostaghimi, Olszewski, Bell, Roberts, Crotty. 2017 [21]	To explore professional behaviors specific to appropriate use of technology by looking at changes in third-year medical students' attitudes and behaviors at the beginning and conclusion of their clinical clerkships	Cross-sectional survey (quantitative)	Descriptive analysis	51 medical students (RR pre- and post-clerkship surveys were 96% and 86%)	USA	All types of SM	Privacy, information security, communications, and SM, boundaries, and online tone, digital identity, perceptions of technology usage in professional setting	Erosion of professionalism related to information security that occurred despite medical school and hospital-based teaching sessions to promote digital professionalism. True alteration of trainee behavior will require a cultural shift that includes continual education, better role models, and frequent reminders for faculty, house staff, students, and staff.	Yes, exempted
O'Sullivan et al. 2017 [22]	To (1) examine health science students' opinions on the use of	Cross-sectional	Descriptive analysis	1,343 health science students (RR 81,89%)	China, Mexico, Ireland,	All types of SM	Factors that would encourage students to use SM in their education.	A significant number of students across all health science disciplines self-	Yes, approved

	SM in health science education and (2) identify factors that may discourage its use	survey (quantitative)			Canada, United Kingdom, Hong Kong, Australia		The impact of SM training on students' confidence in using SM.<br>rates of sharing of different items among users and nonusers without explicit permission.<br>Frequency of SM use and inappropriate sharing by health science students	reported sharing clinical images inappropriately, and thus request the need for policies and training specific to SM use in health science education.	
Avcı, Çelikden, Eren, Aydenizöz 2015. [23]	To evaluate the use of SM and attitudes toward its use in medicine among medical students.	Cross-sectional survey (quantitative)	Descriptive analysis	681 medical students (RR 70,8%)	Turkey	All types of SM	The frequency of SM use and attitudes toward the use of SM in medicine.	93.4% of medical students used SM and 89.3% used SM for professional purposes. Factor analysis showed that attitudes toward SM are based on five factors: professional usefulness, popularity, ethics, barriers, and innovativeness. A structural equation model revealed the highest positive correlation between usefulness and innovativeness; ethics had a low but positive correlation with other factors.	Yes, approved
Gupta, Singh, Dhaliwal. 2015 [24]	To assess medical students' social networking presence and the degree of visible activity on their profiles, with particular reference to unprofessional content	Mixed methods cross-sectional survey (quantitative and qualitative)	i. Descriptive analysis<br><br>ii. Content analysis	477 medical students (RR 78.1%)	India	Facebook	Personal information, prevalence of identifiable profiles, the number of Facebook friends, the number of photo albums, and the number and types of social groups joined, presence of unprofessional content of Facebook profile (illustrating substance abuse, sexism, racism, or lack of respect for patients or others).	Most students use Facebook. However, privacy settings did not allow strangers to see many of the students' posts. Thus, when we visited their profiles as strangers, we found unprofessional content on very few profiles. However, unprofessional content, even if hidden from strangers, is still unprofessional content. Since Facebook is an integral part of our lives, it is important for medical educators and students to	Yes, approved

								understand the implications and importance of e-professionalism. Students should be explicitly instructed that patients and others might judge them based on their Facebook profiles and that they need to be conscious that their online image can affect their professional standing. Professionalism curricula should be revised to include e-professionalism.	
Nyangeni, Du Rand, Van Rooyen. 2015 [25]	To explore and describe the perceptions of nursing students regarding responsible use of SM.	Exploratory qualitative study (qualitative)	Thematic analysis of semi-structured interviews	12 nursing students	South Africa	All types of SM	Perceptions regarding the use of SM	The extensive use of SM in the clinical environment, by healthcare students, requires a joint effort by Nursing Education Institutions and healthcare facilities to ensure that SM are used in an ethically acceptable manner. The implementation of the recommendations of this research study could positively influence legally and ethically acceptable use of SM at healthcare facilities.	Yes, approved
Gettig, Noronha, Graneto, Obucina, Christensen, Fjortoft. 2016 [26]	To compare pharmacy, osteopathic medicine, dental medicine, and physician assistant (PA) students' perceptions of e-professionalism.	Cross-sectional survey (quantitative)	Descriptive analysis	208 pharmacy students, 208 osteopathic medicine students, 132 dental students, 87 physician assistant students (RR 94,2%)	USA	All types of SM	16 scenarios in which a hypothetical health care student or professional shared information or content electronically and students were asked to indicate how much they agreed that the scenario represented professional behavior.	The health care professions students surveyed had similar perceptions of e-professionalism. Of the four cohorts, osteopathic medicine students appeared less conservative in their approach to e-	Yes, exempted

								professionalism than the other cohorts.	
Yang, Jeong, Je, Jee, Yoo, Choi. 2016 [27]	To examine the use patterns of SNSs among pharmacy students and assess their perceptions regarding e-professionalism on popular SNSs under the newly implemented 6-year pharmacy educational system in South Korea.	Cross-sectional survey (quantitative)	Descriptive analysis	223 pharmacy students (RR 94.2%)	South Korea	All types of SM	demographic data, SNS activities, perceptions of e-professionalism	Many Korean pharmacy students use SNSs. However, the term “e-professionalism” is relatively new and unfamiliar in Korea. Faculty members and administrators of pharmacy schools in Korea should introduce new issues related with “e-professionalism” to pharmacy students and make them familiar with it before starting to seek pharmacy jobs.	Yes, approved
Chester, Walthert, Gallagher, Anderson, Stitely 2017 [28]	To address a deficit in data and knowledge regarding patient-targeted Googling (PTG), and to investigate medical student use of SNSs due to a close association with PTG.	Mixed methods (qualitative and quantitative)	i. Descriptive analysis<br><br>ii. Thematic analysis	88 medical students (RR 65,1%)<br><br>2 focus groups (10 students)	New Zealand	All types of SM	Key themes relating to students’ attitudes towards PTG, and reasons why they might engage in PTG.	PTG was uncommon. Attitudes were varied and context dependent. Most participants saw problems with PTG and favoured more explicit guidance on the issue. SNS usage was high; participants were concerned by the content of their SNS profiles and who they were connecting with online. Participants showing high SNS use were 1.83 times more likely to have conducted PTG than lower use groups.	Yes, approved
Knott, Nigel, Wassif 2018 [29]	To explore the views of the first-year graduate entry program students at the University of Central Lancashire and their use of SM together with their opinions on what they consider to be professional online behavior	Mixed methods (qualitative and quantitative)	i. Descriptive analysis<br><br>ii. Thematic analysis of semi-structured interviews	i. 22 students (RR not stated)<br><br>ii. 4 students	UK	All types of SM	demographic data, questions about the use of different social media sites, awareness of privacy settings online, views about what constitutes unprofessional behavior online, accepting patients and university staff as ‘friends’ on social network	Educators should incorporate additional details not only about professionalism and ethical and legal aspects within the undergraduate curriculum but more specific emphasis on the use of SM as part of the undergraduate BDS course	Yes, approved

West, Wagner, Greenberg, Buck, Hsieh, Horn et al. 2018 [30]	To examine the relationship between medical students' SM beliefs and behaviors and professional identity measured by a physician professional identity fusion pictorial item.	Cross-sectional survey (quantitative)	Descriptive analysis	3,473 first-, second-, third-, and fourth-year medical students (RR not stated)	USA	All types of SM	Identity fusion, physician professional identity, SM measures, perceptions of SM beliefs and behaviors.	This study suggests as students' physician identity emerges, they are more likely to expect themselves and peers to represent the larger community of physicians professionally, at least in the realm of SM. While this study focused on beliefs and behaviors related to SM, the results are encouraging for professional identity formation in general.	Yes, approved
Dobson, Patel, Neville. 2019 [31]	To determine whether students were aware of the standards set by the General Dental Council (GCD) regarding SM and whether they believed they were being professional in their online activities.	Cross-sectional survey (quantitative)	Descriptive analysis	88 dental students (RR 67%)	UK	All types of SM	Patterns of SM use, demographics, e-professionalism and GDC guidelines awareness, privacy setting and profile accessibility, self-googling.	This research has revealed certain flashpoints between student perceptions of professionalism and the standards expected of them by regulators and Achieving this ideal has become further complicated by the arrival of SM and its society alike. While students have an awareness of GDC SM guidelines, this study reveals a gap between their knowledge of the guidelines and its impact on values, attitudes and behavior. If this situation is not resolved, then students will begin to appear in the GDC's Fitness to practice cases.	Yes, approved
Hsieh, Kuo, Wang. 2019 [32]	To develop a curriculum for teaching professionalism by enabling students to share positive examples of professionalism in SM that reflects the	Mixed methods (qualitative and quantitative)	i. Descriptive analysis<br><br>ii. Content analysis	103 medical students in their first clinical year (RR not stated)	Taiwan	Facebook	Positive behaviors students observed during clinical practice	An innovative training program was well accepted in the formal curriculum and the predesigned SM environment, and it also adapts to the needs of different cultures in	Yes, approved

	authentic experience in clinical environment.							medical professionalism education. Appreciative inquiry for medical professionalism should be integrated into the organizational culture and the culture of SM interaction.	
Sadd. 2019 [33]	To (1) describe the effect of education in professional boundaries on the use and management of SM by using quantitative survey methods, to (2) ask "What are the social networking behaviors of student nurses following education in professional boundaries?	Cross-sectional survey (quantitative)	Descriptive analysis	102 nursing students (RR 45%)	New Zealand	All types of SM	Use of social networking, online profile and privacy, professionalism and social networking, demographics	Findings from this research indicate that student nurses are active SNS users, primarily for personal engagement. Facebook is overwhelmingly the most popular SNS, with Snapchat and Instagram also commonly used. While students primarily used SNS for personal reasons, many reported SNS use for educational / professional purposes as well, including to discuss academic related topics. Most students responded that they were aware of privacy settings on SNS, however there is a discrepancy between awareness of privacy settings and the number of students implementing the privacy features.	Yes, approved
Hinojo-Lucena, Aznar-Díaz, Cáceres-Reche, Romero-Rodríguez. 2020 [34]	To analyze the use and integration of social networks to favor international collaboration between medical students from the University of Granada (Spain) and the University of Sheffield (UK).	Cross-sectional survey (quantitative)	Descriptive analysis	110 medical students (RR not stated)	UK, Spain	All types of SM	Student expectations, use of ICT and assessment/satisfaction	High percentages according to the improvement of learning from collaboration. This type of experience highlights the need to collaborate from digital networks with health professionals from other countries to improve student learning and know different methods	None stated

								and systems of action that improve daily medical practice.	
Karveleas, Kyriakouli, Koukou, Koufatzidou, Kalogirou, Tosios 2020 [35]	To investigate the behaviour of Greek dental students on Facebook, focusing on potentially unprofessional posts and the online student-patient relationship.	Cross-sectional survey (quantitative)	i. Descriptive analysis	512 dental students (RR 97.7%)	Greek	Facebook	(a) demographics and general characteristics of responders; (b) Facebook profile settings and main reasons for its use; (c) Facebook content shared by dental students; (d) student-patient relationship via Facebook; and (e) students' perception about the impact of their online behavior	Dental students might fall into pitfalls when it comes to e-professionalism. As SM are becoming an integral part of life, there is need to include e-professionalism in dental education curriculum.	Yes, approved
**Deans / Program directors (n=5)**									
Marnocha, Marnocha, Pilliow. 2014 [36]	To (1) assess presence, intensity, and content areas of cyberprofessionalism incidents among nursing students and (2) gain general information about institutional responses to such issues and incidents.	Mixed methods (quantitative and qualitative)	i. Descriptive analysis<br><br>ii. Qualitative content data analysis	293 deans and directors of nursing programs (RR 26%)	USA	All types of SM	Awareness of incidents of student-posted unprofessional content online levels of concern about the posting of unprofessional content online, institutional policies and responses regarding unprofessional content, disciplinary actions	Cyberprofessionalism is an important concern for nursing professionals and educators, and it appears that unprofessional online behavior among nursing students is prevalent. Educational institutions need to be proactive by implementing SM policies and ensuring effective and timely handling of student-posted unprofessional content.	Yes, approved
Langenfeld, Vargo, Schenarts. 2016 [37]	To (1) assess the rate and manner in which general surgery program directors (PDs) currently use SM and determine if they had encounter inappropriate behavior among students, residents and faculty members, (2) determine how may residency programs had formal policies and curricula on SM within surgical education and (3) gauge the PDs current opinions	Cross-sectional survey (quantitative)	Descriptive analysis	110 surgery program directors (RR 42,5%)	USA	SM sites, with specific reference to Facebook and Twitter	SM presence, unprofessional online behavior, formal disciplinary action against resident/faculty member for online behavior; intuitional policy on SM; formal instruction for residents on SM and professionalism	Use of SM is high among PDs, and they often view the online behavior of residency applicants, surgical residents, and faculty surgeons. Within surgical education, there needs to be an increased focus on institutional policies and standardized curricula to help educate physicians on SM and online professionalism.	Yes, approved

	on the importance of SM within surgical education.								
Henry, Pieren. 2014 [38]	To evaluate how SM is being used in dental hygiene program admissions and policy.	Mixed methods (quantitative and qualitative)	i. Descriptive analysis<br><br>ii. Qualitative data analysis	155 dental hygiene program directors (RR 48,3%)	US	All types of SM	Personal use of SM, program use of SM, SM use in admissions and SM policies	SM is currently being used in a variety of ways in dental hygiene programs, but not in the area of admissions. There is some uncertainty about the role SM should play in a professional environment.	Yes, exempted
Al Qarni, Al Shehri, Wahab. 2019 [39]	To assess the attitudes of program directors toward the ranking of applicants for residency programs using their SM accounts as a criterion of acceptance.	Mixed methods (qualitative and quantitative)	i. Descriptive analysis<br><br>ii. Qualitatively via focus groups	i. 39 family medicine program directors (RR 95.12%)<br><br>ii. 10 family medicine program directors	Saudi Arabia	All types of SM	Demographics, SM usage, online background checks, investigating SM of residency applicants	Program directors agreed on the importance of SM behavior and how inappropriate behavior could jeopardize a candidate's chance of acceptance. It is vital that to formulate bioethical regulations to meet the needs of the new digital age. Culturally and nationally appropriate guidelines for the use of SM by health-care professionals are urgently required. Such bioethical guidelines will help minimize inappropriate behavior and increase patient sense of safety.	Yes, approved
Staud and Kearney. 2019 [40]	To identify how online SM behaviors, influence the licensure and enforcement practices of dental professionals.	Cross-sectional survey (quantitative)	Descriptive analysis	18 directors of the dental boards (RR 35%)	USA	All types of SM	Dental boards demographics and SM tools used, reporting and disciplinary actions for dentists, state dental board current impressions of online unprofessional behaviors	Dental boards are aware of potential online unprofessional behaviors and have implemented various consequences. Dental boards should consider developing policies to address potential online unprofessional behavior to protect the public that they serve.	Yes, exempted
Undergraduate and postgraduate HCPs (n=7)									

Jafarey, Shekhani, Mohsin-E-Azam, Gill, Shirazi, Hassan, Iqbal, Naqvi. 2016 [41]	To (1) assess the pattern of Facebook usage among a cross section of physicians, trainees, and medical students across three major Pakistani cities, (2) assess their knowledge and practice regarding privacy and content control on Facebook, including patient-related content, (3) assess professional behavior among the different cohorts on Facebook, and whether it threatened classical professional boundaries.	Mixed methods (quantitative and qualitative)	i. Descriptive analysis<br><br>ii. Thematic analysis	i. 897 medical professionals: physicians, trainees and medical students (RR 86%)<br><br>ii. 10 in-depth interviews, 3 focus groups	Pakistan	Facebook	Institutional characteristics of the participants, presence of profiles, reasons for Facebook use, professional utility of Facebook, privacy settings, possibilities of dissemination of Facebook content, photo content, informed consent and patient-related content and images, friending patients, friending across professional boundaries and professional behavior among groups	Physicians, their trainees and students are using Facebook for a variety of social and professional reasons, with contents of both kinds of discussions easily spilling over to those on their "Friends" list. With Facebook increasingly becoming a source of often unverified professional information, there is a real danger of Facebook feeds from professionals spreading disinformation, especially to nonprofessional circles where people might lack the judgment to tell fact from fiction. Despite a clear level of discomfort with online privacy, there is little evidence that respondents wished to take more control over their posted content.	None stated
Duke, Anstey, Carter, Gosse, Hutchens, Marsh. 2017 [42]	To explore faculty and student utilization of social media and its professional implications in nurse education.	Mixed methods (quantitative and qualitative)	i. Descriptive analysis<br><br>ii. Descriptive analytic approach.	i. 337 Nursing students (RR 54,5%)<br><br>ii. 74 faculty members (RR 39,2%)	Canada	All types of SM	Type, frequency and purpose of SM use, e-professionalism and privacy, perceptions of advantages and disadvantages in relation to SM use	There is a high reported usage of SM among students and faculty. Utilization of public platforms, while potentially beneficial, can have professional implications if not used appropriately with both personal and academic use. Developing best practice approaches for using social media in nurse education is essential to ensure that faculty and students are informed of e-professionalism.	Yes, approved

Garg, Pearson, Bond, Runyon, Pillow, Hopson, Cooney, Khadpe, Nomura, Inboriboon. 2016 [43]	To (1) identify and characterize the types and reported incidence of unprofessional SM behavior by EM residents, faculty, and nurses and the (2) concomitant personal and institutional risks.	Cross-sectional survey (quantitative)	Descriptive analysis	i. 772 emergency residents (RR 13%)<br><br>ii. 542 faculty members (RR 44%)	USA	All types of SM	Professional behavior, posting identifiable patient information, radiographs, clinical picture, posting images of intoxicated colleagues, inappropriate photographs or posts	EM residents and faculty members cause and encounter high-risk-to-professionalism events frequently while using SM; these events present significant risks to the individuals responsible and their associated institution. Awareness of these risks should prompt responsible SM use and consideration of Council of Emergency Medicine Residency Directors Social Media Task Force recommendation.	Yes, approved
Soares, Shenvi, Waller, Johnson, Hodgson. 2017 [44]	To compare State Medical Board (SMB) directors' perceptions of investigation for specific SM behaviors with those of emergency medicine (EM) physicians.	Cross-sectional descriptive study (quantitative)	Descriptive analysis	119 participants (7 medical students, 68 residents, and 44 attending physicians), (RR 58%)	USA	All types of SM	Unprofessional SM behaviors involving inappropriate communication, identifying patient images and discriminatory speech	Physicians reported a lower likelihood of investigation for themes that intersect with social identity, compared to SMB directors, particularly for images of alcohol and derogatory speech.	Yes, approved
Kitsis, Milan, Cohen, Myers, Herron, McEvoy, Weingarten, Grayson. 2016 [45]	To (1) examine and compare characteristics of SM use by medical students and faculty; (2) explore the scope of self- and peer-posting of unprofessional online content; (3) determine what actions were taken when unprofessional content was viewed.	Cross-sectional survey (quantitative)	Descriptive analysis	i. 496 medical students (RR 64%)<br>ii 614 faculty members (RR 23%)	USA	All types of SM	SM usage, barriers to SM usage, posting unprofessional online content, correlation of age and SM platform	Medical students reported spending more time using SM and posting unprofessional content more often than did faculty. The data suggest that medical students and faculty may have different perceptions of SM professionalism.	Yes, approved
Brisson, Fisher, LaBelle, Kozmic. 2015 [46]	To (1) examine differences in SNS usage between students and faculty and (2) evaluate four content areas: SNS usage patterns, attitudes regarding activity on SNS, experience with patient interactions online, and awareness of	Cross-sectional survey (quantitative)	Descriptive analysis	i. 300 students in school of medicine (RR 42%)<br><br>ii. 31 school of medicine faculty members (RR 78%)	USA	All types of SNS sites	SNS usage patterns (personal or professional use), attitudes regarding activity on SNS sites, experience with patient interactions online, and awareness of institutional guidelines on appropriate use of SNS sites	Students are more likely than faculty to use SNS and use it very differently than faculty. Students would benefit from training on appropriate use of SNS. Topics that should be addressed include editing one's online presence,	Yes, exempted

	institutional guidelines on use of SNS.							managing friend requests from patients, dealing with colleagues who post harmful content, conducting Internet searches on patients, and discussion of boundaries to identify potential harms associated with SNS usage.	
Pereira et al. 2015 [47]	To develop evidence for the professional use of SM in medicine.	Qualitative framework	Thematic analysis of appreciative inquiry	30 self-selected participants (workshop group session) 19 attending physicians; 5 resident doctors; 2 non-physician educators; 2 administrators; 1 librarian; and 1 medical student.	Canada	All types of SM	Discovery of best practices, the most positive trends, events and developments on SM and professionalism, future plans regarding SM and professionalism, defining areas for improvement of digital professionalism, ensuring implementation of professionalism in SM through changes.	Appreciative inquiry is a tool that can develop the positive practices of organizations and individuals. Study results provide evidence for the professional use of SM that may contribute to guidelines to help individuals realize benefits and avoid harm.	Yes, approved
**Postgraduate HCPs (n=41)**									
D'Souza, Henningham, Zou, Huang, O'Sullivan, Last et al. 2017 [48]	To (1) assess attitudes of health educators toward SM use in education, (2) examine differences between faculty members who do and do not use SM in teaching practice, and (3) determine contributing factors for an increase in the uptake of SM	Cross-sectional survey (quantitative)	Descriptive analysis	270 faculty members (nursing, public health, medicine, pharmacy, dentistry, and physiotherapy) (RR 4%-46%)	China, Mexico, UK, Canada, Ireland, Hong Kong, Australia	All types of SM	Usage of SM, relationship between the frequency of SM use and barriers to the use of SM for health education, factors influencing decisions to use SM in teaching practice, capacity of SM to improve interactions among students/educators, and the type of SM currently used, frequency of SM use in educational practice "users" and "nonusers"	Usage of SM as teaching tool isn't uniform for all faculty members but necessitates targeted strategies for current users and nonusers. Users were more motivated by learner satisfaction and deterred by lack of technology compatibility, whereas nonusers reported the need for departmental and skill development support. Both shared concerns of professionalism and lack of evidence showing enhanced learning. The majority of educators are open-minded to incorporating SM into their teaching practice.	Yes, approved

Kesselheim, Schwartz, Belmonte, Boland, Poynter, Batra, et al. 2016 [49]	To (1) describe pediatric residents' experiences related to social networking sites (particularly Facebook &Twitter), and to (2) compare survey results to previously reported data from pediatric program directors, also to (3) explore how these data may shape future educational interventions related to residents' use of these technologies	Cross-sectional survey (quantitative)	Descriptive analysis	495 pediatric residents (RR 52%)	USA	SNS (particularly Facebook and, Twitter)	Familiarity with and use of SNS, perceptions of resident professionalism on SNS, inappropriate behavior on SNS, educational interventions or policies about SNS	Residents were significantly more likely than program directors to report the existence of educational interventions related to SM their programs. Effective educational interventions for teaching online professionalism must include the skills necessary for residents not only to recognize inappropriate behavior on SNS but also learn how to address it themselves.	Yes, approved
Langenfeld, Cook, Sudbeck, Luers, Schenarts. 2014 [50]	To (1) evaluate the publicly available Facebook profiles of surgical residents and to (2) determine the incidence and degree of unprofessional conduct.	Mixed methods (qualitative and quantitative)	i. Content analysis of Facebook profiles<br><br>ii. Descriptive analysis	319 surgical residents (RR 32%)	USA	Facebook	Professional, potentially unprofessional, or clearly unprofessional content on publicly available profiles (binge drinking, sexually suggestive photos)	Unprofessional behavior is prevalent among surgical residents who use Facebook, and this behavior does not appear to decrease as residents' progress through training.	Yes, approved
Scragg, Shaikh, Shires, Stein Hodgins, Mercer, Robinson, et al. 2017 [51]	To (1) explore breast screening practitioners' opinions and attitudes with regard to using SM, (2) engage with clients online, (3) identify challenges, and (4) strategies to overcome them, and (5) make recommendations for further work	Qualitative exploratory nature	Thematic analysis	78 participants in 4 workshops (RR not stated)	UK	All types of SM	Professional and legal accountability, information accuracy, access, skills and training, supportive policies regarding SM use, privacy settings/sharing, online habits	Breast screening practitioners appeared willing and motivated to engage in SM not only to benefit their clients but also to improve their own professional networking and development. Participants identified a need to undergo training to develop their skills for communicating effectively online.	Yes, approved
Laliberté, Beaulieu-Poulin, Campeau Larrivée, Charbonneau, Samson, Ehrmann Feldman. 2016 [52]	To explore knowledge and behavior that physiotherapists and physical rehabilitation therapists practicing in Quebec have of Facebook.	Cross-sectional survey (quantitative)	Descriptive analysis	322 physiotherapy professionals (physiotherapists and physical rehabilitation therapists) (RR 4,5%)	Canada	Facebook	Personal and professional use of Facebook, experience with Facebook, knowledge of platform's privacy policies	Need to establish guidelines regarding the use of SM for physiotherapy professionals to ensure maintenance of professionalism and ethical conduct.	Yes, approved

Fuoco, Leveridge. 2015 [53]	To understand the attitudes and practices of urologists regarding SM use.	Cross-sectional survey (quantitative)	Descriptive analysis	229 urologists (RR 45.4%)	Canada	All types of SM	Use of SM, attitudes toward SM, interaction with patients on SM sites, guidelines or legislation about SM use, unprofessional posts	Practicing urologists engage infrequently in SM activities and are almost universal in avoiding SM for professional use. Most feel that SM is best kept to exchanges between colleagues. Emerging data suggest an increasing involvement is likely in the continuing professional development space.	Yes, approved
Irfan, Farhana, Eiad, Nassr, Al Mohammed, Maya N et al. 2018 [54]	To evaluate the utility of SM among family medicine residents and consultants.	Cross-sectional descriptive study (quantitative)	Descriptive analysis	40 family medicine physicians and 92 residents (RR 78%)	Kingdom of Saudi Arabia	All types of SM	SM use (presence, reasons, frequency), attitudes/perception of the benefit, usefulness and quality of information of SM	SM was very popular among physicians and residents. Females and physicians used SM more for professional purposes than did residents. Male physicians used SM less for educational and learning purposes, and instead used it for personal reasons and leisure. Majority found it to be an effective use of time, and a beneficial and engaging way to get high-quality information	Yes, approved
Panahi, Watson, Partridge. 2016 [55]	To identify approaches through which physicians establish interpersonal trust on SM	Exploratory qualitative study (qualitative)	Thematic analysis of semi-structured interviews	24 physicians (RR not stated)	Australia, USA, Europe	All types of SM	SM use (experience, trust), trustworthiness on SM	Healthcare professionals need to approach SM carefully when using it for knowledge sharing, networking and developing trusted relations with like-minded peers.	Yes, approved
Khandelwal, Nugus, Elkoushy, Cruess, Cruess, Smilovitch, et al. 2015 [56]	To create and evaluate a teaching format reflecting the complexity of the clinical environment and incorporating the contemporary issue of SM use, while maximizing the limited time available for	Participatory action research (mixed methods, qualitative and quantitative)	i. Descriptive analysis of pre- and post-workshop survey<br><br>ii. Qualitative analysis of workshop participants' feedback	52 residents (various medical specialties) (RR 71,2%)	Canada	All type of SM	E- professionalism, attributes of professionalism in clinical context, social contract between physicians and society, understanding of how the faculty of medicine code of conduct fits into professionalism	Contemporary issues such as SM have an impact on the professionalism of postgraduate trainees and physicians, and thus must be addressed in the professionalism curriculum.	Yes, approved

	professionalism teaching for residents.								
Koo, Bowman, Ficko, Gormley. 2018 [57]	To characterize changes in the frequency and nature of unprofessional content on urologists' Facebook accounts during the transition from residency to practice.	Cohort mixed methods study (qualitative and quantitative)	i. Content analysis of FB profiles and posts<br><br>ii. Descriptive analysis	198 urologists (RR 70%)	USA	Facebook	Publicly identifiable accounts, unprofessional or potentially objectionable content, changes in content during time	Most urologists maintained public Facebook accounts after the transition to practice, and about half of these contained unprofessional or potentially objectionable content. Amidst their increasing self-identification as urologists on SM, the majority of practicing urologists had posted concerning content, which could have an impact on their professional identities and public perceptions of the specialty.	Yes, approved
Dawkins, King, Boateng, Nichols, Desselle. 2017 [58]	To (1) understand what pediatric residents view as appropriate SM postings,<br>(2) recognize the degree to which these residents are exposed to postings that violate SM professionalism guidelines	Cross-sectional descriptive study (quantitative)	Descriptive analysis	1,628 pediatric residents<br>(RR 16,53%)	USA	Facebook	Frequency of Facebook use, appropriateness of the posting, awareness of institutional policies, prior SM training	Today's residents, like others of their generation, use SM sites to converse with peers without considering the implications for the profession. The frequent use of SM by learners needs to change the emphasis educators and regulatory bodies place on SM guidelines and teaching professionalism in the digital age.	Yes, exempted
Benetoli, Chen, Schaefer, Chaar, Aslani. 2016 [59]	To explore how pharmacists separate professional and personal information and activities on SNS, their perceptions of professional behavior on	Exploratory qualitative study (qualitative)	Thematic analysis of semi-structured interviews	31 international practicing pharmacists (RR not stated)	Australia, New Zealand, United States, Brazil, Germany, Nigeria,	SNS	Content of SNS posts, identification of professional and personal information on SNS, capabilities to recognize and distinguish private from professional information	A definitive strategy to separate professional and personal information and activities on SNS was not adopted by most pharmacists. E-professionalism issues could negatively impact	Yes, approved

	SNS, and opinions on guidelines in this area.				Thailand, Philippines, United Kingdom			on the standing of individual practitioners and the profession.	
Levati. 2014 [60]	To explore the use of Facebook by Registered Nurses (RNs) in Italy and the United Kingdom (UK), focusing on the disclosure of personal and professional information.	Mixed methods cross-sectional survey (quantitative and qualitative)	i. Descriptive analysis<br>ii. Content analysis	124 nurses' profile pages (RR 60%)	UK, Italy	Facebook	Privacy settings, the nature of material posted by nurses, content of "wall page" and pictures	RNs in Italy and in the UK had an online behavior that would not cause concern, but the study also identified certain online behaviors, which may put nurses at a higher level of vulnerability and lead to the disclosure of potentially unprofessional conduct. Relevant nursing governing bodies should promote further discussion at national and international level.	Yes, approved
Patel, Hawkins, Rawson, Hoang. 2017 [61]	To compare radiologists who use SM for professional purposes to those who do not regarding their characteristics, habits, and attitudes.	Cross-sectional descriptive study (quantitative)	Descriptive analysis	186 radiologists' complete responses, 124 (66.7%) responded to the e-mailed survey and 62 (33.3%) responded to SM promotion (RR for e-mail survey 75.2%, RR for SM promotion not stated)	USA	Facebook and Twitter	SM presence, type of SM user, patterns of use (platforms, duration, frequency), benefits and barriers of SM use	Nearly 60% of radiologist respondents use SM for professional purposes. Radiology is likely to see growth in the number of users and in the role of SM in the coming years as nearly half of professional users are radiology trainees. Twitter use for professional purposes among radiologists was disproportionately male. It is important to be cognizant of gender imbalance and to improve visibility of female leaders on social networking.	Yes, approved
Wagner, Cochran, Jones, Gusani, Varghese Jr., Attai. 2018 [62]	To characterize surgeon's professional use and perceptions of SM	Cross-sectional descriptive study (quantitative)	Descriptive analysis	208 surgeons, clinical fellows, and residents (RR 20%)	USA	All types of SM	Preferred SM for professional contact, attitude toward SM, effect of SM on professional development	Most of surgeons responding to survey used some form of SM for professional purposes and consider SM participation potentially beneficial to professional	Yes, approved

								development. Generational differences in surgeon attitudes suggest usage of SM among surgeons will expend over time.	
Adilman, Rajmohan, Brooks, Urgoiti, Chung, Hammad et al. 2016 [63]	To (1) address the current lack of knowledge surrounding the nature of Web-based SM use by oncology physicians and physicians in training, to (2) identify gaps in SM use between age-defined physician generations, and to (3) perform a needs assessment by identifying what physicians seek through SM and areas for improvement	Prospective cohort survey study (quantitative)	Descriptive analysis	207 participants (oncology physicians) (RR 30.4%).	Canada	All types of SM	Frequency, purpose, and platform of SM use, Attitudes toward SM use benefits and barriers	The identified gap in SM use between age cohorts may have negative implications for communication in oncology. Despite advancements in SM and efforts to integrate SM into medical education, most oncologists and trainees use SM rarely, which, along with the age-related gap in use, may have consequences for collaboration and education in oncology. Investigations to further understand barriers to SM use should be undertaken to enhance physician collaboration and knowledge sharing through SM.	None stated
Neville. 2017 [64]	To (1) explore the prevalence of SM related Fitness to Practice (FtP) cases investigated by the General Dental Council (GDC) from 1 September 2013 to 21 June 2016, (2) quantitatively examine the nature of each cases and identify pertinent themes and underlying patterns to these online professional lapses.	Case study design - mixed methods (qualitative and quantitative)	Content analysis framework	6 cases – dental doctors (2,4%)	UK	All types of SM	Incidence of SM Fitness to Practice (FtP) cases investigated by the GDC	This analysis of FtP cases relating to the GDC's SM guidelines supports the assumption that SM can be a vehicle for unprofessionalism. The low incidence rate should be interpreted with caution, being illustrative of the types of issues that might arise rather than the volume.	None stated

Klee, Covey, Zhong. 2015 [65]	To (1) provide insight into family physicians' use and acceptance of SM as well as (2) assess current professional SM training.	Cross-sectional descriptive study (quantitative)	Descriptive analysis	61 family medicine residents and 192 practicing physicians responded (RR 21%.)	USA	All types of SM	SM use (presence, reasons, frequency), the patient–physician relationship ("friending" patients), SM knowledge and training	The high utilization of SM by younger providers, high prevalence of patient use of the Internet, and the countless beneficial opportunities SM offers should be catalysts to drive curriculum development and early implementation in medical education. This curriculum should focus around four pillars: professional standards for SM use, SM clinical practice integration, professional networking, and research.	Yes, approved
Mohiuddin, Shahid, Shuaib. 2015 [66]	To (1) measure resident awareness of SM and to (2) evaluate the impact of reflective practice-based sessions regarding the impact of SM on professionalism in surgery	Prospective observational study (quantitative)	Descriptive analysis of pre/post session surveys	i. 38 surgical residents (RR 79 %)<br><br>ii. 28 surgical residents (RR 74%)	Dominican Republic	All types of SM	Frequency of SM usage, "friending"on SM, unprofessional posts and content, impact of SM on professional career, protection of patient information on SM	Reflective practice-based sessions regarding the impact of SM on professionalism in surgery were well favored by the residents. The majority agreed that it had important implications for the longevity of their professional career. Participants reported having an increased awareness to protect patient privacy and utilize SM more professionally.	Yes, approved
Lefebvre et al. 2016 [67]	To (1) investigate the existing perceptions about SM and professionalism among new physicians entering in professional clinical practice. (2) determine the effects of formal SM instruction and policy on young professionals' ability to navigate case-based scenarios about online behavior in the	Prospective observational study (quantitative)	Descriptive analysis	70 residents (9 specialties)<br>(RR 56%)	US	Facebook	Self-reported instruction on SM use in medical school; self-reported familiarity with current institutional SM policy; case-scenario questions regarding specific tenets of the current institutional SM policy; understanding of SM account control options; prior SM account closures; prior unprofessional posts; current SM use	Young physicians demonstrate a casual approach to SM activity in the context of professional medical practice. However, SM instruction and/or familiarity with the SM policy are associated with more cautious perceptions about online behavior. Furthermore, assessment of	Yes, approved

	context of professional medicine							perceptions and practices of new employees in a health care environment may help improve the content and delivery of policy information.	
Surani, Hirani, Elias, Quisenberry, Varon, Surani S et al. 2017 [68]	To evaluate the use of SM among healthcare workers in an attempt to identify how it affects the quality of patient care.	Cross-sectional descriptive (exploratory) study (quantitative)	Descriptive analysis	366 healthcare providers (RR 99%)	USA	All types of SM	SM use (presence, frequency), existence of institutional SM policy, comparison on the use of SM between physicians and nurses	The use of SM in the study was in line with the general public. This study suggests that physicians contribute to medical forums online more than nurses.	Yes, approved
Campbell, Evans, Pumper, Moreno. 2016 [69]	To understand perspectives and experiences of these “early adopter” physician bloggers and SM users.	Exploratory qualitative study	Thematic analysis of in-depth interviews	17 physicians (RR not stated)	USA	All types of SM	Experiences with using SM for health information, including benefits and challenges, institution support, and perceptions and experiences of their online audiences	Uncertainty remains regarding roles and responsibilities of physicians providing medical content within SM forums and few providers appeared to be using the platform to its full potential.	Yes, exempted
Alshakhs, Alanzi. 2018 [70]	To evaluate the perception of health-care professionals in Saudi Arabia toward the usages of SM in health-care delivery	Cross-sectional descriptive study (quantitative)	Descriptive analysis	120 health-care professionals (RR not stated)	Kingdom of Saudi Arabia	All types of SM	Pattern of SM usage, perception of SM benefits and risks	The results of this research indicate that SM can be a useful tool by which physicians may promote their services and publish general health information. However, there are potential problems in the use of social networks that can have negative consequences for patients and HCPs. This implies that precautions must be taken to avoid ruptures of patient privacy and other risks that can result in legal action against health professionals damaging their image and professional status. The study also found that the participants are willing to	Yes, approved

								use SM for professional purposes	
Langenfeld et al.<br>2015 [71]	To (1) evaluate the publicly available Facebook profiles of faculty surgeons involved in the education of general surgery residents, and (2) determine the incidence and degree of unprofessional behavior exhibited by them on Facebook.	Mixed methods (qualitative and quantitative)	i. Content analysis of Facebook profiles<br><br>ii. Descriptive analysis	195 faculty in the practice of general surgery (25.7%)	United States	Facebook	Professional, potentially unprofessional, or clearly unprofessional content on publicly available profiles	Unprofessional behavior on Facebook is less common among surgical faculty compared with surgical residents. However, the rates remain unacceptably high, especially among men and those in practice for less than 5 years. Education on the dangers of SM should not be limited to residents but should instead be extended to attending surgeons as well.	Yes, approved
Weijs et al.<br>2017 [72]	To (1) explore attitudes, beliefs, and experiences of Canadian public health professionals (PHPs) and their personal use of Facebook to (2) assess views of online professionalism and (3) blurring between their professional and personal lives.	Cross-sectional survey (quantitative)	Descriptive analysis	621 public health professionals<br>(RR not stated)	Canada	Facebook	Facebook usage, information disclosure, privacy and default settings knowledge, awareness of the consequences, personality factors (need for popularity, self-esteem, trust), on-line professionalism	Overlap between the private and public lives of Canadian PHPs exists on Facebook and highlights the potential for damage to public health credibility.	Yes, approved
Koo, Ficko, Gormley.<br>2017 [73]	To characterize unprofessional content on public Facebook accounts of contemporary US urology residency graduates	Mixed methods (quantitative and qualitative)	i. Content analysis of Facebook profiles<br><br>ii. Descriptive analysis	201 urology residency graduates (72%)	United States	Facebook	Assessment of unprofessional content on Facebook (unprofessional content, potentially objectionable content)	Most recent residency graduates had publicly accessible Facebook profiles, and a substantial proportion contained self-authored unprofessional content. Of those identifying as urologists on Facebook, approximately half violated published professionalism guidelines. Greater awareness of trainees’	Yes, approved

								online identities is needed.	
Borgmann, DeWitt, Tsaur, Heferkamp, Loeb. 2015 [74]	To assess the perceived utility of Twitter for professional goals and the perceived impact of Twitter on clinical practice of active Twitter physician users. We aimed to determine the perceptions of the impact of Twitter on users' clinical practice, research, and other professional activities.	Cross-sectional survey (quantitative)	Descriptive analysis	A total of 312 physicians responded to the survey, including 57 during the pilot phase at the 2014 EAU meeting (RR 7.2% of all #eua14 Twitter users), and 255 of 1,199 participants from the 2014 AUA Twitter feed (RR 21.3%).	Global	All types of SM	Demographics, Twitter usage, benefits of Twitter, impact of Twitter on their clinical practice and other professional goals	Our survey suggests that most Twitter users at urology meetings do perceive numerous important benefits of SM use for networking, disseminating research, advocacy, clinical practice, and other professional goals. In addition, our study provides a proof of principle demonstration on the feasibility of using directed tweets to perform targeted survey research within the urological community.	None stated
Robertson, Shoss, Broom. 2016 [75]	To develop a SM training program that emphasized perspective taking and fostered appropriate SM use.	Mixed methods (qualitative and quantitative)	Descriptive analysis	16 pediatric residents (RR not stated)	USA	All types of SM	SM knowledge, attitudes and behaviors.	SM training module is an effective and useful tool for members of the medical community as the internet and SM continue to grow in popularity and lines between professional and personal realms are continually blurred.	Yes, approved
Duymuş, Karadeniz, Şükür, Atiç, Zehir, Azboy. 2017 [76]	To identify the prevalence of SM and Internet usage of orthopedists and to determine its effects on patient–physician communication.	Cross-sectional survey (quantitative)	Descriptive analysis	2,597 Orthopedists (RR 12,4%)	Turkey	All types of SM	The questionnaire consists of a total 25 items pertaining to personal information, which SM tool they use, their overall views of and expectations from SM, the effects of SM on patient–physician relationship and communication.	SM tools and Internet are commonly used by orthopedists to communicate with their patients. Even though there are beneficial effects in patient–physician relationship, effective standards and regulations should be developed to enable a safe communication and to resolve ethical and legal uncertainties.	None stated

Long, Qi, Ou, Zu, Cao, Zeng, et al. 2017 [77]	To evaluate SM use among Chinese urologists and the perceived impact of SM on their practice.	Cross-sectional survey (quantitative)	Descriptive analysis	Urologists<br>A total of 157, 242 and 266 survey questionnaires were completed, (RRs of 78.5%, 80.7% and 83.1% during the years 2014, 2015 and 2016, respectively).	China	All types of SM	SM usage, demographic information	Dramatic increase in SM use among Chinese urologists, provides great opportunities for online academic communication and medical education. Unprofessional use of SM in the medical practice may bring about potential risks and challenges for the further development of SM in medical practice.	Yes, approved
Nikiphorou, Studenic, Ammitzbøll, Canavan, Jani M, Ospelt, Berenbaum. 2017 [78]	To explore perceptions, barriers and patterns of SM (SM) use among rheumatology fellows and basic scientists.	Cross-sectional survey (quantitative)	Descriptive analysis	233 Rheumatology fellows and basic scientists (RR not stated)	47 countries	All types of SM	General demographics, frequency and types of SM use, reasons and barriers to SM use	Substantial use of SM by rheumatologists and basic scientists for social and professional reasons. The survey highlights a need for providing learning resources and increasing awareness of the use of SM.	None stated
Hazzam, Lahrech. 2018 [79]	To understand the factors that relate to the frequency use of SM in the health care discipline. It also aims to explore the underlying online behaviors of HCPs, which include the exchange of medical information with peers, interpersonal communication, and productivity enhancement in their daily practice.	Cross-sectional survey (quantitative)	Descriptive analysis	973 participants that included physicians, pharmacists, and allied HCPs (RR 20,3%)	United Arab Emirates	All types of SM	Attitudes toward usage of SM; perceived usefulness, ease of use, and environmental constraint; perceived norms, image, and perceived control; intention of use and the frequency of use	The use of online platforms facilitates the exchange of medical information among peers and enhances the share of experiences that support HCP's learning and development.	None stated
Justinia, Alyami, Al-Qahtani, Bashanfar, El-Khatib et al. 2019 [80]	To assess the perceptions and usage of SM (SM) by OSs (OS) in Jeddah, Saudi Arabia, and its impact on their profession, and to evaluate their level of awareness of the potential risks on their practice	Mixed methods (qualitative and quantitative)	i. Descriptive analysis<br><br>ii. Thematic content analysis/grounded theory analysis	i. 165 orthopedic surgeons (RR 51%)<br><br>ii. 8 participants in interview	Saudi Arabia	Twitter, Facebook, Snapchat, Instagram, LinkedIn, Periscope, YouTube	i. demographics, use of SM in practice, attitudes towards SM and its use, perceptions of the associated risks<br><br>ii. attitudes towards SM, their opinions on its use, thoughts on the associated risks	OSs' perceptions and usage were generally cautious. The major concerns were patient confidentiality, along with ethical and legal consequences. The need to contribute to quality online content was evident. Lack of formal	Yes, approved

								guidelines was a continuous theme.	
Lee, Hwang, Lee, Woo, Hahn, Koh 2019 [81]	To identify Internet use patterns of psychiatrists and psychiatry residents in South Korea and to provide basic data for developing e-professionalism.	Cross-sectional survey (quantitative)	Descriptive analysis	79 Psychiatry residents<br>116 Psychiatrists (RR 78%)	South Korea	All types of SM	Demographics, use of email, web searches, personal and professional use of websites and social networking, and negative and positive experiences of electronic communication and social networking.	In order to reduce the negative effects of electronic communication and social networking, we need guidelines that are appropriate for the situation in South Korea. Furthermore, future research will need to identify and suggest solutions for negative experiences of electronic communication and social networking that may affect the relationship between patients and physicians.	Yes, approved
Renew, Ladlie, Gorlin, Long. 2019 [82]	To better delineate the role residency program-based SM accounts in recruiting prospective candidates	Cross-sectional survey (quantitative)	Descriptive analysis	89 Anesthesiology resident candidates (RR 40,6%)	USA	All types of SM	Demographics, SM use and Impact.	Program-specific SM accounts can be used to advertise and showcase their departments while offering applicants another vehicle to research programs. As more information becomes available for both program leadership and applicants through SM, care must be taken when posting content to ensure appropriate levels professionalism are maintained.	Yes, approved
Wang, Wang, Zhang, Jiang. 2019 [83]	To understand the SM use and online professionalism of Chinese registered nurses.	Cross-sectional survey (quantitative)	Descriptive analysis	1,100 Nurses (RR 60%)	China	All types of SM	Demographic and professional information, SM use and online professionalism	SM are prevalent among Chinese nurses and the most popular platform is WeChat. Chinese nurses used SM privately and professionally. SM were also used to obtain and share medical knowledge. Patient-nurse interactions via SM were quite common. Chinese nurses'	Yes, approved

								professionalism was challenged. This study also highlighted the need for Chinese guidelines regarding nurses’ SM usage.	
Ahmad, Sattar, Akram. 2020 [84]	To evaluate the quality and accuracy of professionalism videos currently available on the YouTube.	Mixed methods (qualitative and quantitative)	Descriptive content analysis of YouTube videos	17 YouTube videos on professionalism	Global (English speaking)	YouTube	Total N of: Views, Likes, Dislikes, Positive Comments, Negative Comments Per day N: Views, Likes, Dislikes, Positive Comments, Negative Comments, Like/Viewers, Dislike/Viewers, Positive comments/Like comments/Dislike, Videos scores = ((Like-Dislike)/(Like + Dislike)) * 100. Videos merits = ((Positive comments-Negative comments) * 100.	Medical professionalism multimedia videos uploaded by healthcare specialists or organizations on YouTube provided reliable information for medical students, healthcare workers and other professionals. We conclude that YouTube is a leading and free online source of videos meant for students or other healthcare workers, yet the viewers need to be aware of the source prior to using it for training and learning.	None stated
Kerr, Booth, Jackson. 2020 [85]	To identify the characteristics and behaviors of microcelebrity nurses who act as influencers on Instagram and use their nursing profile to gain attention and presence on the Web	Exploratory qualitative study (qualitative)	Thematic content analysis	10 micro-celebrity nurses Instagram profiles (RR not applicable)	Canada	Instagram	5 themes emerged: (1) engaging Instagram users, (2) educational opportunities and insights, (3) nursing-related humor, (4) emotions experienced by nurses, and (5) media and narratives including patient details or work context.	Nursing practice policies and guidelines must be updated to include recommendations pertinent to nurses with microcelebrity status, to maintain nurses’ professionalism and to protect patients’ safety.	Yes, exempted
Loo, Wong, Lee. 2020 [86]	To explore the considerations and possible competing or conflicting considerations of faculty and residents in the National Healthcare Group – Alexandra Health Pte Ltd (NHG-AHPL) Residency;	Mixed methods (qualitative and quantitative)	i. Content analysis on Facebook profile<br><br>ii. Descriptive analysis	64 Faculty<br>118 Residents (RR=36,8%)	Singapore	Facebook	Randomly presented Facebooks posts as part of an online questionnaire, rated their appropriateness, and provided explanations for their ratings.	Doctors within the same Residency do not necessarily have a uniform set of professional priorities and may have to manage conflicting professional and personal values in different contexts.	Yes, approved

Low, Tan, Joseph.2020 [87]	To determine the profile of the knowledge and practices of doctors in our institution, and to identify knowledge gaps in the use of SM accounts.	Cross-sectional survey (quantitative)	Descriptive analysis	119 Medical doctors (RR 12,8%)	Singapore	All types of SM	Demographics, use of SM, and case-based scenarios involving professionalism, patient-doctor relationship and personal practices of SM use.	Knowledge of institutional SM policy and privacy settings of SM accounts is inadequate among doctors. Regarding practices in SM use, while most agree that caution should be exercised for online posts involving patients, ambiguity still exists. The emerging knowledge deficit and potentially unsafe practices that are identified can be addressed through continuing medical education and training on SM use.	Yes, approved
Ruan, Yilmaz, Lu, Lee, Chan. 2020 [88]	To define the properties and development of the digital self and its interactions with the current professional identity development theory.	Exploratory qualitative study (qualitative)	Thematic analysis of semi-structured interviews	17 SM experts (RR not stated)	Canada	All types of SM	Demographics, frequencies of SM platforms for personal and professional use, themes (initial formation of the digital identity; cultivating digital identity; real-life identity vs. digital identity management; the professional and personal dimensions of SM)	The formation of digital identity, its development, and interactions that require identity management were features captured in our study. Moreover, the fluid and dynamic characteristics of digital identity in conjunction with its accelerated capacity of growth yield differences from professional identity development that can potentially be harnessed. Navigating the identity development of young or upcoming health care professionals is a priority for institutions now and in the post–COVID-19 world. Today, digital identity can no longer be neglected. Digital citizenship can no longer be ignored as a key	Yes, approved

								facet of one's professional responsibility.	

References:

1. Chretien KC, Tuck MG, Simon M, Singh LO, Kind T. A Digital Ethnography of Medical Students who Use Twitter for Professional Development. J Gen Intern Med. 2015 Nov;30(11):1673–80. PMID: 25952652. doi: 10.1007/s11606-015-3345-z.
2. Marnocha S, Marnocha M, Cleveland R, Lambie C, Limberg CY, Wnuk J. A Peer-Delivered Educational Intervention to Improve Nursing Student Cyberprofessionalism. Nurse Educ. 2017 Oct;42(5):245–9. PMID: 28252547. doi: 10.1097/NNE.0000000000000368.
3. Nason KN, Byrne H, Nason GJ, O'Connell B. An assessment of professionalism on students' Facebook profiles. Eur J Dent Educ. 2018 Feb;22(1):30–3. PMID: 27735108. doi: 10.1111/eje.12240.
4. Flickinger TE, O'Hagan T, Chisolm MS. Developing a Curriculum to Promote Professionalism for Medical Students Using Social Media: Pilot of a Workshop and Blog-Based Intervention. JMIR Med Educ. 2015 Dec 1;1(2):e17. PMID: 27731846. doi: 10.2196/mededu.4886.
5. George DR, Navarro AM, Stazyk KK, Clark MA, Green MJ. Ethical quandaries and Facebook use: How do medical students think they (and their peers) should (and would) act? AJOB Empir Bioeth. 2014;5(2):68–79. doi: 10.1080/23294515.2013.864344.
6. Henning MA, Hawken S, MacDonald J, McKimm J, Brown M, Moriarty H, et al. Exploring educational interventions to facilitate health professional students' professionally safe online presence. Med Teach. 2017 Sep;39(9):959-66. PMID: 28562145. doi: 10.1080/0142159X.2017.1332363.
7. Nicolai L, Schmidbauer M, Gradel M, Ferch S, Antón S, Hoppe B, et al. Facebook Groups as a Powerful and Dynamic Tool in Medical Education: Mixed-Method Study. J Med Internet Res. 2017 Dec 22;19(12):e408. PMID: 29273572. doi: 10.2196/jmir.7990.
8. Ferguson C, DiGiacomo M, Saliba B, Green J, Moorley C, Wyllie A et al. First year nursing students' experiences of social media during the transition to university: a focus group study. Contemp Nurse. 2016 Oct;52(5):625–35. PMID: 27334672. doi: 10.1080/10376178.2016.1205458.
9. Ness GL, Sheehan AH, Snyder ME. Graduating student pharmacists' perspectives on e-professionalism and social media: qualitative findings. J Am Pharm Assoc (2003). 2014 Mar-Apr;54(2):138–43. PMID: 24632929. doi: 10.1331/JAPhA.2014.13188.
10. Bagley JE, DiGiacinto D, Lawyer J, Anderson MP. Health Care Students Who Frequently Use Facebook Are Unaware of the Risks for Violating HIPAA Standards: A Pilot Study. J Diagn Med Sonogr. 2014;30(3):114–20. doi: 10.1177/8756479314530509.
11. Barnable A, Cunning G, Parcon M. Nursing Students' Perceptions of Confidentiality, Accountability, and E-Professionalism in Relation to Facebook. Nurse Educ. 2018 Jan/Feb;43(1):28–31. PMID: 28857955. doi: 10.1097/NNE.0000000000000441.
12. Rocha PN, de Castro NA. Opinions of students from a Brazilian medical school regarding online professionalism. J Gen Intern Med. 2014 May;29(5):758–64. PMID: 24395103. doi: 10.1007/s11606-013-2748-y.
13. Alkhateeb F, Alameddine S, Attarabeen O, Latif DA, Osolin S, et al. Pharmacy students' use of social media sites and perception toward Facebook use. Archives of Pharmacy Practice. 2015;6(4):77-84. doi: 10.4103/2045-080X.165134

14. Mawdsley A. Pharmacy students' perceptions of social media in education. Pharmacy Education. 2015;15(1):108–10.
15. Mather C, Cummings E, Nichols L. Social Media Training for Professional Identity Development in Undergraduate Nurses. Stud Health Technol Inform. 2016;225:344–8. PMID: 27332219.
16. Kenny P, Johnson IG. Social media use, attitudes, behaviours and perceptions of online professionalism amongst dental students. Br Dent J. 2016 Nov 18;221(10):651–5. PMID: 27857111. doi: 10.1038/sj.bdj.2016.864.
17. Jawaid M, Khan MH, Bhutto SN. Social network utilization (Facebook) & e-Professionalism among medical students. Pak J Med Sci. 2015 Jan-Feb;31(1):209-13. PMID: 25878645. doi: 10.12669/pjms.311.5643.
18. Gomes AW, Butera G, Chretien KC, Kind T. The Development and Impact of a Social Media and Professionalism Course for Medical Students. Teach Learn Med. 2017 Jul-Sep;29(3):296–303. PMID: 28272900. doi: 10.1080/10401334.2016.1275971.
19. Walton JM, White J, Ross S. What's on YOUR Facebook profile? Evaluation of an educational intervention to promote appropriate use of privacy settings by medical students on social networking sites. Med Educ Online. 2015 Jul 20;20:28708. PMID: 26198434. doi: 10.3402/meo.v20.28708.
20. Barlow CJ, Morrison S, Stephens HO, Jenkins E, Bailey MJ, Pilcher D. Unprofessional behaviour on social media by medical students. Med J Aust. 2015 Dec 14;203(11):439. PMID: 26654611.
21. Mostaghimi A, Olszewski AE, Bell SK, Roberts DH, Crotty BH. Erosion of Digital Professionalism During Medical Students' Core Clinical Clerkships. JMIR Med Educ. 2017 May 3;3(1):e9. PMID: 28468745. doi: 10.2196/mededu.6879.
22. O'Sullivan E, Cutts E, Kavikondala S, Salcedo A, D'Souza K, Hernandez-Torre M, Anderson C, Tiwari A, Ho K, Last J. Social Media in Health Science Education: An International Survey. JMIR Med Educ 2017 Jan 4;3(1):e1. PMID:28052842
23. Avcı K, Çelikden SG, Eren S, Aydenizöz D. Assessment of medical students' attitudes on social media use in medicine: a cross-sectional study. BMC Med Educ. 2015 Feb 15;15(1):18. PMID: 25890252. doi: 10.1186/s12909-015-0300-y
24. Gupta S, Singh S, Dhaliwal U. Visible Facebook profiles and e-professionalism in undergraduate medical students in India. J Educ Eval Health Prof. 2015;12:50. PMID:26582630. doi: 10.3352/jeehp.2015.12.50.
25. Nyangeni T, Du Rand S, Van Rooyen D. Perceptions of nursing students regarding responsible use of social media in the Eastern Cape. Curationis. 2015 Jul 23;38(2):1496. PMID: 26244464. doi: 10.4102/curationis.v38i2.1496.
26. Gettig JP, Noronha S, Graneto J, Obucina L, Christensen KJ, Fjortoft NF. Examining Health Care Students' Attitudes toward E-Professionalism. Am J Pharm Educ. 2016 Dec 25;80(10):169. PMID:28179718. doi: 10.5688/ajpe8010169.

27. Yang Y-M, Jeong E, Je NK, Jee J-P, Yoo JC, Choi EJ. An analysis of pharmacy students' social networking service activities and perceptions regarding e-professionalism under the newly implemented 6-year pharmacy educational system in South Korea. Indian Journal of Pharmaceutical Education and Research. 2016;50(1):63–9. doi: 10.5530/ijper.50.1.9

28. Chester AN, Walthert SE, Gallagher SJ, Anderson LC, Stitely ML. Patient-targeted Googling and social media: a cross-sectional study of senior medical students. BMC Med Ethics. 2017 Dec 4;18(1):70. PMID:29202840. doi: 10.1186/s12910-017-0230-9.

29. Knott PN, Wassif HS. Older and wiser? First year BDS graduate entry students and their views on using social media and professional practice. Br Dent J. 2018 Sep;225(5):437–40. PMID: 30168814. doi: 10.1038/sj.bdj.2018.745.

30. West CA, Wagner JM, Greenberg SB, Buck E, Hsieh P, Horn K, et al. Examining Medical Students' Social Media Beliefs and Behaviors and Their Relationship to Professional Identity. Medical Science Educator. 2018;28(2):389–99. doi: 10.1007/s40670-018-0562-1

31. Dobson E, Patel P, Neville P. Perceptions of e-professionalism among dental students: a UK dental school study. Br Dent J. 2019 Jan;226(1):73–8. PMID: 30631197**.** doi: 10.1038/sj.bdj.2019.11.

32. Hsieh J-G, Kuo L-C, Wang Y-W. Learning medical professionalism - the application of appreciative inquiry and social media. Med Educ Online. 2019 Dec;24(1):1586507. PMID:30831060. doi: 10.1080/10872981.2019.1586507.

33. Sadd R. Student Nurse Attitudes and Behaviours when Using Social Network Sites. Stud Health Technol Inform. 2019 Aug 21;264:1342–6. PMID:31438144. doi: 10.3233/SHTI190445.

34. Hinojo-Lucena F-J, Aznar-Díaz I, Cáceres-Reche M-P, Romero-Rodríguez J-M. Use of social networks for international collaboration among medical students. Educacion Médica. 2020 Mar 1;21(2):137–41. doi: 10.1016/j.edumed.2018.08.009

35. Karveleas I, Kyriakouli A, Koukou M, Koufatzidou M, Kalogirou E-M, Tosios KI. The relationship between Facebook behaviour and e-professionalism: A questionnaire-based cross-sectional study among Greek dental students. Eur J Dent Educ. 2020 Aug 11; PMID:32780448. doi: 10.1111/eje.12585.

36. Marnocha S, Marnocha MR, Pilliow T. Unprofessional content posted online among nursing students. Nurse Educ. 2015 May-Jun;40(3):119–23. PMID: 25501656. doi: 10.1097/NNE.0000000000000123.

37. Langenfeld SJ, Vargo DJ, Schenarts PJ. Balancing Privacy and Professionalism: A Survey of General Surgery Program Directors on Social Media and Surgical Education. J Surg Educ. 2016 Nov - Dec;73(6):e28–e32. PMID: 27524278. doi: 10.1016/j.jsurg.2016.07.010.

38. Henry RK, Pieren JA. The use of social media in dental hygiene programs: a survey of program directors. J Dent Hyg. 2014 Aug;88(4):243–9. PMID: 25134957

39. Al Qarni AM, Al Shehri SZ, Wahab MMA. Perceptions and Attitudes of Family Medicine Residency Program Directors Regarding Ranking Applicants for Residency Programs using their Social Media Accounts: A National Study in Saudi Arabia. J Fam Community Med. 2019 Aug;26(2):133–40. PMID:31143087. doi: 10.4103/jfcm.JFCM_176_18.

40. Staud SN, Kearney RC. Social Media Use Behaviors and State Dental Licensing Boards. J Dent Hyg. 2019 Jun;93(3):37–43. PMID:31182567
41. Jafarey A, Shekhani S, Mohsin-E-Azam, Gill R, Shirazi B, Hassan M, et al. Physicians in Cyberspace: Finding Boundaries. Asian Bioeth Rev. 2016 Dec;8(4):272–89. doi: 10.1353/asb.2016.0023.
42. Duke VJA, Anstey A, Carter S, Gosse N, Hutchens KM, Marsh JA. Social media in nurse education: Utilization and E-professionalism. Nurse Educ Today. 2017 Oct;57:8–13. PMID: 28683342. doi: 10.1016/j.nedt.2017.06.009.
43. Garg M, Pearson DA, Bond MC, Runyon M, Pillow MT, Hopson L, et al. Survey of Individual and Institutional Risk Associated with the Use of Social Media. West J Emerg Med. 2016 May;17(3):344–9. PMID: 27330669. doi: 10.5811/westjem.2016.2.28451.
44. Soares W, Shenvi C, Waller N, Johnson R, Hodgson CS. Perceptions of Unprofessional Social Media Behavior Among Emergency Medicine Physicians. J Grad Med Educ. 2017 Feb;9(1):85–9. PMID: 28261400. doi: 10.4300/JGME-D-16-00203.1.
45. Kitsis EA, Milan FB, Cohen HW, Myers D, Herron P, McEvoy M, et al. Who’s misbehaving? Perceptions of unprofessional social media use by medical students and faculty. BMC Med Educ. 2016 Feb 18;16:67. PMID: 26887561. doi: 10.1186/s12909-016-0572-x.
46. Brisson GE, Fisher MJ, LaBelle MW, Kozmic SE. Defining a mismatch: differences in usage of social networking sites between medical students and the faculty who teach them. Teach Learn Med. 2015;27(2):208–14. PMID: 25893945. doi: 10.1080/10401334.2015.1011648.
47. Pereira I, Cunningham AM, Moreau K, Sherbino J, Jalali A. Thou shalt not tweet unprofessionally: an appreciative inquiry into the professional use of social media. Postgrad Med J. 2015 Oct;91(1080):561–4. PMID: 26294333. doi: 10.1136/postgradmedj-2015-133353.
48. D’Souza K, Henningham L, Zou R, Huang J, O’Sullivan E, Last J, et al. Attitudes of Health Professional Educators Toward the Use of Social Media as a Teaching Tool: Global Cross-Sectional Study. JMIR Med Educ. 2017 Aug 4;3(2):e13. PMID: 28778841. doi: 10.2196/mededu.6429.
49. Kesselheim JC, Schwartz A, Belmonte F, Boland KA, Poynter S, Batra M, et al. A National Survey of Pediatric Residents’ Professionalism and Social Networking: Implications for Curriculum Development. Acad Pediatr. 2016 Mar;16(2):110–4. PMID: 26718877. doi:10.1016/j.acap.2015.12.004.
50. Langenfeld SJ, Cook G, Sudbeck C, Luers T, Schenarts PJ. An assessment of unprofessional behavior among surgical residents on Facebook: a warning of the dangers of social media. J Surg Educ. 2014 Dec;71(6):e28-32. PMID: 24981657. doi: 10.1016/j.jsurg.2014.05.013.
51. Scragg B, Shaikh S, Shires G, Stein Hodgins J, Mercer C, Robinson L, et al. An exploration of mammographers’ attitudes towards the use of social media for providing breast screening information to clients. Radiography (Lond). 2017 Aug;23(3):249–55. PMID: 28687294. doi: 10.1016/j.radi.2017.04.004.
52. Laliberté M, Beaulieu-Poulin C, Campeau Larrivée A, Charbonneau M, Samson É, Ehrmann Feldman D. Current Uses (and Potential Misuses) of Facebook: An Online Survey in Physiotherapy. Physiother Can. 2016;68(1):5–12. PMID: 27504042. doi: 10.3138/ptc.2014-41.
53. Fuoco M, Leveridge MJ. Early adopters or laggards? Attitudes toward and use of social media among urologists: Urologists’ use of social media. BJU Int. 2015 Mar;115(3):491–7. PMID: 24981237. doi: 10.1111/bju.12855.

54. Irfan KS, Farhana I, Eiad AF, Nassr AM, Al Mohammed AQ, Maya N, et al. Family physicians' utility of social media: a survey comparison among family medicine residents and physicians. Afr Health Sci. 2018 Sep;18(3):817-27. PMID: 30603016. doi: 10.4314/ahs.v18i3.41.

55. Panahi S, Watson J, Partridge H. Fostering interpersonal trust on social media: physicians' perspectives and experiences. Postgrad Med J. 2016 Feb;92(1084):70–3. PMID: 26627975. doi:10.1136/postgradmedj-2015-133270

56. Khandelwal A, Nugus P, Elkoushy MA, Cruess RL, Cruess SR, Smilovitch M, et al. How we made professionalism relevant to twenty-first century residents. Med Teach. 2015;37(6):538–42. PMID: 25594336. doi: 10.3109/0142159X.2014.990878.

57. Koo K, Bowman MS, Ficko Z, Gormley EA. Older and wiser? Changes in unprofessional content on urologists' social media after transition from residency to practice. BJU Int. 2018 Aug;122(2):337–43. PMID: 29694713. doi: 10.1111/bju.14363.

58. Dawkins R, King WD, Boateng B, Nichols M, Desselle BC. Pediatric Residents' Perceptions of Potential Professionalism Violations on Social Media: A US National Survey. JMIR Med Educ. 2017 Jan 31;3(1):e2. PMID: 28143804. doi: 10.2196/mededu.5993.

59. Benetoli A, Chen TF, Schaefer M, Chaar B, Aslani P. Pharmacists' perceptions of professionalism on social networking sites. Res Social Adm Pharm. 2017 May-Jun;13(3):575–88. PMID: 27423783. doi: 10.1016/j.sapharm.2016.05.044.

60. Levati S. Professional conduct among registered nurses in the use of online social networking sites. J Adv Nurs. 2014 Oct;70(10):2284–92. PMID: 24617801. doi: 10.1111/jan.12377.

61. Patel SS, Hawkins CM, Rawson JV, Hoang JK. Professional Social Networking in Radiology: Who Is There and What Are They Doing? Acad Radiol. 2017 May;24(5):574–9. PMID: 28153576. doi: 10.1016/j.acra.2016.09.026.

62. Wagner JP, Cochran AL, Jones C, Gusani NJ, Varghese TK Jr, Attai DJ. Professional Use of Social Media Among Surgeons: Results of a Multi-Institutional Study. J Surg Educ. 2018 May-Jun;75(3):804–10. PMID: 28964746. doi: 10.1016/j.jsurg.2017.09.008.

63. Adilman R, Rajmohan Y, Brooks E, Urgoiti GR, Chung C, Hammad N, et al. Social Media Use Among Physicians and Trainees: Results of a National Medical Oncology Physician Survey. J Oncol Pract. 2016 Jan;12(1):79–80, e52-60. PMID: 26443837. doi: 10.1200/JOP.2015.006429.

64. Neville P. Social media and professionalism: a retrospective content analysis of Fitness to Practise cases heard by the GDC concerning social media complaints. Br Dent J. 2017 Sep 8;223(5):353–7. PMID: 28883584. doi: 10.1038/sj.bdj.2017.765.

65. Klee D, Covey C, Zhong L. Social media beliefs and usage among family medicine residents and practicing family physicians. Fam Med. 2015 Mar;47(3):222-6. PMID: 25853534

66. Mohiuddin Z, Shahid H, Shuaib W. Social Media Impact: Utility of Reflective Approach in the Practice of Surgery. Indian J Surg. 2015 Dec;77(Suppl 3):893–8. PMID: 27011477. doi: 10.1007/s12262-014-1056-z.

67. Lefebvre C, Mesner J, Stopyra J, O'Neill J, Husain I, Geer C, et al. Social Media in Professional Medicine: New Resident Perceptions and Practices. J Med Internet Res. 2016 Jun 9;18(6):e119. PMID: 27283846. doi: 10.2196/jmir.5612.

68. Surani Z, Hirani R, Elias A, Quisenberry L, Varon J, Surani S, et al. Social media usage among health care providers. BMC Res Notes. 2017 Nov 29;10(1):654. PMID: 29187244. doi: 10.1186/s13104-017-2993-y.

69. Campbell L, Evans Y, Pumper M, Moreno MA. Social media use by physicians: a qualitative study of the new frontier of medicine. BMC Med Inform Decis Mak. 2016 Jul 15;16:91. PMID: 27418201. doi: 10.1186/s12911-016-0327-y.

70. Alshakhs F, Alanzi T. The evolving role of social media in health-care delivery: measuring the perception of health-care professionals in Eastern Saudi Arabia. J Multidiscip Healthc. 2018 Sep 21;11:473-79. PMID: 30275699. doi: 10.2147/JMDH.S171538.

71. Langenfeld SJ, Sudbeck C, Luers T, Adamson P, Cook G, Schenarts PJ. The Glass Houses of Attending Surgeons: An Assessment of Unprofessional Behavior on Facebook Among Practicing Surgeons. J Surg Educ. 2015 Nov-Dec;72(6):e280-5. PMID: 26276300. doi: 10.1016/j.jsurg.2015.07.007.

72. Weijs C, Majowicz S, Coe JB, Desmarais S, Jones-Bitton A. The personal use of Facebook by public health professionals in Canada: Implications for public health practice. J Commun Healthc. 2017 Jan 2;10(1):8–15. doi: 10.1080/17538068.2016.1274846.

73. Koo K, Ficko Z, Gormley EA. Unprofessional content on Facebook accounts of US urology residency graduates. BJU Int. 2017 Jun;119(6):955–60. PMID: 28393475. doi: 10.1111/bju.13846.

74. Borgmann H, DeWitt S, Tsaur I, Haferkamp A, Loeb S. Novel survey disseminated through Twitter supports its utility for networking, disseminating research, advocacy, clinical practice and other professional goals. Can Urol Assoc J. 2015 Oct;9(9–10):E713-7. PMID:26664662. doi: 10.5489/cuaj.3014.

75. Robertson M, Shoss MK, Broom MA. Social Media: Social Intelligence Training Module. MedEdPORTAL. 2016 Aug 26;12:10442. PMID:31008220. doi: 10.15766/mep_2374-8265.10442.

76. Duymuş TM, Karadeniz H, Şükür E, Atiç R, Zehir S, Azboy İ. Social media and Internet usage of orthopaedic surgeons. J Clin Orthop Trauma. 2017;8(1):25–30. PMID:28360492. doi: 10.1016/j.jcot.2016.10.007.

77. Long X, Qi L, Ou Z, Zu X, Cao Z, Zeng X, Li Y, Chen M, Wang Z, Wang L. Evolving use of social media among Chinese urologists: Opportunity or challenge? PLOS One. 2017 Jul 28;12(7):e0181895. PMID: 28753632. doi: 10.1371/journal.pone.0181895.

78. Nikiphorou E, Studenic P, Ammitzbøll CG, Canavan M, Jani M, Ospelt C, Berenbaum F, EMEUNET. Social media use among young rheumatologists and basic scientists: results of an international survey by the Emerging EULAR Network (EMEUNET). Ann Rheum Dis. 2017 Apr;76(4):712–5. PMID:27797750. doi: 10.1136/annrheumdis-2016-209718.

79. Hazzam J, Lahrech A. Health Care Professionals' Social Media Behavior and the Underlying Factors of Social Media Adoption and Use: Quantitative Study. J Med Internet Res. 2018 Nov 7;20(11):e12035. PMID:30404773. doi: 10.2196/12035.

80. Justinia T, Alyami A, Al-Qahtani S, Bashanfar M, El-Khatib M, Yahya A, Zagzoog F. Social Media and the Orthopaedic Surgeon: a Mixed Methods Study. Acta Inform Med. 2019 Mar;27(1):23–8. PMID:31213739. doi: 10.5455/aim.2019.27.23-28.

81. Lee YJ, Hwang J, Lee SI, Woo S-I, Hahn SW, Koh S. Impact of experience of psychiatrists and psychiatry residents regarding electronic communication and social networking on internet use patterns: a questionnaire survey for developing e-professionalism in South Korea. BMC Med Educ. 2019 Nov 8;19(1):411. PMID:31703677. doi: 10.1186/s12909-019-1771-z.

82. Renew JR, Ladlie B, Gorlin A, Long T. The Impact of Social Media on Anesthesia Resident Recruitment. J Educ Perioper Med. 2019 Mar;21(1):E632. PMID:31406704

83. Wang Z, Wang S, Zhang Y, Jiang X. Social media usage and online professionalism among registered nurses: A cross-sectional survey. Int J Nurs Stud. 2019 Oct;98:19–26. PMID:31255853. doi: 10.1016/j.ijnurstu.2019.06.001.

84. Ahmad T, Sattar K, Akram A. Medical professionalism videos on YouTube: Content exploration and appraisal of user engagement. Saudi J Biol Sci. 2020 Sep;27(9):2287–92. PMID:32884409. doi: 10.1016/j.sjbs.2020.06.007.

85. Kerr H, Booth R, Jackson K. Exploring the Characteristics and Behaviors of Nurses Who Have Attained Microcelebrity Status on Instagram: Content Analysis. J Med Internet Res. 2020 May 26;22(5):e16540. PMID:32452809. doi: 10.2196/16540.

86. Loo ME, Wong B, Lee YM. Evaluating the appropriateness of facebook posts – what do faculty and residents consider? The Asia Pacific Scholar. 2020;5(3):71–82. doi: 10.29060/TAPS.2020-5-3/OA2226

87. Low J, Tan M, Joseph R. Doctors and social media: knowledge gaps and unsafe practices. Singapore Med J. 2020 Apr 21. PMID: 32312027. doi: 10.11622/smedj.2020067.

88. Ruan B, Yilmaz Y, Lu D, Lee M, Chan TM. Defining the Digital Self: A Qualitative Study to Explore the Digital Component of Professional Identity in the Health Professions. J Med Internet Res. 2020 Sep 29;22(9):e21416. PMID: 32990636. doi: 10.2196/21416.
