# Supplementary material for: Dangers and Benefits of Social Media on E-Professionalism of Health Care Professionals: Scoping Review
Source: J Med Internet Res. 2021 Nov 17;23(11):e25770. doi: 10.2196/25770 (PMC8663533; doi:10.2196/25770)
Supplement: Multimedia Appendix 3 [file jmir_v23i11e25770_app3.docx]

**Multimedia Appendix 3.** List of the excluded studies and the reasons for exclusion.

**List of the excluded studies and the reasons for exclusion (n=671)**

**Excluded articles (n= 623)**

1. Were not in English (n=1)

- [1]

1. Were books, dissertations, reviews, reports, abstracts only, case studies, opinions, letters, commentaries, policies, guidelines or recommendations (n=420)

- [2-421]

1. Did not focused primarily on use of social media among health professionals (n=202)

- [422-623]

**Full-text articles excluded, with reasons (n = 48)**

1. Did not study the uses, benefits, or dangers of social media among health professionals (n=17)

- [624-640]

(5) Did not study HCPs as a study population (n=4)

- [641-644]

(6) Described the use of SMs primarily with a marketing or advertising focus (n=4)

- [645-648]

(7) Were not available as full text in the final search (n=23)

- [649-671]

**Full List of Excluded Articles (n=671)**

1. George RB, Lozada MJ. Anesthesiologists, it’s time to get social! Can J Anesth. 2017;64(12):1169–75. PMID: 28936589. doi: 10.1007/s12630-017-0976-z.
2. Alison Little. #NoFilter: Professionalism in social media. Emerg Med Australas. 2018;30(3):414–5. PMID: 29736932
3. O’Regan A, Smithson WH, Spain E. Social media and professional identity: Pitfalls and potential. Med Teach. 2018 Feb;40(2):112–6. PMID: 29172814. doi: 10.1080/0142159X.2017.1396308
4. ‘Talk to New Recruits About Their Use of Social Media.’ Nurs Stand. 2015 May 29;29(39):9–9. PMID: 26015104. doi: 10.7748/ns.29.39.9.s8
5. Shepherd J. 4. Promoting professional behaviour in practice. Practising Midwife. 2017;20(2):13–5.
6. Langenfeld J, Hall C J., Tran A, Sison-Martinez J, Finke D, Buzalko R, et al. 38 Social Media and Medical Professionalism: An Assessment of Behavior on Facebook by Emergency Medicine Residents. Annals of Emergency Medicine. 2016 Oct 2;68:S17–S17.
7. Chilvers A. A new era of digital professionalism. Journal of School Health (2053-1028). 2015 Mar;1(3):13–5.
8. A Nurse’s Guide to the Use of Social Media. Missouri State Board of Nursing Newsletter. 2017 Aug;19(3):3–5.
9. Weiner J. A personal reflection on social media in medicine: I stand, no wiser than before. Int Rev Psychiatry. 2015 Apr;27(2):155–60. PMID: 25847332 doi: 10.3109/09540261.2015.1015503
10. Peters ME, Uible E, Chisolm MS. A Twitter Education: Why Psychiatrists Should Tweet. Curr Psychiatry Rep. 2015 Dec;17(12):94. PMID: 26463050 doi:10.1007/s11920-015-0635-4
11. ACA Public Policy on Social Media. Journal (Michigan Association of Chiropractors). 2015 Aug 7;10–1.
12. Gallegos K. Academies unite to make an impact in patient care: Together we can. J Am Pharm Assoc. 2017;57(2):142–5. PMID:28285772 doi: 10.1016/j.japh.2017.02.001
13. Davis WM, Ho K, Last J. Advancing social media in medical education. CMAJ. 2015;187(8):549–50. PMID: 25852033 doi:10.1503/cmaj.141417
14. Foster S. Airing your opinions. Br J Nurs. 2016 May 12;25(9):527. PMID: 27172501 doi: 10.12968/bjon.2016.25.9.527
15. De Gagne JC, Choi M, Ledbetter L, Kang HS, Clark CM. An Integrative Review of Cybercivility in Health Professions Education. Nurse Edu. 2016 Sep-Oct 9;41(5):239–45. PMID: 27022683 doi: 10.1097/NNE.0000000000000264
16. Kiran S, Sethi N. Anaesthesiologist and social media: Walking the fine line. Indian J Anaesth. 2018 Oct;62(10):743–6. PMID: 30443055 doi: 10.4103/ija.IJA_449_18
17. DeNicola N, Good M, Newton L. Back to the future: A history of ACOG in social media’s golden age. Currt Opin Obstet Gynecol. 2014;26(6):495–502. PMID: 25377439 doi: 10.1097/GCO.0000000000000123
18. Wareing M. Becoming a health and social care professional. British Journal of Healthcare Assistants. 2017 Jan;11(1):32–9.
19. Being professional on social media. Dental Abstracts. 2017 Dec 11;62(6):325–7.
20. Cook CE, O’connell NE, Hall T, George SZ, Jull G, Wright AA, et al. Benefits and Threats to Using Social Media for Presenting and Implementing Evidence. J Orthop Sports Phys Ther. 2018 Jan;48(1):3–7. PMID: 29291281 doi: 10.2519/jospt.2018.0601
21. Logghe HJ, Boeck MA, Gusani NJ, Hardaway JC, Hughes KA, Mouawad NJ, et al. Best Practices for Surgeons’ Social Media Use: Statement of the Resident and Associate Society of the American College of Surgeons. J Am Coll Surg. 2018;226(3):317–27. PMID: 29224795 doi: 10.1016/j.jamcollsurg.2017.11.022
22. Sandlin JK, Hinmon D. Beyond Baby Steps. J Perinat Neonatal Nurs. 2016 Jul;30(3):204–8. PMID: 27465450 doi: 10.1097/JPN.0000000000000189
23. Peek HS, Richards M, Muir O, Chan SR, Caton M, MacMillan C. Blogging and Social Media for Mental Health Education and Advocacy: a Review for Psychiatrists. Curr Psychiatry Rep. 2015 Nov;17(11):88. PMID: 26377948 doi: 10.1007/s11920-015-0629-2
24. Cork N, Grant P. Blurred lines: the General Medical Council guidance on doctors and social media. Clin Med (Lond). 2016 Jun;16(3):219–22. PMID: 27251909 doi: 10.7861/clinmedicine.16-3-219
25. Allen L. Body Shaming on Social Media. Massage & Bodywork. 2017 Oct 9;32(5):35–35.
26. Hutchins SD. Building a Professional Network and Influencing People--Online. ASHA Leader. 2017 Dec;22(12):28–9.
27. Dehn RW. Can online PA programs reduce the cost of PA education? JAAPA. 2015 Jun;28(6):25–6. PMID: 25932712 doi: 10.1097/01.JAA.0000465228.28890.60
28. Gutierrez PL, Johnson DJ. Can Plastic Surgeons Maintain Professionalism within Social Media? AMA J Ethics. 2018 Apr 1;20(4):379–83. PMID: 29671732 doi: 10.1001/journalofethics.2018.20.4.msoc3-1804
29. Makdissi R. Capsule Commentary on Chretien et al., A Digital Ethnography of Medical Students who use Twitter for Professional Development. J Gen Intern Med. 2015 Nov;30(11):1701. PMID: 26227156 doi: 10.1007/s11606-015-3373-8
30. Gonsalves C. Capsule commentary on Rocha et al., opinions of students from a Brazilian medical school on online professionalism. J Gen Intern Med. 2014;29(5):787. PMID: 24519103 doi: 10.1007/s11606-014-2796-y
31. Bayne CE, Davies BJ. Chipping away at the body politic one study at a time: the case for more ‘unprofessional’ online content. BJU Int. 2017;120(5):609–10. PMID: 28805335 doi: 10.1111/bju.13986
32. Neville P. Clicking on professionalism? Thoughts on teaching students about social media and its impact on dental professionalism. Eur J Dent Educ. 2016 Feb;20(1):55–8. PMID: 25704609 doi: 10.1111/eje.12142
33. Chretien KC, Kind T. Climbing social media in medicine’s hierarchy of needs. Acad Med. 2014;89(10):1318–20. PMID: 25076202 doi: 10.1097/ACM.0000000000000430
34. Clinical digest. Most nurses are responsible Facebook users but a minority share compromising material. Nursing Standard. 2014 Nov 5;29(10):17–17.
35. Manning L. Code of Conduct refresher. Nursing Review (1173-8014). 2017 Nov 10;17(5):15–8.
36. Al-Amodi S, Mathew S, Fox J, Sabit R, Patricolo A. Collaborate online as a small group. Educ Prim Care. 2015;26(2):127–9. PMID: 25898306
37. Udovicich C, Kasivisvanathan V, Winchester CL. Communicating your research (part 2): to the wider community. Journal of Clinical Urology. 2018;11(3):208–14.
38. Krouse JH. Congratulations 2016. Otolaryngology - Head and Neck Surgery (United States). 2016;154(1):3–4.
39. Chinn T. Connecting and celebrating: social media for gastrointestinal nurses. Gastrointestinal Nursing. 2017 Oct;15(8):14–9.
40. Ménage D. Connecting for compassion. Practising Midwife. 2015 Mar;18(3):32–5. PMID: 26349330
41. Kraakevik J. Crafting a positive professional digital profile to augment your practice. Neurol Clin Pract. 2016;6(1):87–93. PMID: 29443275 doi: 10.1212/CPJ.0000000000000211
42. Walega D. Deliberate Malfeasance or Innocent Error? Misrepresentation in Pain Medicine Fellowship Applications. Pain Medicine (United States). 2015;16(2):230–1. PMID: 25580778 doi: 10.1111/pme.12673
43. Cornock M. Digital diagnosis. Nursing Standard. 2017 Oct 11;32(7):28–28.
44. Lemire F. Digital engagement. Can Fam Physician. 2018;64(4):319–20. PMID: 29650615
45. Gyke P. Digital professionalism. Ohio nurses review. 2014;89(3):S4. PMID: 25007557
46. Mishra B. Doctors at cross road. Indian Heart Journal. 2015;67(5):425–6. PMID: 26432727
47. Sokol D. Doctors shouldn’t reveal so much. BMJ (Online). 2018 Jun;361:k2495. PMID: 29891586
48. Brooks S. Does personal social media usage affect efficiency and well-being? Computers in Human Behavior. 2015 May;46:26–37.
49. Safdar A. Don’t be scared to use social media professionally. Clinical Pharmacist. 2015;7(2).
50. Twist MLC, Hertlein KM. E-mail Me, Tweet Me, Follow Me, Friend Me: Online Professional Networking Between Family Therapists. Journal of Feminist Family Therapy. 2015 Jul;27(3/4):116–33.
51. e-Professionalism. Dental Abstracts. 2016 Feb 1;61(1):13–5.
52. McAuliffe D, Nipperess S. e-Professionalism and the Ethical Use of Technology in Social Work. Australian Social Work. 2017 Apr 3;70(2):131–4.
53. Madanick RD. Education Becomes Social: The Intersection of Social Media and Medical Education. Gastroenterology. 2015;149(4):844–7. PMID: 26311278 doi: 10.1053/j.gastro.2015.08.037
54. Scruth EA, Pugh DM, Adams CL, Foss-Durant AM. Electronic and Social Media: The Legal and Ethical Issues for Healthcare. Clin Nurse Spec. 2015 Feb 1;29(1):8–11. PMID: 25469434 doi: 10.1097/NUR.0000000000000089
55. Murphy DG, Loeb S, Basto MY, Challacombe B, Trinh Q-D, Leveridge M, et al. Engaging responsibly with social media: The British Journal of Urology International (BJUI) guidelines. BJU International. 2014;114(1):9–11.
56. Kubheka B. Ethical and legal perspectives on the medical practitioners use of social media. S Afr Med J. 2017 Apr 25;107(5):386–9. PMID: 28492116 doi: 10.7196/SAMJ.2017.v107i5.12047
57. Rouprêt M, Morgan TM, Bostrom PJ, Cooperberg MR, Kutikov A, Linton KD, et al. European Association of Urology (@Uroweb) recommendations on the appropriate use of social media. Eur Urol. 2014;66(4):628–32. PMID: 25043941 doi: 10.1016/j.eururo.2014.06.046
58. Ellaway RH, Coral J, Topps D, Topps M. Exploring digital professionalism. Med Teach. 2015;37(9):844–9. PMID: 26030375 doi: 10.3109/0142159X.2015.1044956
59. Parks M, Sorby K, Mcfarland DB, Wallbank J, King HR. Adams, D. Exploring the use of social media by students on an occupational therapy programme. PosterP64 [Abstract from RCOT (Royal College of Occupational Therapist) Annual Conference 2017]. British Journal of Occupational Therapy. 2017 Aug 2;80:93–93.
60. Gatter P. Facebook alert. Therapy Today. 2016 Feb;27(1):40–40.
61. Kocemba P, Lasota M, Sroka NH, Feleszko W. Facebook-based medicine, or the doctor’s professional image on the Internet. Pediatria i Medycyna Rodzinna. 2015;11(3):328–38.
62. Dillon N. Facebook, My Space, Blog, or Tweet, What You Say May Not Be Sweet: Professional Boundaries and Social Media...APNA 31st Annual Conference, October 18-21, 2017, Phoenix, Arizona: Part I. Journal of the American Psychiatric Nurses Association. 2018 Jun 5;24(3):278–9.
63. Ho A, Hee N, Teo R. Facilitating co‐learning of medical ethical issues using Facebook. Med Educ. 2018 May;52(5):560–1. PMID: 29672945 doi: 10.1111/medu.13577
64. Skrabal J. Factors and Processes That Influence E-Professionalism among Pre-Licensure Baccalaureate Nursing Students When Utilizing Social Media. Factors & Processes That Influence E-Professionalism among Pre-Licensure Baccalaureate Nursing Students When Utilizing Social Media. 2017 Jan;(Ed.D.):1–1.
65. Caplan AL, Teagarden JR, Kearns L, Bateman-House AS, Mitchell E, Arawi T, et al. Fair, just and compassionate: A pilot for making allocation decisions for patients requesting experimental drugs outside of clinical trials. J Med Ethics. 2018 Nov;44(11):761–7. PMID: 29982174
66. Fleet B. Features. Facing up to Facebook: a student’s perspective. Midwifery News. 2015 Jun;(77):30–30.
67. Kodadek LM. First-place essay--Con: The writing is on the (Facebook) wall: The threat posed by social media. Bull Am Coll Surg. 2015 Nov;100(11):21–3. PMID: 2667753
68. Scott Johnson R, Chiu LL, Czelusta K-L. For residents, technology can put professionalism and reputation at risk. Current Psychiatry. 2015;14(7):e3–4.
69. Budd L, Fidler L, Anand A. Gaining competence through social media. CMAJ. 2016;188(13):E311–2. PMID: 27402073 doi: 10.1503/cmaj.160255
70. Bastian D. Get a Cert III in Snapchat...Carey Mather. Nursing Review (1326-0472). 2016 Oct 9;(5):32–32.
71. Mather CA, Gale F, Cummings EA. Governing mobile technology use for continuing professional development in the Australian nursing profession. BMC Nurs. 2017;16:17. PMID: 28428731
72. Walji M, Stanbrook MB. Health professionalism must be ensured online and offline. CMAJ. 2015 May 19;187(8):547. PMID: 25852035
73. Wake C. Hey presto! Dental Nursing. 2018 Mar;14(3):126–7.
74. Logghe HJ, McFadden CL, Tully NJ, Jones C. History of Social Media in Surgery. Clin Colon Rectal Surg. 2017 Sep;30(4):233–9. PMID: 28924395
75. Langenfeld SJ, Batra R. How Can Social Media Get Us in Trouble? Clin Colon Rectal Surg. 2017 Sep;30(4):264–9. PMID: 28924400 doi: 10.1055/s-0037-1604255
76. Pound P. How should mandatory sex education be taught? BMJ. 2017 11;357:j1768. PMID: 28400378
77. Singleton N. How to Separate Personal and Office Life. American Chiropractor. 2016 Jan;38(1):26–7.
78. Chudleigh M, Jones R. How to set up and use a Twitter account professionally. Nurs Stand. 2016 Nov 2;31(10):40–3. PMID: 27861058
79. Ashurst A. How to...manage staffs’ use of social media at work. Nursing & Residential Care. 2015 Jul;17(7):416–416.
80. Hartley M. I posted a photo of my granddaughter, forgetting that dental hygienists were watching. RDH. 2018 May;38(5):8–8.
81. Sinclair M. I spy with my little electronic eye your digital profile: living with the discoverability factor. Evidence Based Midwifery. 2018 Mar;16(1):3–3.
82. Staton-Williams D. In Your Corner — Friend Requests From Patients. Tar Heel Nurse. 2015 Oct 15;77(6):20. PMID: 26721170
83. Hopkins EE, Spadaro KC, Walter L, Wasco JJ, Fisher M, Sterrett SE. Incivility in the Online Classroom: A Guide for Policy Development. Nurs Forum. 2017 Oct;52(4):306–12. PMID: 28419515
84. Delgado-López PD, Corrales-García EM. Influence of Internet and Social Media in the Promotion of Alternative Oncology, Cancer Quackery, and the Predatory Publishing Phenomenon. Cureus. 2018 May 13;10(5):e2617. PMID: 30027009
85. Scott KR, Hsu CH, Johnson NJ, Mamtani M, Conlon LW, DeRoos FJ. Integration of social media in emergency medicine residency curriculum. Ann Emerg Med. 2014;64(4):396–404. PMID: 24957931
86. Power A. Is Facebook an appropriate platform for professional discourse? British Journal of Midwifery. 2015 Feb 2;23(2):140–2.
87. Ireland B. Is your address hotchick@gmail.com? Dental Nursing. 2017 Apr;13(4):169–70.
88. Brous E, Olsen DP. Lessons Learned from Litigation: Legal and Ethical Consequences of Social Media. Am J Nurs. 2017 Sep;117(9):50–4. PMID: 28837489
89. Jones-Berry S. Let Twitter and Facebook improve rather than ruin your practice. Nurs Stand. 2016 Feb 10;30(24):12–3. PMID: 26860144
90. Orkin A, Ovens H, McLeod S, Varner C, Melady D, Thompson C, et al. Letter in Response to: “CJEM Debate Series: #Social Media – Social Media has Created Emergency Medicine Celebrities Who Now Influence Practice More Than Published Evidence.” CJEM. 2018 Oct;20(S2):S89–S89.
91. Schroeder WK. Leveraging Social Media in #FamilyNursing Practice. J Fam Nurs. 2017 Feb;23(1):55–72. PMID: 28795931
92. Kalia V, Ortiz DA, Patel AK, Moriarity AK, Canon CL, Duszak R. Leveraging Twitter to Maximize the Radiology Meeting Experience. J Am Coll Radiol. 2018;15(1 Pt B):177–83. PMID: 29162419
93. Power A. LinkedIn: Facebook for professionals? British Journal of Midwifery. 2015 Mar;23(3):196–8.
94. Savage A. Linkedln or out? Can social media platforms be useful to POGP members? Journal of Pelvic, Obstetric & Gynaecological Physiotherapy. 2016 Sep;(119):6–14.
95. Baron A, Townsend R. Live tweeting by ambulance services: a growing concern. Journal of Paramedic Practice. 2017 Jul;9(7):282–6.
96. Conrad S. Manage Your Online Reputation. Physical Therapy. 2015 Sep 2;6–10.
97. Ford C. Managing Multiple Twitter Accounts. MLA News. 2016 Jan;56(1):6–6.
98. Matthews EP, Matthews TM. Medical Ethics and Law in Radiologic Technology. Radiol Technol. 2015 Dec 11;87(2):163–84. PMID: 26538219
99. Birrer RB, Tokuda Y. Medicalization: A historical perspective. J Gen Fam Med. 2017;18(2):48–51. PMID: 29263990
100. Shepherd J. Midwifery basics 4.Promoting professional behaviour in practice. Practising Midwife. 2017 Feb;20(2):1–6.
101. Humphrey C. Midwives Have Rights Too - Reputation and Cyberbullying. Midwifery News. 2015 Dec;(79):14–14.
102. Brugaletta S. Moving fast towards the future: The role of #SoMe in learning cardiology. EuroIntervention. 2018;13(16):1874–5. PMID: 29555618
103. Bates D. Music Therapy Ethics “2.0”: Preventing User Error in Technology. Music Therapy Perspectives. 2014 Dec;32(2):136–41.
104. Daviss S, Hanson A, Miller D. My three shrinks: Personal stories of social media exploration. Int Rev Psychiatry. 2015 Apr;27(2):167–73. PMID: 25906990
105. Citrome L. My two favourite professional social networking sites: LinkedIn and ResearchGate - How they can help you, or hurt you. Int J Clin Pract. 2015;69(6):623–4. PMID: 26010939
106. Baker MJ, George DR, Kauffman GL Jr. Navigating the Google Blind Spot: An Emerging Need for Professional Guidelines to Address Patient-Targeted Googling. J Gen Intern Med. 2015;30(1):6–7. PMID: 25227741
107. Navigating the World of Social Media. Nursing News. 2016 Jan;40(1):19–19.
108. Riley B. Need for standardized professionalism training in the osteopathic family practice residency training. Osteopathic Family Physician. 2014;6(3):42–4.
109. Moorley C, Watson R. NMC code advice on digital communications. Nurs Times. 2015 Apr 1-7;111(14):22–3. PMID: 26182587
110. Bhatti J. Nurses in a Social Media World. Arizona Nurse. 2016 Feb;69(1):11–11.
111. Green J. Nurses’ online behaviour: lessons for the nursing profession. Contemp Nurse. 2017 Jun;53(3):355–67. PMID: 28077035
112. Halcomb E, Stephens M, Bryce J, Foley E, Ashley C. Nursing competency standards in primary health care: an integrative review. J Clin Nurs. 2016 May;25(9–10):1193–205. PMID: 26990487
113. Hoilman JA. Nursing students’ perspectives on social media e-professionalism in undergraduate nursing programs. Nursing Students’ Perspectives on Social Media E-Professionalism in Undergraduate Nursing Programs. 2016 Jan;1–1.
114. Westrick SJ. Nursing Students’ Use of Electronic and Social Media: Law, Ethics, and E-Professionalism. Nurs Educ Perspect. 2016 Feb;37(1):16–22. PMID: 27164772
115. John B. Online professionalism. World of Irish Nursing & Midwifery. 2015 Sep;23(7):47–47.
116. Browne C, Cantelo J. Online professionalism - A student perspective. A response to Hemming et al. Henning M, Hawken S, MacDonald J,et al.,Exploring educational interventions to facilitate health professional students’ professionally safe online presence. Med Teach. 39:959–966. 2017. Med TeacH. 2018 Jan;40(1):107–8. PMID: 28920506
117. Borgmann H, Cooperberg M, Murphy D, Loeb S, N’Dow J, Ribal MJ, et al. Online Professionalism-2018 Update of European Association of Urology (@Uroweb) Recommendations on the Appropriate Use of Social Media. Eur Urol. 2018 Nov;74(5):644–50. PMID: 30177286
118. Chretien KC, Tuck MG. Online professionalism: A synthetic review. Int Rev Psychiatry. 2015 Apr;27(2):106–17. PMID: 25804627
119. Herron PD. Opportunities and ethical challenges for the practice of medicine in the digital era. Curr Rev Musculoskelet Med. 2015 Jun;8(2):113–7. PMID: 25786848
120. Holden ACL. Paradise Lost; the reputation of the dental profession and regulatory scope. Br Dent J. 2017 Feb 24;222(4):239–41. PMID: 28232715
121. Colbert JA, Lehmann LS. Partnering with patients to realize the benefits of social media. Am J Obstet Gynecol. 2015;212(3):302-3.e1. PMID: 25526874
122. Peedin AR, Karp JK. Pathology and Professionalism in the Age of Social Media. Arch Pathol Lab Med. 2018 Apr;142(4):441–2. PMID: 29251997
123. Kleppinger CA, Cain J. Personal Digital Branding as a Professional Asset in the Digital Age. Am J Pharm Educ. 2015 Aug 25;79(6):79. PMID: 26430266
124. Thoma B. Personal reflections on exploring social media in medicine. Int Rev Psychiatry. 2015 Apr;27(2):161–6. PMID: 25750994
125. Grindrod K, Forgione A, Tsuyuki RT, Gavura S, Giustini D. Pharmacy 2.0: A scoping review of social media use in pharmacy. Res Social Adm Pharm. 2014;10(1):256–70. PMID: 23810653
126. Peluchette JV, Karl KA, Coustasse A. Physicians, patients, and Facebook: Could you? Would you? Should you? Health Mark Q. 2016 Apr;33(2):112–26. PMID: 27295007
127. Kaldy J. Policy Remedies for Social Media Headaches. Caring for the Ages. 2015 Jun;16(6):11–11.
128. Helm J, Jones RM. Practice Paper of the Academy of Nutrition and Dietetics: Social Media and the Dietetics Practitioner: Opportunities, Challenges, and Best Practices. J Acad Nutr Diet. 2016;116(11):1825–35. PMID: 27788767
129. Clark J. President's massage. Getting to the “Us.” Audiology Today. 2018 Feb 1;30(1):8–8.
130. Johnstone M-J. Privacy, professionalism and social media. Aust Nurs Midwifery J. 2016 Feb;23(7):23.
131. Evatt M, Guttendorf J. Professional Boundaries on Social Media: Risks and Consequences. Med-Surg Matters. 2017 Apr 3;26(2):1–8.
132. Professional Boundaries: A Guide to the Importance of Appropriate Professional Boundaries. Oregon State Board of Nursing Sentinel. 2015 Dec;34(4):9–12.
133. Day-Calder M. Student life: Professional conduct. Nurs Stand. 2016 6/1/2016;30(40):66–66.
134. Sabin JE, Harland JC. Professional Ethics for Digital Age Psychiatry: Boundaries, Privacy, and Communication. Curr Psychiatry Rep. 2017 Sep;19(9):55. PMID: 28726059
135. Baron RJ. Professional self-regulation in a changing world: Old problems need new approaches. JAMA. 2015;313(18):1807–8. PMID: 25965218
136. Lai PBS. Professionalism and #hellomynameis. Surgical Practice. 2017;21(3):101–2.
137. Greaney D, Nolan G, Crowe S. Professionalism and social media in pediatric anesthesia. Paediatr Anaesth. 2017;27(8):866–7. PMID: 28685979
138. Gagnon K, Sabus C. Professionalism in a digital age: opportunities and considerations for using social media in health care. Phys Ther. 2015 Mar;95(3):406–14. PMID: 24903111
139. Yang H. Professionalism in anesthesia. Can J Anaesth. 2017 Feb;64(2):149–57. PMID: 27650648. doi: 10.1007/s12630-016-0738-3.
140. Güner G, Müderrisoğlu C, Kösemehmetoğlu K, Söylemezoğlu F. Professionalism in Pathology: The Turkish Experience. Arch Pathol Lab Med. 2018 Apr;142(4):433–4. PMID: 29565209 doi: 10.5858/arpa.2017-0452-LE
141. Domen RE, Johnson K, Conran RM, et al. Professionalism in pathology: a case-based approach as a potential educational tool. Arch Pathol Lab Med. 2017;141(2):215–219. PMID: 27763788
142. Hilton L. Professionalism in social media is a balancing act. Dermatology Times. 2016 Jul;37(7):60–60.
143. Samson K. Professionalism-Advocacy: AAN PALF Advocate: Neurologists Needed in Soccer Return-to-Play Decisions. Neurology Today. 2018 Jul 19;18(14):24–6.
144. Barnes HA. “Professor Siemms (and Son) Surgeon-Chiropodist and Manicure” -- Victorian Chiropodists Operating in Wales, and Other Interesting Characters in the History of Chiropody in the UK. Podiatry Now. 2017 Aug;20(8):22–6.
145. Torous J, Keshavan M, Gutheil T. Promise and perils of digital psychiatry. Asian J Psychiatr. 2014;10:120–2. PMID: 25042968 doi: 10.1016/j.ajp.2014.06.006
146. Shepherd J. Promoting professional behavior in practice. Pract Midwife. 2017 Feb;20(2):13–5. PMID: 30462426
147. Ashurst A. Promoting professionalism and trust in care home nurses. Nursing & Residential Care. 2017 Nov;19(11):654–5.
148. Hryhorczuk AL, Hanneman K, Eisenberg RL, Meyer EC, Brown SD. Radiologic Professionalism in Modern Health Care. Radiographics. 2015 Oct;35(6):1779–88. PMID: 26466185
149. Ñamendys-Silva SA. Reader comments. Baylor University Medical Center Proceedings. 2016 Jan;29(1):84–84.
150. Tarrant B. Real and relevant: using web 2.0 social media technology to deepen and generalise learning in the health sciences and bridge the theory-practice gap. Physical Therapy Reviews. 2018 Feb;23(1):61–7.
151. Barnhoorn PC, Domen RE. Resident remediation: Start from scratch: To the editor. Am J Clin Pathol. 2015;144(3):525. PMID: 26276784
152. Jones-Berry S. Revealed: the price nurses pay for ill-judged postings on social media. Nurs Stand. 2016 Feb 10;30(24):7. PMID: 26860133
153. Bhardwa S. Revolutionising care with the Code. Independent Nurse. 2015 Apr 6;39–41.
154. Attai DJ, Anderson PF, Fisch MJ, Graham DL, Katz MS, Kesselheim J, et al. Risks and benefits of Twitter use by hematologists/oncologists in the era of digital medicine. Semin Hematol. 2017;54(4):198–204. PMID: 29153081
155. Arif Z. Safe use of social media. Nurs Stand. 2016 Nov 30;31(14):28. PMID: 27902159
156. Freshwater E. Saying the wrong thing. Br J Nurs. 2015 May 14;24(9):497. PMID: 25978285
157. SCoR publishes new guidance on the use of social media. Synergy News. 2016 Jan;10–10.
158. Ibrahim AM. Seeing is Believing: Using Visual Abstracts to Disseminate Scientific Research. Am J Gastroenterol. 2018;113(4):459–61. PMID: 28925990
159. Sullivan ME, Frishman GN, Vrees RA. Showing your public face: does screening social media assess residency applicants’ professionalism? Am J Obstet Gynecol. 2017 Nov;217(5):619–20. PMID: 28784415
160. Smethurst C. Social media -- does the dental world need it? Dental Nursing. 2017 Feb;13(2):81–2.
161. Collier A. Social media -- personal versus professional life. Dental Nursing. 2017 Oct;13(10):494–5.
162. Dimov V, Gonzalez-Estrada A, Eidelman F. Social Media and Allergy. Curr Allergy Asthma Rep. 2018 Nov 14;18(12):76. PMID: 30430277
163. Neville P, Waylen A. Social media and dentistry: some reflections on e-professionalism. Br Dent J. 2015 Apr 24;218(8):475–8. PMID: 25908363.
164. Katz MS. Social media and medical professionalism: The need for guidance. Eur Urol. 2014;66(4):633–4. PMID: 25107636
165. Risling T. Social Media and Nurs Leadersh (Tor Ont): Unifying Professional Voice and Presence. Nurs Leadersh (Tor Ont). 2016;28(4):48–57. PMID: 27122090
166. Lewis MA, Dicker AP. Social Media and Oncology: The Past, Present, and Future of Electronic Communication Between Physician and Patient. Semin Oncol. 2015 Oct;42(5):764–71. PMID: 26433557
167. Ehlert MJ. Social Media and Online Communication: Clinical Urology Practice in the 21st Century. Urology Practice. 2015;2(1):2–6.
168. Henderson ML, Clayville KA, Fisher JS, Kuntz KK, Mysel H, Purnell TS, et al. Social media and organ donation: Ethically navigating the next frontier. Am J Transplant. 2017 Nov;17(11):2803–9. PMID: 28744966
169. Webber EC, Fu P Jr., McKenna MP. Social media and pediatric hospital medicine: How our discipline benefits from a virtual community and why it matters for our future. Hosp Pediatr. 2014;4(1):51–3. PMID: 24435603
170. Holden A. Social media and professionalism: does the profession need to re-think the parameters of professionalism within social media? Aust Dent J. 2017 Mar;62(1):23–9. PMID: 27468729
171. McLawhorn AS, De Martino I, Fehring KA, Sculco PK. Social media and your practice: navigating the surgeon-patient relationship. Curr Rev Musculoskelet Med. 2016;9(4):487–95. PMID: 27766538
172. Bibault J-E, Katz MS, Motwani S. Social media for radiation oncologists: A practical primer. Adv Radiat Oncol. 2017;2(3):277–80. PMID: 29114592
173. Pillow MT, Hopson L, Bond M, Cabrera D, Patterson L, Pearson D, et al. Social media guidelines and best practices: recommendations from the Council of Residency Directors Social Media Task Force. West J Emerg Med. 2014;15(1):26-30. PMID: 24578765 doi: 10.5811/westjem.2013.7.14945
174. Hillman T, Sherbino J. Social media in medical education: A new pedagogical paradigm? Postgrad Med J. 2015;91(1080):544–5. PMID: 26338982 doi: 10.1136/postgradmedj-2015-133686
175. Cobbett J, Tran V, Humphrey K. Social media in medical education: How far have we come? Emerg Med Australas. 2018 Jun;30(3):420–2. PMID: 29732689 doi: 10.1111/1742-6723.13102
176. Chisolm MS. Social media in medicine: The volume that Twitter built. Int Rev Psychiatry. 2015 Apr;27(2):83–4. PMID: 25828741 doi: 10.3109/09540261.2015.1026239
177. O’Connor S, Jolliffe S, Stanmore E, Renwick L, Booth R. Social media in nursing and midwifery education: A mixed study systematic review. J Adv Nurs. 2018 Oct;74(10):2273–89. PMID: 30019486 doi: 10.1111/jan.13799
178. Lonzer J, Lonzer D, Medina M, Piedimonte G. Social media in pediatrics: A call for guidelines. J Pediatr. 2015;166(3):511–2. PMID: 25722261 doi: 10.1016/j.jpeds.2014.11.032
179. Stott I. Social media in the workplace: approach with caution. Nursing & Residential Care. 2015 Sep;17(9):519–21.
180. McMurtrie H. Social Media now comes as standard. Midwifery News. 2014 Dec;(75):29–29.
181. Loeb S, Catto J, Kutikov A. Social media offers unprecedented opportunities for vibrant exchange of professional ideas across continents. Eur Urol. 2014;66(1):118–9. PMID: 24630683 doi: 10.1016/j.eururo.2014.02.048
182. Campbell S, Chong S, Ewen V, Toombs E, Tzalazidis R, Maranzan KA. Social Media Policy for Graduate Students: Challenges and Opportunities for Professional Psychology Training Programs. Canadian Psychology. 2016 Aug;57(3):202–10.
183. Hunter T. Social Media Posts and Professionalism. Dental Assistant. 2015 Jun 5;22–3.
184. Shah V, Kotsenas AL. Social Media Tips to Enhance Medical Education. Acad Radiol. 2017;24(6):747–52. PMID: 28222940 doi: 10.1016/j.acra.2016.12.023
185. Grajales FJ 3rd, Sheps S, Ho K, Novak-Lauscher H, Eysenbach G. Social media: a review and tutorial of applications in medicine and health care. J Med Internet Res. 2014;16(2):e13. PMID: 24518354 doi: 10.2196/jmir.2912
186. Morris K. Social Media: Implications for Nursing Nursing Practice Statement NP 85. Ohio Nurses Rev. 2015 Jun 5;90(3):29–31.
187. Powers-Kilburn A. Social Media: It’s All About the Conversation! Journal of Legal Nurse Consulting. 2016 Spring;27(1):20–3.
188. Merkel-Walsh R, Moore J. Social Media: It’s Different for Professionals. ASHA Leader. 2018 Jul;23(7):30–4.
189. Katarey D, Francis I. Social media: paternalism versus professionalism. Clin Teach. 2015 Apr;12(2):145. PMID: 25789910 doi: 10.1111/tct.12343
190. Roy D, Taylor J, Cheston CC, Flickinger TE, Chisolm MS. Social Media: Portrait of an Emerging Tool in Medical Education. Acad Psychiatry. 2016 Feb;40(1):136–40. PMID: 25800704 doi: 10.1007/s40596-014-0278-5
191. Affleck P. Social media: Professionalism. Br Dent J. 2017 27;222(2):68–9. PMID: 28127020 doi: 10.1038/sj.bdj.2017.55
192. Leichtle SW. Social media: Threat to professionalism and privacy or essential for current surgical practice? Bull Am Coll Surg. 2015 Nov;100(11):17. PMID: 26677530.
193. Pershad Y, Hangge PT, Albadawi H, Oklu R. Social Medicine: Twitter in Healthcare. J Clin Med. 2018 May 28;7(6). pii: E121. PMID: 29843360 doi: 10.3390/jcm7060121
194. Social networking guidance. Journal of Perioperative Practice. 2015 Aug 7;25(7/8):122–122.
195. Nevins M, Smith K. SoMe, SoWhat? #SoEssential: using social media as a key learning and teaching tool. Journal of Paramedic Practice. 2016 Aug;8(8):386–8.
196. Special Issue: eProfessionalism and the Ethical Use of Technology in Social Work. Australian Social Work. 2015 Jul;68(3):404–404.
197. Griffith L. Staying professional on social media...“Lessons Learned from Litigation: Legal and Ethical Consequences of Social Media” (Legal Clinic , September). Am J Nurs. 2017 Dec;117(12):13. PMID: 29189224 doi: 10.1097/01.NAJ.0000527467.08470.b7
198. Regenberg AC. Stem cell science should be tweeted. Regen Med. 2014;9(2):125–7. PMID: 24750051 doi: 10.2217/rme.13.94
199. Harmse B, Retief I. Striving for excellence: investigating the practical aspects of dietetic practice. South African Journal of Clinical Nutrition. 2015 Jun;28(2):89–91.
200. Oxtoby K. Student life - be sociable, but be careful. Nurs Stand. 2014;28(45):66. PMID: 25005423 doi: 10.7748/ns.28.45.66.s55
201. McCann B. Students & education. Professionals Online: Safeguarding DCs...part 2. ACA News (American Chiropractic Association). 2015 Oct;11(8):34–5.
202. Chappell H, Utrera D. Students, Professionalism and Social Media. Journal of the Academy of Nutrition & Dietetics. 2015 Sep 2;115:A13–A13.
203. Azoury SC, Bliss LA, Ward WH, Liepert AE, Leichtle SW. Surgeons and social media: Threat to professionalism or an essential part of contemporary surgical practice? Bull Am Coll Surg. 2015 Aug;100(8):45–51. PMID: 26419055
204. Jalali A, Wood TJ. Teaching medical students social media: must or bust. Med Educ. 2014 Nov;48(11):1128–9. PMID: 25307668 doi: 10.1111/medu.12585
205. Phillippi JC, Schorn MN, Moore-Davis T. The APGAR rubric for scoring online discussion boards. Nurse Educ Pract. 2015 May;15(3):239–42. PMID: 25735842 doi: 10.1016/j.nepr.2014.11.002
206. Ljungberg A, Denhov A, Topor A. The Art of Helpful Relationships with Professionals: A Meta-ethnography of the Perspective of Persons with Severe Mental Illness. Psychiatr Q. 2015 Dec;86(4):471–95. PMID: 25631156 doi: 10.1007/s11126-015-9347-5
207. Van Geertruyden PH. The AWOL Business Card. J Am Coll Radiol. 2018 Aug;15(8):1188–9. PMID: 30078436 doi: 10.1016/j.jacr.2018.03.047
208. Tilburt JC, Allyse M, Hafferty FW. The Case of Dr. Oz: Ethics, Evidence, and Does Professional Self-Regulation Work? AMA J Ethics. 2017 Feb 1;19(2):199–206. PMID: 28225701 doi: 10.1001/journalofethics.2017.19.2.msoc1-1702
209. Gibbs T. The changing face of professionalism: Reflections in a cracked mirror. Med Teach. 2015;37(9):797–8. PMID: 26082969 doi: 10.3109/0142159X.2015.1054797
210. Egener BE, Mason DJ, McDonald WJ, Okun S, Gaines ME, Fleming DA, et al. The Charter on Professionalism for Health Care Organizations. Acad Med. 2017 Aug;92(8):1091–9. PMID: 28079726 doi: 10.1097/ACM.0000000000001561
211. The code. Midwives. 2015 Spring;18(1):70–1.
212. Preminger BA, Hansen J, Reid CM, Gosman AA. The Divergence of Ethics and Professionalism in the Social Media Arena. Plast Reconstr Surg. 2018 Apr;141(4):1071–2. PMID: 29595743 doi: 10.1097/PRS.0000000000004261
213. The do’s and don’ts of using social media. Synergy News. 2016 Feb;15–15.
214. Arzuaga BH, Petty C, Janvier A. The Doctor-Parent Relationship in Pediatrics: trainees’ experiences and perspectives. Acta Paediatr. 2018 Nov 1. PMID: 30383899 doi: 10.1111/apa.14634
215. Sedrak MS, Dizon DS, Anderson PF, Fisch MJ, Graham DL, Katz MS, et al. The emerging role of professional social media use in oncology. Future Oncol. 2017 Jun;13(15):1281–5. PMID: 28589770 doi: 10.2217/fon-2017-0161
216. Bennett KG, Berlin NL, MacEachern MP, Buchman SR, Preminger BA, Vercler CJ. The Ethical and Professional Use of Social Media in Surgery: A Systematic Review of the Literature. Plast Reconstr Surg. 2018 Sep;142(3):388e-98e. PMID: 30148789 doi: 10.1097/PRS.0000000000004692
217. Henderson M, Dahnke MD. The Ethical Use of Social Media in Nursing Practice. Medsurg Nurs. 2015 Jan-Feb 1;24(1):62–4. PMID: 26306360
218. Dorfman RG, Vaca EE, Fine NA, Schierle CF. The Ethics of Sharing Plastic Surgery Videos on Social Media: Systematic Literature Review, Ethical Analysis, and Proposed Guidelines. Plast Reconstr Surg. 2017 Oct;140(4):825–36. PMID: 28953737 doi: 10.1097/PRS.0000000000003695
219. Berquist TH. The evolving world of biomedical publication: Runaway train or merry-go-round? AJR Am J Roentgenol. 2018;210(3):469–72. PMID: 29469622 doi: 10.2214/AJR.17.19431
220. Shah LM. The figley fellowship: Social media in radiology journalism. AJR Am J Roentgenol. 2018 Apr;210(4):705–8. PMID: 29565199 doi: 10.2214/AJR.17.19446
221. Francis L, Holmvall CM, O’Brien LE. The influence of workload and civility of treatment on the perpetration of email incivility. Computers in Human Behavior. 2015 May;46:191–201.
222. Carroll CL, Ramachandran P. The intelligent use of digital tools and social media in practice management. Chest. 2014 Apr;145(4):896–902. PMID: 24687711 doi:10.1378/chest.13-0251
223. ten Hoeve Y, Jansen G, Roodbol P. The nursing profession: Public image, self-concept and professional identity. A discussion paper. J Adv Nurs. 2014;70(2):295–309. PMID: 23711235. doi: 10.1111/jan.12177
224. Ofri D. The passion and the peril: storytelling in medicine. Acad Med. 2015 Aug;90(8):1005–6. PMID: 25692561. doi: 10.1097/ACM.0000000000000672
225. deBronkart D. The patient’s voice in the emerging era of participatory medicine. Int J Psychiatry Med. 2018 Nov;53(5–6):350–60. PMID: 30114957. doi: 10.1177/0091217418791461
226. Saenger AK, Berkwits M, Carley S, Haymond S, Ennis-O’Connor M, Sherbino J, et al. The power of social media in medicine and medical education: Opportunities, risks, and rewards. Clin Chem. 2018 Sep;64(9):1284–90. PMID: 29789353 doi: 10.1373/clinchem.2018.288225
227. Punjabi PP. The professional amateur and the amateurish professional. Perfusion. 2018 Sep;33(6):413–4. PMID: 30126349. doi: 10.1177/0267659118793805
228. Berlin R. The professional ethics of online dating: Need for guidance. J Am Acad Child Adoles Psychiatry. 2014 Sep;53(9):935–7. PMID: 25151414. doi: 10.1016/j.jaac.2014.05.017
229. Ho A, Hee N. The professional online: Stranger in a strange land. Asian Bioethics Review. 2017;9(3):251–5.
230. Peate I. The professional use of social media. British Journal of Healthcare Assistants. 2015 Jul;9(7):350–3.
231. Cooke BK, Goddard ER, Werner TL, Cooke EO, Griffith EEH. The risks and responsible roles for psychiatrists who interact with the media. J Am Acad Psychiatry Law. 2014;42(4):459–68. PMID: 25492072
232. Batt-Rawden S, Flickinger T, Weiner J, Cheston C, Chisolm M. The role of social media in clinical excellence. Clin Teach. 2014;11(4):264–9. PMID: 24917094 doi: 10.1111/tct.12129
233. Perkins L, Dey M, Banerjee S. The third Swansea Perinatal Symposium (SWAPS 2017). Infant. 2017 Sep;13(5):208–10.
234. Pander T, Pinilla S, Dimitriadis K, Fischer MR. The use of Facebook in medical education - A literature review. GMS Z Med Ausbild. 2014 AUg 15;31(3): Doc33. PMID: 25228935 doi: 10.3205/zma000925
235. Sterling M, Leung P, Wright D, Bishop TF. The Use of Social Media in Graduate Medical Education: A Systematic Review. Acad Med. 2017;92(7):1043–56. PMID: 28225466 doi: 10.1097/ACM.0000000000001617
236. Benetoli A, Chen TF, Aslani P. The use of social media in pharmacy practice and education. Res Social Adm Pharm. 2015 Feb;11(1):1–46. PMID: 24814268 doi: 10.1016/j.sapharm.2014.04.002
237. Handy J. This month in JICS. J Intensive Care Soc. 2016;17(4):275. PMID: 28979509 doi: 10.1177/1751143716670297
238. Tweets of the Week. Nursing Standard. 2015 Sep 23;30(4):33–33.
239. Al-Eraky MM. Twelve Tips for teaching medical professionalism at all levels of medical education. Med Teach. 2015 Nov;37(11):1018–25. PMID: 25776227 doi: 10.3109/0142159X.2015.1020288
240. Kind T, Patel PD, Lie D, Chretien KC. Twelve tips for using social media as a medical educator. Med Teach. 2014;36(4):284–90. PMID: 24261897 doi: 10.3109/0142159X.2013.852167
241. Choo EK, Ranney ML, Chan TM, Trueger NS, Walsh AE, Tegtmeyer K, et al. Twitter as a tool for communication and knowledge exchange in academic medicine: A guide for skeptics and novices. Med Teach. 2015 May;37(5):411–6. PMID: 25523012 doi: 10.3109/0142159X.2014.993371
242. Patel SS, Majhail NS. Twitter Use in the Hematopoietic Cell Transplantation Community. Curr Hematol Malig Rep. 2018 Feb;13(1):53–8. PMID: 29374370 doi: 10.1007/s11899-018-0434-1
243. Corden N, Cliffe J. Twitter: what is it all about? Practising Midwife. 2017 Sep;20(8):1–3.
244. Mathews E. Understanding the Code. World of Irish Nursing & Midwifery. 2015 Oct;23(8):46–8.
245. Rice A. Upping the professional game: Elite hygienists do not emerge from social media quarrels. RDH. 2018 May;38(5):50–2.
246. Chan WS, Leung AY. Use of Social Network Sites for Communication Among Health Professionals: Systematic Review. J Med Internet Res. 2018 Mar 28;20(3):e117. PMID: 29592845 doi: 10.2196/jmir.8382
247. Verdusco LM. Using Social Media. Home Healthc Now. 2016 Aug 7;34(7):399–400. PMID: 27348037 doi: 10.1097/NHH.0000000000000410
248. Bergl P, Muntz M. Using social media to enhance health professional education. Clin Teach. 2016 Dec;13(6):399–404. PMID: 27870329 doi: 10.1111/tct.12594
249. Dimov V, Eidelman F. Utilizing social networks, blogging and YouTube in allergy and immunology practices. Expert Rev Clin Immunol. 2015;11(10):1065–8. PMID: 26163316 doi: 10.1586/1744666X.2015.1065731
250. Charlton R. We are all patients. Hong Kong Practitioner. 2014;36(3):81–2.
251. We asked: What is your #1 success tip for brand new massage therapists? Massage Magazine. 2018 Feb;(261):16–16.
252. Macdonald ME, Beaudin A, Pineda C. What do patients think about dental services in Quebec? Analysis of a dentist rating website. J Can Dent Assoc. 2015;81:f3. PMID: 26030597
253. Huddle TS. What does the character of medicine as a social practice imply for professional conscientious objection? Theor Med Bioeth. 2017;38(6):429–45. PMID: 29030798 doi: 10.1007/s11017-017-9426-8
254. Power A. What is social media? British Journal of Midwifery. 2014;22(12):896–7.
255. Salloch S. Who’s afraid of EBM? Medical professionalism from the perspective of evidence-based medicine. Med Health Care Philos. 2017;20(1):61–6. PMID: 27581425 doi: 10.1007/s11019-016-9726-1
256. Why we should all consider a digital detox. Dental Nursing. 2017 Feb;13(2):84–4.
257. Cotton P. Worldly wise: The challenges for global professionals. Med Teach. 2015;37(9):799–802. PMID: 26357979 doi: 10.3109/0142159X.2015.1054798
258. Young physicians more casual about social media. Physician Practice Perspectives. 2016 Nov;35(11):11–2.
259. Redfern Jones J. Your Career in a Nutshell. Nurs Stand. 2014 Nov 12;29(11):63. PMID: 25388740 doi: 10.7748/ns.29.11.63.s50
260. Bakare K. Your lateness speaks volumes. Dental Nursing. 2018 Aug;14(8):382–4.
261. Denecke K, Bamidis P, Bond C, Gabarron E, Househ M, Lau AYS, et al. Ethical Issues of Social Media Usage in Healthcare. Yearb Med Inform. 2015 Aug;24(01):137–47. PMID: 26293861 doi: 10.15265/IY-2015-001
262. Moreno MA, Vaillancourt T. The Role of Health Care Providers in Cyberbullying. Can J Psychiatry. 2017 Jun;62(6):364–7. PMID: 28562092 doi: 10.1177/0706743716684792
263. Harris R, Perkins E, Holt R, Brown S, Garner J, Mosedale S, et al. Contracting with General Dental Services: a mixed-methods study on factors influencing responses to contracts in English general dental practice [Internet]. Southampton (UK): NIHR Journals Library; 2015 [cited 2018 Nov 27]. (Health Services and Delivery Research). Available from: http://www.ncbi.nlm.nih.gov/books/NBK299443/
264. Ozar DT, Sokol DJ, Patthoff DE. Dental ethics at chairside: Professional obligations and practical applications, third edition. 2018. 1 p. (Dental Ethics at Chairside: Professional Obligations and Practical Applications, Third Edition). ISBN: 9781626165533
265. Wiljer D, Thakur A. Ethics, Obligations, and Health Informatics for Clinicians. In: Health Professionals’ Education in the Age of Clinical Information Systems, Mobile Computing and Social Networks. 2017. p. 111–27. ISBN: 9780128093214
266. Uprety S, Ghimire R. Media, social media, and the securitization of mental health problems in Nepal: Yama Buddha’s case study. In: Deconstructing Stigma in Mental Health. 2018. p. 42–69. doi: 10.4018/978-1-5225-3808-0.ch003
267. Jaffe TA, Cron DC, Linzey JR, Nikolian VC, Ibrahim AM. The Digital Age of Academic Medicine: The Role of Social Media. In: Medical and Scientific Publishing: Author, Editor, and Reviewer Perspectives. 2017. p. 189–202. ISBN: 9780128099698
268. Abedi M, Khurram A, Abedi D. RE: learning medical professionalism - the application of appreciative inquiry and social media. Med Educ Online 2020 Dec;25(1):1780723. PMID:32552569
269. Adcock G. Living in the Aisle. The Journal for Nurse Practitioners. 2019 Apr;15(4):321–2. doi: 10.1016/j.nurpra.2019.01.011
270. AHC MEDIA. Updated Ethics Manual Addresses Many New Realities in Clinical Practice. Medical Ethics Advisor 2019 Mar;35(3):36–36.
271. Arif SA, Abrons JP. Promoting cultural sensitivity with the ethical and professional use of social media during global pharmacy experiences. Curr Pharm Teach Learn. 2020 Nov;12(11):1383–6. PMID:32867940
272. Atiyeh BS, Chahine F, Abou Ghanem O. Social Media and Plastic Surgery Practice Building: A Thin Line Between Efficient Marketing, Professionalism, and Ethics. Aesth Plast Surg [Internet] 2020 Sep 22 [access Oct 18, 2020]
273. Balikuddembe JK, Reinhardt JD. Can Digitization of Health Care Help Low-Resourced Countries Provide Better Community-Based Rehabilitation Services?. Phys Ther. 2020 Feb 7;100(2):217–24. PMID:31680158
274. Bansal P, Bingemann TA, Greenhawt M, Mosnaim G, Nanda A, Oppenheimer J, Sharma H, Stukus D, Shaker M. Clinician Wellness During the COVID-19 Pandemic: Extraordinary Times and Unusual Challenges for the Allergist/Immunologist. J Allergy Clin Immunol Pract. 2020 Jun;8(6):1781-90.e3. PMID:32259628
275. Benetoli A, Chen TF, Schaefer M, Chaar B, Hehir T, Aslani P. Professionalism of pharmacists on social network sites. Research in Social & Administrative Pharmacy. 2016 Sep;12(5):e29–e29. doi: 10.1016/j.sapharm.2016.05.073
276. Berney DM. Evolution not revolution. Histopathology. 2019;74(2):217–8. PMID: 30561075
277. Beseler L. The Academy’s Millennial Generation. Journal of the Academy of Nutrition and Dietetics. 2016;116(12):1881. doi: 10.1016/j.jand.2016.09.033
278. Bilal M, Taleban S, Riegler J, Surawicz C, Feld A. The Do’s and Don’ts of Social Media: A Guide for Gastroenterologists. Am J Gastroenterol. 2019;114(3):375–6. PMID: 30333539
279. Blakely MD. Social Media: The Newest Vehicle for Lateral Violence. Med-Surg Matters. 2019 Dec 11;28(6):11–3.
280. Booth R, O’Connor S. Meaningful use of Twitter in nursing education may improve student learning and should be considered as a viable educational tool to assist in the development of digital professionalism. Evid Based Nurs. 2017;20(1):28. PMID: **27903596** doi: 10.1136/eb-2016-102465
281. Bowser KE. The use of pseudoscience to legitimize criticism of young surgeons and reinforce old ways. J Vasc Surg. 2020;72(5):1827–8. PMID: **33011002.**
282. Brackenbury J. Practise what you preach and think before you post. Journal of Aesthetic Nursing. 2020 Mar;9(2):90–1. doi: 10.12968/joan.2020.9.2.90
283. Bradley Radford A. How to Choose the Right Massage Trade Partner. Massage Magazine. 2019 Jun;(277):44–8.
284. Braillon A. The Sunshine Act Has Been for a World that Doesn’t Exist Anymore! American Journal of Medicine. 2018;131(11):e471. doi: 10.1016/j.amjmed.2018.05.036
285. Brasel KJ, Korndorffer JR Jr, Webb TP, Tseng J, Smink DS. Editorial. Journal of Surgical Education. 2020;77(6):1325–6. doi: 10.1016/j.jsurg.2020.08.032
286. Brookes G. To post or not to post: Social media and nursing. Nurs N Z 2017 Mar;23(2):34. PMID:30556990
287. Brown NC, McGee SJ. Conceptualizing Boundaries for the Professionalization of Healthcare Ethics Practice: A Call for Empirical Research. HEC Forum. 2014;26(4):325–41. PMID: **24973870**
288. Carraccio C. Competency 1. Demonstrate sufficient knowledge of the basic and clinically supportive sciences appropriate to pediatrics. Academic Pediatrics. 2014;14(2):S36–7. doi: 10.1016/j.acap.2013.10.007
289. Caruso Brown AE, Arthur JD, Mutrie LH, Lantos JD. Seeking a Second Opinion on Social Media. Pediatrics. 2019 Nov;144(5). PMID:31597691
290. Cole TS, Spetzler RF. Restraint is not the better part of valor. J Neurosurgery. 2020;133(4):1278–9. doi: 10.3171/2020.5.JNS201819
291. Colenda CC. From Retirement to Preferment: Reflections on a Career. Am J Geriatr Psychiatry. 2016;24(9):673–4. PMID: **27569262.**
292. Collier A. How to distance yourself from social media problems. Dental Nursing. 2020 Dec;16(12):584–5. doi: 10.12968/denn.2020.16.12.584
293. Colt HG, Williamson JP. Training in interventional pulmonology: What we have learned and a way forward. Respirology. 2020 Sep;25(9):997–1007. PMID:32453479
294. Cope V. Social Media, E-Professionalism And The Image Of Nursing: How One Nurse Can Reach Many!...5th Annual Worldwide Nursing Conference (WNC2017), 24th–25th July 2017, Singapore. Annual Worldwide Nursing Conference 2017 Jan;160–4. doi: 10.5176/2315-4330_WNC17.129
295. de Peralta TL, Farrior OF, Flake NM, Gallagher D, Susin C, Valenza J. The Use of Social Media by Dental Students for Communication and Learning: Two Viewpoints: Viewpoint 1: Social Media Use Can Benefit Dental Students’ Communication and Learning and Viewpoint 2: Potential Problems with Social Media Outweigh Their Benefits for Dental Education. J Dent Educ. 2019 Jun;83(6):663–8. PMID:30910932
296. Dietl CA, Russell JC. Effects of Technological Advances in Surgical Education on Quantitative Outcomes From Residency Programs. J Surg Educ. 2016;73(5):819–30. PMID: **27184181**
297. Dike CC, Candilis P, Kocsis B, Sidhu N, Recupero P. Ethical Considerations Regarding Internet Searches for Patient Information. Psychiatr Serv. 2019 Apr 1;70(4):324–8. PMID:30651058
298. Dubin JM, Greer AB, Patel P, [et al.]. Global survey evaluating drawbacks of social media usage for practising urologists. BJU International 2020;126(1):7–8. PMID: 3214793
299. Duma N, Maingi S, Tap WD, Weekes CD, Thomas CR. Establishing a Mutually Respectful Environment in the Workplace: A Toolbox for Performance Excellence. Am Soc Clin Oncol Educ Book 2019 Jan;39:e219–26. PMID:31099664
300. Economides JM, Choi YK, Fan KL, Kanuri AP, Song DH. Are We Witnessing a Paradigm Shift?: A Systematic Review of Social Media in Residency. Plast Reconstr Surg Glob Open. 2019 Aug;7(8):e2288. PMID: 31592016. doi: 10.1097/GOX.0000000000002288
301. Emanuel EJ. The Inevitable Reimagining of Medical Education. JAMA - Journal of the American Medical Association. 2020;323(12):1127–8. PMID: **32105294 doi:** 10.1001/jama.2020.1227
302. Ennis-O-Connor M, Mannion R. Social media networks and leadership ethics in healthcare. Healthc Manage Forum. 2020 May;33(3):145–8. PMID:31884833
303. European S of R (ESR). Summary of the proceedings of the International Forum 2019: “What are the strategies to engage the young generation.” Insights into Imaging. 2020;11(1). doi: 10.1186/s13244-019-0827-2
304. Fable S. The Instructor-or-influencer Challenge. IDEA Fitness Journal 2019 Mar;16(3):47–50.
305. Favaretto M, Shaw D, De Clercq E, Joda T, Elger BS. Big Data and Digitalization in Dentistry: A Systematic Review of the Ethical Issues. Int J Environ Res Public Health. 2020 Apr 6;17(7). PMID:32268509
306. Flynn J. How to Protect Your License to Practice. Imprint. 2019 Jan;66(1):31–7.
307. Friedman LH. EDITORIAL: Rethinking Problem-Solving in an Era of COVID-19. Journal of Health Administration Education. 2020;37(1):1–5.
308. Friedman SH, Martinez RP. Boundaries, Professionalism, and Malpractice in Psychiatry. Focus (Am Psychiatr Publ). 2019 Oct;17(4):365–71. PMID:32047383
309. Furnas HJ. Discussion: The Public’s Preferences on Plastic Surgery Social Media Engagement and Professionalism: Demystifying the Impact of Demographics. Plast Reconstr Surg. 2019 Feb;143(2):631–632. PMID:30688912
310. Gabbard GO. Digital Professionalism. Acad Psychiatry. 2019 Jun;43(3):259–63. PMID:30324397
311. Gaetke-Udager K, Magid D, Smith M, Kumar V, Khaja M, Dunnick NR. Response to Strategies for Recruiting Medical Students to Radiology. Acad Radiol. 2020;27(9):1337. PMID: 31839565 doi: 10.1016/j.acra.2019.11.014
312. Gandhi JS. Portrayal of a profession: The art of Norman Rockwell. Am J Med. 2014;127(10):1024–5. PMID: **24979743** doi: 10.1016/j.amjmed.2014.06.019
313. Gierke T. Etiquette Tips that “Virtually” Everyone Can Use. ASBN Update. 2020 Nov;24(6):10–10.
314. Gih D, Kurtz BP, Liu HY. Twitter for intermediate users: hands-on skills for networking, professionalism, and promotion. Journal of the American Academy of Child & Adolescent Psychiatry. 2020 Oct 2;59(10):S356–S356. doi: 10.1016/j.jaac.2020.07.879
315. Gisselman AS. Editorial. Physical Therapy Reviews. 2019 Jun;24(3/4):59–59.
316. Gloviczki P, Lawrence PF. Commitment to increase diversity and address unconscious bias. J Vasc Surg. 2020;72(5):1515. PMID: **33011001** doi: 10.1016/j.jvs.2020.08.001
317. Gloviczki P, Lawrence PF. Farewell to a year of challenges and opportunities. J Vasc Surg. 2020;72(6):1837–42. PMID: **33222821** doi: 10.1016/j.jvs.2020.10.016
318. Grindrod K. Technology and social media applications in pharmacy practice. In:Encyclopedia of Pharmacy Practice and Clinical Pharmacy. Elsevier; 2019. p. 352–8. doi: 10.1016/B978-0-128-12735-3.00165-5
319. Hartley MT, Bourgeois PJ. The Commission on Rehabilitation Counselor Certification Code of Ethics: An Emerging Approach to Digital Technology. Rehabilitation Research, Policy & Education. 2020 Jun;34(2):73–85. doi: 10.1891/RE-19-04
320. Hayhoe B, Papanikitas A, Sajid I. Ethical issues in the use of online social media forums by GPs. Br J Gen Pract. 2019;69(681):203–4. PMID: **30923160** doi: 10.3399/bjgp19X702101
321. Heitkamp DE, Cooke EA, Deitte LA, [et al.]. Radiology Program Directors Should Have an Active Presence on Twitter. J Am Coll Radiol. 2020;17(2):293–5. PMID: **31843345** doi: 10.1016/j.jacr.2019.10.014
322. Hennessy CM, Royer DF, Meyer AJ, Smith CF. Social Media Guidelines for Anatomists. Anat Sci Educ. 2020 Jul;13(4):527–39. PMID: **32043732**
323. Hetzler PT, Wang J, Fan KL, Song DH. Conceptualizing Professionalism in Social Media: A Framework for Evaluation. Plast Reconstr Surg. 2019 Jun;143(6):1318e–9e. PMID:30907794
324. Hilty D, Chan S, Torous J, Luo J, Boland R. A Framework for Competencies for the Use of Mobile Technologies in Psychiatry and Medicine: Scoping Review. JMIR Mhealth Uhealth. 2020 Feb 21;8(2):e12229. PMID:32130153
325. Houle A-M, Ouimet G, Beiko D, Oake JS, Davies TO. Exploring the business of urology: Influence management and political skills. Can Urol Assoc J.2017;11(8):227–9. PMID: **28798818**
326. Hughes BA, Stallard J, West CC. The use of WhatsappⓇ as a way to deliver plastic surgery teaching during the COVID-19 pandemic. J Plast Reconstr Aesthet Surg. 2020;73(7):e1–e2. PMID: **32505627** doi: 10.1016/j.bjps.2020.05.034
327. Jackson J. Social media: blurred lines between personal and professional behaviour. Practice Nurse. 2019 Apr;49(4):14–16.
328. Jawed S, Mahboob U, Yasmeen R. Digital professional identity: Dear Internet! Who am I?. Educ Health (Abingdon). 2019;32(1):33. PMID: **31512590** doi: 10.4103/efh.EfH_232_17
329. Jildeh TR, Okoroha KR, Guthrie ST, Parsons TW. Social Media Use for Orthopaedic Surgeons. JBJS Rev. 2019 Mar;7(3):e7. PMID:30920481
330. Kardashian A, May FP. Empowering early career female gastroenterologists and hepatologists. Nat Rev Gastroenterol Hepatol. 2019;16(11):644–5. PMID: **31570808** doi: 10.1038/s41575-019-0216-9
331. Karim S. Professionalism through a lens of inclusion and diversity. J Vasc Surg. 2020;72(5):1822–3. PMID**: 32890721** doi: 10.1016/j.jvs.2020.07.074
332. Kirsch NR. In the Rough: A PT-patient relationship built on golf goes off course. PT in Motion 2020. Mar;12(2):16–9.
333. Kling S. Challenging beliefs: Social media and professionalism. Current Allergy and Clinical Immunology. 2019;32(3):176–8.
334. Knestrick J. May Laundry and Social Media. Journal for Nurse Practitioners. 2019 May;15(5):A10–A10. doi: 10.1016/j.nurpra.2019.04.008
335. Knott J. Better Instagram results. Dermatology Times. 2019 Oct;40(10):54–6.
336. Knottnerus JA, Tugwell P. Promoting transparency of research and data needs much more attention. J Clin Epidemiol. 2016;70:1–3. PMID: **26821973**
337. Kocemba P, Lasota M, Sroka NH, Feleszko W. Facebook-based medicine, or the doctor’s professional image on the Internet. Pediatria i Medycyna Rodzinna. 2015;11(3):328–38. doi: 10.15557/PiMR.2015.0032
338. Kornowski R. Contemporary structural heart disease registries: when professional networking meets clinical research. EuroIntervention. 2019;15(9):e745–7. PMID: 31579011
339. Koziebrocki J. Advertising, Public Statements, And Professionalism In Dentistry: Zuk v. Alberta Dental Association and College. Oral Health Journal. 2019 Feb;109(2):94–5.
340. Kung JW, Wigmore SJ. How surgeons should behave on social media. Surgery (Oxf). 2020 Oct;38(10):623–6. PMID:32904552
341. Link C, Rebman K, Christian A, Jung Campbell C. Speak Your Mind. From Facebook. Massage & Bodywork. 2019 Feb 1;34(1):19–19.
342. Little JS, Romee R. Tweeting from the Bench: Twitter and the Physician-Scientist Benefits and Challenges. Curr Hematol Malig Rep. 2020 Dec;15(6):419–23. PMID:33179209
343. Liu HY, Beresin EV, Chisolm MS. Social Media Skills for Professional Development in Psychiatry and Medicine. Psychiatr Clin North Am. 2019 Sep;42(3):483–92. PMID: **31358127**
344. Loeb S, Murphy DG. Evaluating and Optimizing the Use of Social Media in Urology. Eur Urol Focus. 2020;6(3):425–6. PMID: **32127328**
345. Long KN. The power of social media: A call for change in professional perspective. J Vasc Surg. 2020;72(5):1820–1. PMID: **32889068**. doi: 10.1016/j.jvs.2020.07.072
346. Luc JGY, Antonoff MB. A Cardiothoracic Surgeon’s Playbook for Social Media and Digital Scholarship. Innovations (Phila.). 2020;15(5):416–22. PMID: 32894681
347. Mandelbaum M, Taub PJ. “The Name Game”: A Case against Hidden Identity on Social Media during the Plastic Surgery Interview Season. Plast Reconstr Surg. 2020 Aug;146(2):245e–6e. PMID:32740628
348. Mar C, Chang S, Forster B. Remedial Training for the Radiology Resident: A Template for Optimization of the Learning Plan. Acad Radiol 2015;22(2):240–6. PMID: 25481977
349. Margioris AN. Hormones—International Journal of Endocrinology and Metabolism: a new publisher, a step forward. Hormones (Athens). 2018;17(1):1-2. PMID: **29858869**
350. Mazer BL, Fuller MY, Lepe M, [et al.]. Social Media in Pathology: Continuing a Tradition of Dialogue and Education. Arch Pathol Lab Med. 2018 Aug;142(8):889–90. PMID: **30040463**. doi: 10.5858/arpa.2018-0057-LE
351. McCarthy CP, DeCamp M, McEvoy JW. The Reply. Am J Med. 2018;131(11):e473. PMID: 30392640 doi:10.1016/j.amjmed.2018.06.024
352. McGrath L, Swift A, Clark M, Bradbury-Jones C. Understanding the benefits and risks of nursing students engaging with online social media. Nurs Stand. 2019 Sep 27;34(10):45–9. PMID:31544374
353. Minhas RS. The elephant in the Zoom: Recognizing and reconciling my internalized racism. CMAJ. 2020;192(40):E1169–70. PMID: 33020126 doi: 10.1503/cmaj.201737
354. Montgomery T, Berns JS, Braddock CH Iii. Transparency as a Trust-Building Practice in Physician Relationships with Patients. JAMA - Journal of the American Medical Association 2020;324(23):2365–6. PMID: **33320234** doi: 10.1001/jama.2020.18368
355. Mooney CJ, Peyre SE, Clark NS, Nofziger AC. Rapid transition to online assessment: Practical steps and unanticipated advantages. Med Educ. 2020 Sep;54(9):857–8. PMID: 32403189 doi: 10.1111medu.14225
356. Nalabandian T, Ireland ME. Genre-typical narrative arcs in films are less appealing to lay audiences and professional film critics. Behav Res Methods 2019 Aug;51(4):1636–50. PMID:30506118
357. Nemetz ETA, Urbach DR, Devon KM. The art of surgery: Balancing compassionate with virtual care. J Med Internet Res. 2020;22(8):e22417. PMID: 32852276. doi: 10.2196/22417
358. Oehler RL. On Measles, Vaccination, Social Media Activism, and How to Win Back Our Role as Our Patients’ Best Advocates. Clin Infect Dis. 2020 Jan 15;70(2):338–40. PMID: 31309980 doi: 10.1093/cid/ciz656
359. Ogle K, Roche C, Pourmand A. On first-pass, twitter response is inferior to expectations. Am J Emerg Med. 2020;38(1):151–2. PMID: 31685306. doi:10.1014/j.ajem.2019.158438
360. Phelon S. Responsible Use of Social Media. The North Dakota Nurse. 2020 Jan;89(1):9–16.
361. Priest M, Adamson M, Montagna Heckert M, Deighan K, Vincent A. MASSAGE Magazine Social Networking. Massage Magazine 2015 Apr;(227):16–16.
362. Prober AS, Ledermann E, Norbash A, Mehan WA Jr, Bedi HS. Fulfilling the health care economics milestones: Adopting an online curriculum for radiology residency programs. J Am Coll Radiol. 2015;12(3):314–7. PMID: 25743927 doi: 10.1016/j.jacr.2014.09.09.030
363. Qiu L, Morse A, Di W, Song L, [et al.]. Management of gynecology patients during the coronavirus disease 2019 pandemic: Chinese expert consensus. Am J Obstet Gynecol. 2020;223(1):3–8. PMID: 32416154 doi: 10.1016/j.ajog.2020.05.024
364. Redmond CE, Crawford R, O’neill DC, Lee MJ. Social media as a learning resource for medical students. Ir Med J. 2020;113(4):64. PMID: 32268057
365. Riley B. Incorporating a Standardized Online Professionalism Curriculum in Osteopathic Medical School. J Am Osteopath Assoc. 2019 Feb 1;119(2):112–5. PMID:30688348
366. Rimmer A. A patient has complained about me online. What should i do?. BMJ. 2019;366:15705. PMID: 31570371 doi: 10.1136/bmj.15705
367. Rose CD. Make O.D.-to-O.D. Referrals Successful: Why and how to build a team of local optometrists to meet patients’ needs. Optometric Management. 2020 Apr;55(4):28–9.
368. Ross P, Cross R. Rise of the e-Nurse: the power of social media in nursing. Contemp Nurse. 2019 May 4;55(2–3):211–20. PMID: **31280696**
369. Rossella DL, Patrizia D, Cinzia G, Gianluca LC, Giuseppe C. The patient-physician relationship in the face of oncological disease: A review of literature on the emotional and psychological reactions of patients and physician. Acta Medica Mediterranea. 2016;32(6):1827–33. doi: **10.19193/0393-6384_2016_6_170**
370. Rothman DJ, Blumenthal D, Thibault GE. Medical Professionalism In An Organizational Age: Challenges And Opportunities. Health Aff (Millwood). 2020 Jan;39(1):108–14. PMID:31905069
371. Runyan A. Studies looking into “professionalism” would benefit from self-reflection and integrity of the research process. J Vasc Surgery. 2020;72(5):1823–4. PMID: **32958314** doi: 10.1016/j.jvs.2020.07.075
372. Sawyer NT, Nguyen M. HIPAA Versus CIPA (California Invasion of Privacy Act): Are Physicians Protected from Live Social Media Streaming in the Emergency Department? West J Emerg Med. 2020 May;21(3):583–5. PMID: **32421504**
373. Schloss RW Jr. The highly visible radiologist: Ethics of social media use in radiology. Clin Imaging. 2020;67:189–90. PMID: **32836099**. doi: 10.1016/j.clinimag.2020.08.002.
374. Schoenbrunner A, Gosman A, Bajaj AK. Framework for the Creation of Ethical and Professional Social Media Content. Plast Reconstr Surg. 2019 Jul;144(1):118e–25e. PMID:31246836
375. Schwartz ES. A welcoming environment. Applied Radiology. 2020;49(2):8.
376. Sewpaul V, Henrickson M. The (r)evolution and decolonization of social work ethics: The Global Social Work Statement of Ethical Principles. International Social Work. 2019 Nov;62(6):1469–81. doi: 10.1177/0020872819846238
377. Shah S, Topf J. Mentorship in the digital age: Nephrology social media collective internship. Clin J Am Soc Nephrol. 2019;14(2):294–6. PMID: **30647092**
378. Shanahan B. Top Five Lessons Learned in My First Year as a New Professional. Audiology Today. 2018 Jun 5;30(3):10–1.
379. Skourou C, Sherouse GW, Bahar N, [et al.]. Code of ethics for the American Association of Physicists in Medicine (Revised): Report of Task Group 109. Med Phys. 2019 Apr;46(4):e79–e93. PMID:30570754
380. Smalldridge A. Allow GPs and consultants to speak to each other again. BMJ (Online) 2015;350:h1623. PMID: **25813648**
381. Stamp N, Mitchell R, Fleming S. Social media and professionalism among surgeons: Who decides what’s right and what’s wrong? J Vasc Surg. 2020 Nov;72(5):1824–6. PMID:32958315
382. Steers M-LN, Gallups SF. Ethical tipping point: Nurses’ presence on social media. Nursing. 2020 Dec;50(12):52–4. PMID: **33497095**
383. Stephens S. 7 tips to get the most from your posts. Dermatology Times. 2019 Sep;40(9):76–7.
384. Ta AQ, Tang CG. Social Media among Medical Students and Personnel Here to Stay. Otolaryngol Head Neck Surg. 2018 Jun;158(6):972–3. PMID:29405835
385. Talsma J. Social media presence can benefit pharmacies: Responsible use necessary to avoid pitfalls, such as HIPAA violations. Drug Topics. 2014;(Dec 2014).
386. Tan J, Bernard B, Chung J, [et al.]. Invited Commentary: ACCESS Open Minds National Youth Council. Early Interv Psychiatry. 2019;13(S1):65–7. PMID: **31243910** doi: 10.1111/eip.12820
387. Taylor J, Loeb S. Guideline of guidelines: social media in urology. BJU Int. 2020 Mar;125(3):379–82. PMID:31631471
388. Teh J, Ashcroft J. “Tweet’’-format reflective writing: A modern needs assessment. Am J Surg. 2019;218(3):664–5. PMID: **30389121** doi: 10.1016/j.amjsurg.2018.10.032
389. Terry M. Managing Your Online Presence: Here are some tips on handling your online marketing. Podiatry Management. 2019 Jan;38(1):93–8.
390. Tracy DK, Joyce DW, Albertson DN, Shergill SS. Kaleidoscope. Br J Psychiatry. 2020;217(4):593–4. PMID: **33100242** doi: 10.1192/bjp.2020.155
391. Trueger NS, Farcas A. If a tweet falls in a conference ... CJEM. 2020 May;22(3):275–6. PMID: **32456736** doi: 10.1017/cem.2020.41
392. Trueman S. Blurred line between private and professional. Nursing Review. 2017 Feb 1;(1):24–5.
393. Truong H, Salib A, Rowe CK. The Use of Social Media in Pediatric Urology-Forging New Paths or Crossing Boundaries? Curr Urol Rep. 2019 Oct 16;20(11):72. PMID:31620926
394. Udawatta M, Ong V, Duong C, [et al.]. In reply: Patient satisfaction ratings of male and female residents across subspecialties. Neurosurgery. 2020;87(4):E530–2. doi: 10.1093/neuros/nyaa324
395. Vallejo MC, Attaallah AF, Nield LS, Elmo RM, Cottrell S, Ferrari ND. An online web-based assessment tool to monitor graduate medical trainee professionalism and supervision. Int J Med Educ. 2018 Jun 22;9:173–4. PMID:29936492 doi: 10.5116/ijme.5b1e.21ca
396. Van Witzenburg M. Social Media: Proceed with caution. RDH. 2020 Apr;40(4):20–2.
397. Varghese TK, Entwistle JW, Mayer JE, Moffatt-Bruce SD, Sade RM, Cardiothoracic Ethics Forum. Ethical Standards for Cardiothoracic Surgeons’ Participation in Social Media. Ann Thorac Surg. 2019 Sep;108(3):666–70. PMID:31262490 doi: 0.1016/j.athoracsur.2019.04.003
398. Villa-García L, Rodriguez Blanco O. Social networks in health care: Ethical implications and nursing professionalism. Enferm Clin. 2020 Feb;30(1):66–7. PMID:31640942 doi: 10.1016/j.enfcli.2019.08.006
399. Wagner CR. A Great Ride. Mol Pharm. 2018;15(12):5445. PMID: **30985144** doi: 10.1021/acs.molpharmaceut.8b01121
400. Wardrope A, Reuber M. Medicine and the media: the ethics of virtual medical encounters. Clin Med (Lond). 2019 Jan;19(1):11–5. PMID:30651238 doi: 10.7861/clinmedicine.19-1-11
401. Weddle M. Rock paper scissors. Patient Educ Couns. 2014;94(1):138–9. PMID**: 24144676** doi: 10.1016/j.pec.2013.09.011
402. Werner R. Proceed at Your Own Peril: 13 Terrible Tips for MTs. Massage & Bodywork. 2019 Apr 3;34(2):15–7.
403. Weston R, Crandall M, Ferrada P. Social Media and Free Open Access Medical (FOAM) Education. Current Surgery Reports. 2019;7(2):4. doi: 10.1007/s40137-019-0224-2
404. Wexner SD, Hoyt DB, Cortés-Guiral D. The American College of Surgeons Response to the COVID-19 Pandemic (Part III): Leadership in a Time of Crisis. Am Surg. 2020 Jul;86(7):762–5. PMID:32916069 doi: 10.1177/0003134820940772
405. Willard B. Social Media and Nursing: Where to Draw the Line with Professionalism. Nebraska Nurse. 2019 May;52(2):2–2.
406. Wissinger CL, Stiegler Z. Using the Extended Parallel Process Model to Frame E-Professionalism Instruction in Healthcare Education. Teach Learn Med. 2019 May 27;31(3):335-41. PMID: **31157561** doi: 10.1080/10401334.2018.1528155
407. Wong XL, Liu RC, Sebaratnam DF. Evolving role of Instagram in #medicine. Intern Med J. 2019 Oct;49(10):1329–1332. PMID:31602768 doi: 10.1111/imj.14448
408. Wood ML, Forgie SE. A First Step to Blended Delivery: Introducing an Online Component to an Infectious Diseases Course Using a Photography-Based Social Media Platform. Medical Science Educator. 2015;25(2):101–3.
409. Top 10 Reasons for Volunteering for the American Society of Echocardiography (ASE). Journal of the American Society of Echocardiography. 2015;28(8):A17. doi: 10.1016/j.echo.2015.06.008
410. Publishing in an ASPET journal – What’s in it for you?. Journal of Pharmacology and Experimental Therapeutics. 2018;365(1):94–5. doi: 10.1124/jpet.117.248534
411. Optimizing Social Media Use in Health Care: The Mayo Clinic Social Media Network. EP Lab Digest 2018 Nov;18(11).
412. TOP 4 Factors in Choosing Online Continuing Education. Massage Magazine. 2019 Feb;(273):32–3.
413. Non-alcoholic fatty liver disease: hiding in plain sight. Practice Nurse. 2019 Apr;49(4):24–8.
414. Tricia B. Bent-Goodley. Progress, Hope, and Gratitude: From One Social Worker to Another (A Farewell Message). Soc Work. 2019 Apr;64(2):101–2. PMID: 30809668 doi: 10.1093/sw/swz006
415. Cole L. Got Civility? ASHA Digital Toolkit Promotes Respectful Online Communication. ASHA Leader. 2019 Jun;24(6):6–64.
416. Pledge of Professional Civility and DPG/MIG Online Netiquette Guidelines. Oncology Nutrition Connection. 2019 Jul;26(3):26–7.
417. HCPs need to be cautious on social media. Canadian Chiropractor. 2019 Aug 7;24(5):8–8.
418. Why We Tweet: Neurologists Tell All. Neurology Today. 2020 Jan 23;20(2):20–20.
419. A Guide To Using Social Media. Oregon State Board of Nursing Sentinel. 2020 May;39(2):6–9.
420. Tips to help support patients using technology during the COVID-19 pandemic. Case Management Monthly. 2020 Aug;17(8):2–4.
421. Parkhurst DC. A Call for Transformation in Physician Assistant Education. J Physician Assist Educ. 2015 Jun;26(2):101–5. PMID: 25933017 doi: 10.1097/JPA.0000000000000017
422. Cohn RJ, Plack MM. A Cloud With a Silver Lining: Helping Students Learn About Professionalism. Teach Learn Med. 2017 Jul-Sep;29(3):304–12. PMID: 28632008 doi: 10.1080/10401334.2016.1274658
423. Boyd L, Lawson C, DiProspero L, Tan K, Matthews K, Singh N. Use of Online Media for Professional Development Amongst Medical Radiation Practitioners in Australia and Canada. Journal of Medical Imaging and Radiation Sciences. 2018;49(2):187–93. doi: https://doi.org/10.1016/j.jmir.2018.03.005
424. Regnier V. A compelling societal problem makes for a good professional life. Annual Review of Gerontology and Geriatrics. 2018;38(1):239–55.
425. Sabin JE, Skimming K. A framework of ethics for telepsychiatry practice. Int Rev Psychiatry. 2015;27(6):490–5. PMID: 26493214 doi: 10.3109/09540261.2015.1094034
426. Alkhatib OJ. A Moral (Normative) Framework for the Judgment of Actions and Decisions in the Construction Industry and Engineering: Part II. Sci Eng Ethics. 2017;23(6):1617–41. PMID: 27913987 doi: 10.1007/s11948-016-9851-5
427. Levy RM. A Personal Evaluation of Changing Social Contracts in Medicine: Reflections of Medicine in the Media. Neuromodulation. 2017 Jan;20(1):1–6. PMID: 28101994 doi: 10.1111/ner.12570
428. Dimitri D, Gubert A, Miller AB, Thoma B, Chan T. A Quantitative Study on Anonymity and Professionalism within an Online Free Open Access Medical Education Community. Cureus. 2016 Sep 18;8(9):e788. PMID: 28638746 doi: 10.7759/cureus.788
429. Bell E. A Room with a view of integrity and professionalism: personal reflections on teaching responsible conduct of research in the neurosciences. Sci Eng Ethics. 2015;21(2):461–9. PMID: 24760542 doi: 10.1007/s11948-014-9545-9
430. Search KR, Tolle SL, McCombs GB, Arndt A. A Study of Visible Tattoos in Entry-Level Dental Hygiene Education Programs. J Dent Hyg. 2018 Feb;92(1):6–15. PMID: 29500280
431. Dapueto JJ, Viera M, Samenow C, Swiggart WH, Steiger J. A Tale of Two Countries: Innovation and Collaboration Aimed at Changing the Culture of Medicine in Uruguay. HEC Forum. 2018;30(4):329–39. PMID: 29752645 doi: 10.1007/s10730-018-9351-x
432. Browne F, Rolfe K, Currie A, Walker T, Roff S. Adapting and feasibility testing pre-registration e-learning resources for Professionalism in Osteopathy in the UK. International Journal of Osteopathic Medicine. 2015;18(1):50–62.
433. Colvin J, French J, Siperstein A, Capizzani TR, Krishnamurthy VD. Addressing Professionalism, Social, and Communication Competencies in Surgical Residency Via Integrated Humanities Workshops: A Pilot Curriculum. J Surg Educ. 2018 Jun;75(3):589–93. PMID: 29056347 doi: 10.1016/j.jsurg.2017.09.035
434. Rafla M, Carson NJ, DeJong SM. Adolescents and the Internet: what mental health clinicians need to know. Curr Psychiatry Rep. 2014;16(9):472. PMID: 25070673 doi: 10.1007/s11920-014-0472-x
435. Railey K, Tuttle B, Weiss J. Are You SURE You Want to Send That? A Model for Email Professionalism in Medical Education. J Physician Assist Educ. 2017 Dec;28(4):205–9. PMID: 29189651 doi: 10.1097/JPA.0000000000000165
436. Wagner JP, Chen DC, Donahue TR, Quach C, Hines OJ, Hiatt JR, et al. Assessment of resident operative performance using a real-time mobile Web system: preparing for the milestone age. J Surg Educ. 2014 Dec;71(6):e41-46. PMID: 25037504 doi: 10.1016/j.jsurg.2014.06.008
437. MacVarish K, Kenefick H, Fidler A, Cohen B, Orellana Y, Todd K. Building Professionalism Through Management Training: New England Public Health Training Center’s Low-Cost, High-Impact Model. J Public Health Manag Pract. 2018 Oct;24(5):479–86. PMID: 28991053 doi: 10.1097/PHH.0000000000000693
438. Brandt AM, Rettig SA, Kale NK, Zuckerman JD, Egol KA. Can a Clinician-Scientist Training Program Develop Academic Orthopaedic Surgeons? One Program’s Thirty-Year Experience. J Surg Educ. 2018 Aug;75(4):1039–44. PMID: 29102560 doi: 10.1016/j.jsurg.2017.10.003
439. Barnhoorn PC, Bolk JH, Ottenhoff-de Jonge MW, van Mook WN, de Beaufort AJ. Causes and characteristics of medical student referrals to a professional behaviour board. Int J Med Educ. 2017 Jan 15;8:19–24. PMID: 28088777 doi: 10.5116/ijme.584b.d591
440. Francis A, Hills C, MacDonald-Wicks L, Johnston C, James D, Surjan Y, et al. Characteristics of an ideal practice educator: Perspectives from practice educators in diagnostic radiography, nuclear medicine, nutrition and dietetics, occupational therapy and physiotherapy and radiation therapy. Radiography. 2016 Nov;22(4):287–94.
441. Adams S, Schiffers P. Co-constructed health narratives during a “media event”: The case of the first Dutch Twitter heart operation. Digit Health. 2017 Jun 21;3:2055207617712046. PMID: 29942602 doi: 10.1177/2055207617712046
442. Brown NC, McGee SJ. Conceptualizing boundaries for the professionalization of healthcare ethics practice: A call for empirical research. HEC Forum. 2014;26(4):325–41. PMID: 24973870 doi: 10.1007/s10730-014-9240-x
443. Cheung JC. Confronting the Challenges in Using Social Network Sites for Cyber Youth Work. Soc Work. 2016 Apr;61(2):171–3. PMID: 27180529
444. Kaijser M, van Ramshorst G, van Wagensveld B, Pierie JP. Current Techniques of Teaching and Learning in Bariatric Surgical Procedures: A Systematic Review. J Surg Educ. 2018 Jun;75(3):730–8. PMID: 29033273 doi: 10.1016/j.jsurg.2017.09.023
445. Sharifian N, Bedos C, Wootton J, El-Murr IJ, Charbonneau A, Emami E. Dental Students’ Perspectives On Rural Dental Practice: A Qualitative Study. J Can Dent Assoc. 2015;81:f23. PMID: 26679337
446. Fan Y, Zhang X, Xie X. Design and Development of a Course in Professionalism and Ethics for CDIO Curriculum in China. Sci Eng Ethics. 2015;21(5):1381–9. PMID: 25230906 doi: 10.1007/s11948-014-9592-2
447. Ajjawi R, Barton KL, Dennis AA, Rees CE. Developing a national dental education research strategy: priorities, barriers and enablers. BMJ Open. 2017 29;7(3):e013129. PMID: 28360237 doi: 10.1136/bmjopen-2016-013129
448. Bluteau P, Clouder L, Cureton D. Developing interprofessional education online: An ecological systems theory analysis. J Interprof Care. 2017 Jul;31(4):420–8. PMID: 28471258 doi: 10.1080/13561820.2017.1307170
449. Bodell S, Sarah A. Developing online professional networks for undergraduate occupational therapy students: An evaluation of an extracurricular facilitated blended learning package. British Journal of Occupational Therapy. 2014;77(6):320–3.
450. Morokhovets HY, Lysanets YV. Developing the professional competence of future doctors in the instructional setting of higher medical educational institutions. Wiad Lek. 2017;70(1):101–4. PMID: 28343203
451. Dang J, Cole JC, Burgess SM, Yang M, Daniels SR, Walt JG. Development and Validation of the Eyelash Satisfaction Questionnaire. Aesthet Surg J. 2016 Feb;36(2):221–8. PMID: 26691738 doi: 10.1093/asj/sjv154
452. Vogel A. Does the Fitness Industry Have a Body Image Problem? IDEA Fitness Journal. 2018 Feb;15(2):34–43.
453. Humphrey D. Don’t “like” unprofessionalism. RDH. 2016 Feb;36(2):24–103 .
454. Ohta R, Kaneko M. Effects of practicing in remote Japanese islands on physicians’ control of negative emotions: A qualitative study. J Rural Med. 2017 Nov;12(2):91–7. PMID: 29255525 doi: 10.2185/jrm.2934
455. Lanken PN, Novack DH, Daetwyler C, Gallop R, Landis JR, Lapin J, et al. Efficacy of an internet-based learning module and small-group debriefing on trainees’ attitudes and communication skills toward patients with substance use disorders: results of a cluster randomized controlled trial. Acad Med. 2015 Mar;90(3):345–54. PMID: 25295964 doi: 10.1097/ACM.0000000000000506
456. Saran JS, Papadakos PJ. Electronic Distraction in the Operating Room: A Major Safety Issue. ORNAC Journal. 2018 Jun;36(2):12–27.
457. Papadakos PJ. Electronic distractions of the respiratory therapist and their impact on patient safety. Respir Care. 2014;59(8):1306–9. PMID: 24962225 doi: 10.4187/respcare.03483
458. Resnik DB, Elmore SA. Ensuring the Quality, Fairness, and Integrity of Journal Peer Review: A Possible Role of Editors. Sci Eng Ethics. 2016;22(1):169–88. PMID: 25633924 doi: 10.1007/s11948-015-9625-5
459. Starke M, Rosqvist HB, Kuosmanen J. Eternal Children? Professionals’ Constructions of Women with an Intellectual Disability Who are Victims of Sexual Crime. Sexuality and Disability. 2016;34(3):315–28.
460. Eggleson KK, Fox MD. Ethical Challenges of Informal Medicine in the Internet Age. Am J Bioeth. 2016 Nov;16(11):51–2. PMID: 27749181 doi: 10.1080/15265161.2016.1222011
461. Maben-Feaster RE, Stansfield RB, Opipari A, Hammoud MM. Evaluating Patient Perspectives of Provider Professionalism on Twitter in an Academic Obstetrics and Gynecology Clinic: Patient Survey. J Med Internet Res. 2018 Mar 12;20(3):e78. PMID: 29530838 doi: 10.2196/jmir.8056
462. Hartranft TH, Yandle K, Graham T, Holden C, Chambers LW. Evaluating Surgical Residents Quickly and Easily Against the Milestones Using Electronic Formative Feedback. J Surg Educ. 2017 Apr;74(2):237–42. PMID: 27746056 doi: 10.1016/j.jsurg.2016.09.006
463. Tahir M, Yasmeen R, Khan RA. Exploring practices of dermatologists in ethical dilemmas in Pakistan: A narrative analysis. Pak J Med Sci. 2018;34(2):374–9. PMID: 29805411 doi: 10.12669/pjms.342.14328
464. Drouin M, O’Connor KW, Schmidt GB, Miller DA. Facebook fired: Legal perspectives and young adults’ opinions on the use of social media in hiring and firing decisions. Computers in Human Behavior. 2015 May;46:123–8.
465. Doshi AM, Somberg M, Rosenkrantz AB. Factors Influencing Patients’ Perspectives of Radiology Imaging Centers: Evaluation Using an Online Social Media Ratings Website. J Am Coll Radiol. 2016 Feb;13(2):210–6. PMID: 26521969 doi: 10.1016/j.jacr.2015.08.020
466. Short C. Fired! Me? Now what? How to rally from the gut punch to your career. RDH. 2018 Feb;38(2):20–76.
467. Montgomery S. Fostering an environment of magnet recognition by using internet technology. Fostering an Environment of Magnet Recognition By Using Internet Technology. 2016 Jan. Minneapolis: Walden University; 2016. [dissertation]
468. Zaidi Z, Verstegen D, Naqvi R, Morahan P, Dornan T. Gender, religion, and sociopolitical issues in cross-cultural online education. Adv Health Sci Educ Theory Pract. 2016 May;21(2):287–301. PMID: 26303113 doi: 10.1007/s10459-015-9631-z
469. Keshvari M, Yamani N, Adibi P, Shahnazi H. Health Journalism: Health Reporting Status and Challenges. Iran J Nurs Midwifery Res. 2018 Feb;23(1):14–17. PMID: 29344040 doi: 10.4103/ijnmr.IJNMR_158_16
470. Abdelrahman T, Brown J, Wheat J, Thomas C, Lewis W. Hirsch Index Value and Variability Related to General Surgery in a UK Deanery. J Surg Educ. 2016 Feb;73(1):111–5. PMID: 26481425 doi: 10.1016/j.jsurg.2015.07.014
471. Hashmi A, Policherla R, Campbell H, Khan FA, Schumaier A, Al-Mufarrej F. How Informative are the Plastic Surgery Residency Websites to Prospective Applicants? J Surg Educ. 2017 Feb;74(1):74–8. PMID: 27717704 doi: 10.1016/j.jsurg.2016.08.002
472. Zhang X, Wen D, Liang J, Lei J. How the public uses social media wechat to obtain health information in china: a survey study. BMC Med Inform Decis Mak. 2017 Jul 5;17(Suppl 2):66. PMID: 28699549 doi: 10.1186/s12911-017-0470-0
473. Oladeji LO, Yu JC, Oladeji AK, Ponce BA. How Useful are Orthopedic Surgery Residency Web Pages? J Surg Educ. 2015 Dec;72(6):1185–9. PMID: 26089163 doi: 10.1016/j.jsurg.2015.05.012
474. Gaughf NW, Foster PS. Implementing a centralized institutional peer tutoring program. Educ Health (Abingdon). 2016 Aug;29(2):148–51. PMID: 27549655 doi: 10.4103/1357-6283.188773
475. Lee CI, Langlotz CP, Elmore JG. Implications of Direct Patient Online Access to Radiology Reports Through Patient Web Portals. J Am Coll Radiol. 2016 Dec;13(12 Pt B):1608–14. PMID: 27888949 doi: 10.1016/j.jacr.2016.09.007
476. Harendza S, Pyra M. Just fun or a prejudice? - physician stereotypes in common jokes and their attribution to medical specialties by undergraduate medical students. BMC Med Educ. 2017 Jul 26;17(1):128. PMID: 28747204 doi: 10.1186/s12909-017-0964-6
477. Hung S, Lai H, Chou Y. Knowledge-sharing Intention in Professional Virtual Communities: A Comparison Between Posters and Lurkers. Journal of the Association for Information Science & Technology. 2015 Dec;66(12):2494–510.
478. McGowan E, Stokes E. Leadership and leadership development within the profession of physiotherapy in Ireland. Physiother Theory Pract. 2017 Jan;33(1):62–71. PMID: 27736279 doi: 10.1080/09593985.2016.1230659
479. Foucault A, Dubé S, Fernandez N, Gagnon R, Charlin B. Learning medical professionalism with the online concordance-of-judgment learning tool (CJLT): A pilot study. Med Teach. 2015;37(10):955–60. PMID: 25336258
480. Borah BF. Longitudinal Service Learning in Medical Education: An Ethical Analysis of the Five-Year Alternative Curriculum at Stritch School of Medicine. J Med Humanit. 2018;39(4):407–16. PMID: 30079441
481. Kearney RC, Premaraj S, Smith BM, Olson GW, Williamson AE, Romanos G. Massive Open Online Courses in Dental Education: Two Viewpoints: Viewpoint 1: Massive Open Online Courses Offer Transformative Technology for Dental Education and Viewpoint 2: Massive Open Online Courses Are Not Ready for Primetime. J Dent Educ. 2016 Feb;80(2):121–7. PMID: 26834128
482. Zulkifli J, Noel B, Bennett D, O’Flynn S, O’Tuathaigh C. Medical students’ perceptions of professional misconduct: relationship with typology and year of programme. J Med Ethics. 2018 Feb;44(2):133–7. PMID: 28780525
483. Foley M, Carney T, Harris R, Fitzpatrick E, Rapca-Veillet A, Van Hout MC. Medicines containing codeine: perspectives of medical professionals in the Republic of Ireland. Ir J Med Sci. 2017 Aug;186(3):555–63. PMID: 28070817
484. Scragg B, Shaikh S, Robinson L, Mercer C. Mixed messages: An evaluation of NHS Trust Social Media policies in the North West of England. Radiography (Lond.). 2017;23(3):235–41. PMID: 28687292
485. Mather C, Cummings E, Gale F. Mobile Learning in Nursing: Tales from the Profession. Stud Health Technol Inform. 2018;252:112–7. PMID: 30040692
486. Beaumont E, Chester P, Rideout H. Navigating Ethical Challenges in Social Media: Social Work Student and Practitioner Perspectives. Australian Social Work. 2017 Mar;70(2):221–8.
487. Chiang K-F, Wang H-H. Nurses’ experiences of using a smart mobile device application to assist home care for patients with chronic disease: a qualitative study. J Clin Nurs. 2016 Jul;25(13–14):2008–17. PMID: 27136280
488. Gyllensten K, Wentz K, Håkansson C, Hagberg M, Nilsson K. Older assistant nurses’ motivation for a full or extended working life. Ageing & Society. 2018 16 July: 1-15. doi: 10.1017/S0144686X18000740
489. Thrower N, Mossman D. Online dating and personal information: Pause before you post. Current Psychiatry. 2016;15(9):88–92.
490. Craig SR, Scott R, Blackwood K. Orienting to Medicine: Scripting Professionalism, Hierarchy, and Social Difference at the Start of Medical School. Cult Med Psychiatry. 2018 Sep;42(3):654–83. PMID: 29687188
491. Crane J. Painful Times: The Emergence and Campaigning of Parents Against Injustice in 1980s Britain. 20 Century Br Hist. 2015;26(3):450–76. PMID: 26502666
492. Gershengoren L. Patient-targeted googling and psychiatric professionals. Int J Psychiatry Med. 2018 Aug 9;91217418791459. PMID: 30092677
493. Brissette MD, Johnson KA, Raciti PM, McCloskey CB, Gratzinger DA, Conran RM, et al. Perceptions of Unprofessional Attitudes and Behaviors: Implications for Faculty Role Modeling and Teaching Professionalism During Pathology Residency. Arch Pathol Lab Med. 2017 Oct;141(10):1394–401. PMID: 28686498
494. Samora JB, Lifchez SD, Blazar PE, American Society for Surgery of the Hand Ethics and Professionalism Committee. Physician-Rating Web Sites: Ethical Implications. J Hand Surg Am. 2016 Jan;41(1):104-110.e1. PMID: 26304734
495. Alexander S. Polishing Your Presence Online: Wise Use of Social Media to Enhance Your Reputation. Clin Nurse Spec 2016 Sep-Oct;30(5):261–3. PMID: 27509561
496. Badyal DK, Desai C, Tripathi SK, Dhaneria SP, Chandy SJ, Bezbaruah BK. Postgraduate pharmacology curriculum in medical institutions in India: Time for need-based appraisal and modifications. Indian J Pharmacol. 2014 Nov-Dec;46(6):584–9. PMID: 25538327
497. Baker P. Preparedness and Education in Airway Management. Anesthesiol Clin. 2015 Jun;33(2):381–95. PMID: 25999010
498. Duke P, Grosseman S, Novack DH, Rosenzweig S. Preserving third year medical students’ empathy and enhancing self-reflection using small group “virtual hangout” technology. Med Teach. 2015;37(6):566–71. PMID: 25189277
499. Cook DA, Blachman MJ, Price DW, West CP, Berger RA, Wittich CM. Professional Development Perceptions and Practices Among U.S. Physicians: A Cross-Specialty National Survey. Acad Med. 2017 Sep;92(9):1335–45. PMID: 28225460
500. Lurie Y, Mark S. Professional Ethics of Software Engineers: An Ethical Framework. Sci Eng Ethics. 2016 Apr;22(2):417–34. PMID: 26047575
501. Park SY, Park S. Professionalism and Commercialism on Cosmetic Surgeons’ Websites. Health Commun. 2017 Jun;32(7):872–9. PMID: 27419269
502. Ezeani CN, Eke HN, Ugwu F. Professionalism in library and information science. Electronic Library. 2015 Feb;33(1):2–18.
503. Ludot M, El Husseini M, Radjack R, Moro MR. Professionals’ reactions facing “radicalized” young people. An exploratory research. Neuropsychiatrie de l’Enfance et de l’Adolescence. 2017;65(3):155–63.
504. Overholser JC. Protesting the Decline While Predicting the Demise of Clinical Psychology: Can We Avoid a Total Collapse? Journal of Contemporary Psychotherapy. 2014;44(4):273–81.
505. Ali N, Fleisher W, Erickson J. Psychiatrists’ and Psychiatry Residents’ Attitudes Toward Transgender People. Acad Psychiatry. 2016 Apr;40(2):268–73. PMID: 25743203
506. Liu Y, Sun X, Qian HZ, Yin L, Yan Z, Wang L, et al. Qualitative Assessment of Barriers and Facilitators of Access to HIV Testing Among Men Who Have Sex with Men in China. AIDS Patient Care STDS. 2015 Sep;29(9):481–9. PMID: 26186029
507. Kogan LR, Hellyer PW, Stewart SM, Dowers KL. Recruitment and Hiring Strategies of Private Practitioners and Implications for Practice Management Training of Veterinary Students. J Vet Med Educ. 2015;42(2):97–106. PMID: 25804629
508. Cottey A. Reducing Ethical Hazards in Knowledge Production. Sci Eng Ethics. 2016 Apr;22(2):367–89. PMID: 25991087
509. Murray K, Stanley K. Reflections on doing, being and becoming the #OTalk student digital leader intern. In: College of Occupational Therapists 40^th^ Annual Conference and exhibition, June 28-30, 2016. British Journal of Occupational Therapy. 2016 Aug 2;79:136–7.
510. Cnossen N. Reflections on the social responsibility of a physician. South Med J. 2016 MAy;109(5):309–10. PMID: 27135729
511. Longhurst JC, Mitchell JH. Reflex control of the circulation by afferents from skeletal muscle. Int Rev Physiol. 1979;18:125–48. PMID: 361603
512. Abu-Serriah M, Dhariwal D, Martin G. Reputation of oral and maxillofacial surgery in the UK: The patients’ perspective. Br J Oral Maxillofac Surg. 2015 Apr;53(4):321–5. PMID: 25631399
513. Saxén S. Same Principles, Different Worlds: A Critical Discourse Analysis of Medical Ethics and Nursing Ethics in Finnish Professional Texts. HEC Forum. 2018 Mar;30(1):31–55. PMID: 28550382
514. Maughan ED, Johnson KH, Engelke MK. School Nursing: Becoming Credible and Visible. NASN Sch Nurse. 2017 Sep;32(5):294–7. PMID: 28823241
515. Reid L. Scientism in Medical Education and the Improvement of Medical Care: Opioids, Competencies, and Social Accountability. Health Care Anal. 2018 Jun;26(2):155–70. PMID: 28986710
516. Eijkholt M, Jankowski J, Fisher M. Screen Shots: When Patients and Families Publish Negative Health Care Narratives Online. Narrat Inq Bioeth. 2017;7(3):245–54. PMID: 29249718
517. von Gruenigen VE, Karlan BY. Sexual harassment in the work place: Its impact on gynecologic oncology and women’s health. Gynecol Oncol. 2018 May;149(2):227–9. PMID: 29526475
518. Roberts M. Should vets use gallows humour at work?. Vet Rec. 2018 Feb;182(5):146. PMID: 29419508
519. Sattar K, Roff S, Meo SA. Similarities and variances in perception of professionalism among Saudi and Egyptian Medical Students. Pak J Med Sci. 2016 Dec;32(6):1390–5. PMID: 28083032
520. Raper SE, Resnick AS, Morris JB. Simulated disclosure of a medical error by residents: development of a course in specific communication skills. J Surg Educ. 2014 Dec;71(6):e116-26. PMID: 25155639
521. Lim G, McIvor WR. Simulation-based anesthesiology education for medical students. Int Anesthesiol Clin. 2015 Fall;53(4):1–22. PMID: 26397782
522. Johnson AC, El Hajj SC, Perret JN, Caffery TS, Jones GN, Musso MW. Smartphones in Medicine: Emerging Practices in an Academic Medical Center. J Med Syst. 2015 Jan;39(1): 164. PMID: 25526706
523. Turner SR, Lai H, Bédard EL. Smoking cessation counseling by surgical and nonsurgical residents: opportunities for health promotion education. J Surg Educ. 2014 Dec;71(6):892–5. PMID: 24818539
524. Schroeder SA. Social Justice as the Moral Core of Family Medicine: A Perspective from the Keystone IV Conference. J Am Board Fam Med. 2016 Aug;29 Suppl 1:S69-71. PMID: 27387169
525. Sage M, Sage T. Social Media and E-professionalism in Child Welfare: Policy and Practice. Journal of Public Child Welfare. 2016;10(1):79–95.
526. Kedrowicz AA, Royal K, Flammer K. Social media and impression management: Veterinary Medicine students’ and faculty members’ attitudes toward the acceptability of social media posts. J Adv Med Educ Prof. 2016 Oct;4(4):155–62. PMID: 27795965
527. Boddy J, Dominelli L. Social Media and Social Work: The Challenges of a New Ethical Space. Australian Social Work. 2017 Mar;70(2):172–84.
528. Foley NM, Maher BM, Corrigan MA. Social media and tomorrow’s medical students - How do they fit? J Surg Educ. 2014 Amy-Jun;71(3):385–90. PMID: 24797855
529. Gibbons C, Richards S, Valderas JM, Campbell J. Supervised Machine Learning Algorithms Can Classify Open-Text Feedback of Doctor Performance With Human-Level Accuracy. J Med Internet Res. 2017 Mar 15;19(3):e65. PMID: 28298265
530. Walsh BC, Karia R, Egol K, Zuckerman JD, Phillips D. Teaching Professionalism in Orthopaedic Residency: Efficacy of the American Academy of Orthopaedic Surgeons Ethics Modules. J Am Acad Orthop Surg. 2018 Jul;26(14):507–14. PMID: 29847419
531. Menashe S, Otjen J, Thapa MM. Techniques for creating video content for radiology education. Radiographics. 2014 Dec;34(7):1819–23. PMID: 25384282
532. Ager A, Iacovou M. The co-construction of medical humanitarianism: analysis of personal, organizationally condoned narratives from an agency website. Soc Sci Med. 2014 Nov;120:430–8. PMID: 24915806
533. Wotherspoon SE, McCarthy PW. The factors and motivations behind United Kingdom chiropractic professional association membership: a survey of the Welsh Institute of Chiropractic Alumni. Chiropr Man Therap. 2016 Sep 12;24(1):35. PMID: 27621800.
534. Koo M, Lin S-C. The image of nursing: A glimpse of the Internet. Jpn J Nurs Sci. 2016 Oct;13(4):496–501. PMID: 27162121
535. Pianosi B, Payne H. The Professionalization of Gerontology: A Canadian Perspective. Educational Gerontology. 2014 Nov;40(11):834–46.
536. Wira CR, Francis MW, Bhat S, Ehrman R, Conner D, Siegel M. The shock index as a predictor of vasopressor use in emergency department patients with severe sepsis. West J Emerg Med. 2014;15(1):60-6. PMID: 24696751
537. Hatzipanagos S, John B, Chiu YT. The Significance of Kinship for Medical Education: Reflections on the Use of a Bespoke Social Network to Support Learners’ Professional Identities. JMIR Med Educ. 2016 Mar 3;2(1):e1. PMID: 27731848
538. Arzuaga BH, Petty C, Janvier A. The therapeutic space and doctor–parent relationship in paediatrics: trainees’ experiences and perspectives. Acta Pasediatr. 2018 nov1. PMID: 30383899
539. Agostini SD, Bonner AM. The Value of the Neurodiagnostic Professional in Patient Care. Neurodiagn J. 2015 Dec;55(4):227–34. PMID: 26793899
540. Martin L. Top 12 Psychiatric Times Blogs in 2014. Psychiatric Times. 2014 Dec;31(12):1–14.
541. John NJ, Shelton PG, Lang MC, Ingersoll J. Training Psychiatry Residents in Professionalism in the Digital World. Psychiatr Q. 2017 Jun;88(2):263–70. PMID: 27796921
542. Ho LKK, Lam AIF. Transformation of Macau policing: From a Portuguese colony to China’s SAR. Crime, Law and Social Change. 2014;61(4):417–37.
543. Shields L. Using Knowledge Wisely. Compr Child Adolesc Nurs. 2018 Apr 3;41(2):89–93. PMID: 29617164
544. Shepherd A, Sanders C, Doyle M, Shaw J. Using social media for support and feedback by mental health service users: Thematic analysis of a twitter conversation. BMC Psychiatry. 2015;15(1). PMID: 25881089
545. Harris LL, Kelley KA, Apke TL, Cleary D, Varekojis SL, White SE. Validation of an Instrument for Measuring Professional Behaviors Across Students Enrolled in Allied Health Professional Programs. J Allied Health. 2018 Fall;47(3):190–5. PMID: 30194825
546. Bezuidenhout L. Variations in Scientific Data Production: What Can We Learn from #Overlyhonestmethods? Sci Eng Ethics. 2015 Dec;21(6):1509–23. PMID: 25524321
547. Mota P, Carvalho N, Carvalho-Dias E, João Costa M, Correia-Pinto J, Lima E. Video-Based Surgical Learning: Improving Trainee Education and Preparation for Surgery. J Surg Educ. 2018 Jun;75(3):828–35. PMID: 29031522
548. Murphy GP, Awad MA, Osterberg EC, Gaither TW, Chumnarnsongkhroh T, Washington SL, et al. Web-Based Physician Ratings for California Physicians on Probation. J Med Internet Res. 2017 Aug 22;19(8):e254. PMID: 28830852
549. Joshi ART, Salami A, Hickey M, Barrett KB, Klingensmith ME, Malangoni MA. What Can SCORE Web Portal Usage Analytics Tell Us About How Surgical Residents Learn? J Surg Educ. 2017 Dec;74(6):e133–7. PMID: 29079112
550. Kaliyadan F, Ashique KT, Jagadeesan S, Krishna B. What’s up dermatology? A pilot survey of the use of WhatsApp in dermatology practice and case discussion among members of WhatsApp dermatology groups. Indian J Dermatol Venereol Leproly. 2016 Jan-Feb;82(1):67–9. PMID: 26728815
551. Schlitzkus LL, Vogt KN, Sullivan ME, Schenarts KD. Workplace bullying of general surgery residents by nurses. J Surg Educ. 2014 Dec;71(6):e149-54. PMID: 25433963
552. Lilburn LER, Breheny M, Pond R. “You’re not really a visitor, you’re just a friend”: How older volunteers navigate home visiting. Ageing and Society. 2018;38(4):817–38. doi: 10.1017/S0144686X16001380
553. Rapp AK, Healy MG, Charlton ME, Keith JN, Rosenbaum ME, Kapadia MR. YouTube is the Most Frequently Used Educational Video Source for Surgical Preparation. J Surg Educ. 2016 Dec;73(6):1072–6. PMID: 27316383
554. Caron J, Light J. “Social Media has Opened a World of ‘Open communication:’” experiences of Adults with Cerebral Palsy who use Augmentative and Alternative Communication and Social Media. Augment Altern Commun. 2016 Jan 2;32(1):25–40. PMID: 26056722
555. van Rensburg SH, Klingensmith K, McLaughlin P, Qayyum Z, van Schalkwyk GI. Patient-provider communication over social media: perspectives of adolescents with psychiatric illness. Health Expect. 2016 Feb;19(1):112–20. PMID: 25581724
556. Eghtesadi M, Florea A. Facebook, Instagram, Reddit and TikTok: a proposal for health authorities to integrate popular social media platforms in contingency planning amid a global pandemic outbreak. Can J Public Health 2020 Jun;111(3):389–91. PMID:32519085
557. Alameddine M, Tamim H, Hadid D, [et al.]. Patient attitudes toward mobile device use by health care providers in the emergency department: Cross-sectional survey. JMIR mHealth and uHealth. 2020;8(3):e16917. PMID: **32229474**
558. Asal NJ, Poyant J. Role and impact of student pharmacists and a pharmacist on an international interprofessional medical brigade. Curr Pharm Teach Learn. 2018;10(5):657–61. PMID: **29986827**
559. Azer SA, Azer S. Top-cited articles in medical professionalism: a bibliometric analysis versus altmetric scores. BMJ Open. 2019 Jul 31;9(7):e029433. PMID:31371297
560. Aziz AB, Ali SK. Relationship between level of empathy during residency training and perception of professionalism climate. BMC Med Educ. 2020 Sep 21;20(1):320. PMID:32957975
561. Backhaus J, Huth K, Entwistle A, Homayounfar K, Koenig S. Digital Affinity in Medical Students Influences Learning Outcome: A Cluster Analytical Design Comparing Vodcast With Traditional Lecture. J Surg Educ. 2019 Jun;76(3):711–9. PMID:30833205
562. Benton W, Snyder ED, Estrada CA, Bryan TJ. Signed, Sealed, Delivered: Increasing Patient Notification of Test Results in an Internal Medicine Resident Continuity Clinic. South Med J. 2019;112(2):85–8. PMID: **30708371**
563. Blau I, Shamir‐Inbal T, Hadad S. Digital collaborative learning in elementary and middle schools as a function of individualistic and collectivistic culture: The role of ICT coordinators’ leadership experience, students’ collaboration skills, and sustainability. Journal of Computer Assisted Learning 2020 Oct;36(5):672–87. doi: 10.1111/jcal.12436.
564. Bleiker J, Knapp K, Morgan-Trimmer S, Hopkins S. What Medical Imaging Professionals Talk About When They Talk About Compassion. J Med Imaging Radiat Sci. 2020 Dec;51(4S):S44–S52. PMID:32855113
565. Bly KC, Ellis SA, Ritter RJ, Kantrowitz-Gordon I. A Survey of Midwives’ Attitudes Toward Men in Midwifery. J Midwifery Womens Health. 2020 Mar;65(2):199–207. PMID:31904186
566. Boulton S, White A. Investigating non-medical prescribers’ awareness of compliance. Journal of Prescribing Practice. 2020 Sep;2(9):504–10. doi: 10.12968/jprp.2020.2.9.504
567. Campo-Arias A, Oviedo HC, Herazo E. Stigma and discrimination against transgender health professional. Revista Facultad de Medicina. 2014;62(1):41–5.
568. Chen Q. Exploring the Bottom-Up Reform of Sex Offender Registration in China: Carceral Feminism and Populist Authoritarianism. Crime, Law and Social Change. 2020;74(3):273–95. doi: 10.1007/s10611-020-09897-z
569. Choi C, Bum C-H. How do you watch sports? Differences on credibility, viewing satisfaction, flow, and reviewing intention between public TV stations and one - Person media via SNS. Sport Mont. 2019;17(1):61–7. doi: 10.26773/smj.190211
570. Damayanti FN, Absori A, Wardiono K, Rejeki S. The comparison of midwives professionalism in Indonesia and England. Journal of South India Medicolegal Association. 2020;12(1):4–9.
571. Davis L, Taylor H, Reyes H. Lifelong learning in nursing: A Delphi study. Nurse Educ Today. 2014;34(3):441–5. PMID: **23664106**
572. Douglas A, Capdeville M. From Index Medicus to the Palm of Our Hands—What’s “App-ening” in Graduate Medical Education. J Cardiothorac Vasc Anesth. 2020;34(8):2133–5. PMID: **32362541**
573. Dunne DM, Lefevre C, Cunniffe B, [et al.]. Performance Nutrition in the digital era - An exploratory study into the use of social media by sports nutritionists. J Sports Sci. 2019 Nov;37(21):2467–74. PMID:31345110
574. Fuller R, Joynes V, Cooper J, Boursicot K, Roberts T. Could COVID-19 be our “There is no alternative” (TINA) opportunity to enhance assessment? Med Teach. 2020 Jul;42(7):781–6. PMID:32552191
575. Fuse A, Navichkova Y, Alloggio K. Perception of intelligibility and qualities of non-native accented speakers. J Commun Disord. 2018;71:37–51. PMID: **29268109**
576. Garst BA, Weston KL, Bowers EP, Quinn WH. Fostering youth leader credibility: Professional, organizational, and community impacts associated with completion of an online master’s degree in youth development leadership. Children & Youth Services Review. 2019 Jan;96:1–9. doi: 10.1016/j.childyouth.2018.11.019.
577. Godlee F. Doctors should tell patients who is paying them and why. BMJ (Online). 2014;348. doi: 10.1136/bmj.g259
578. Gondal KM, Iqbal U, Ahmed A, Khan JS. Supervisors’ perspective on electronic logbook system for postgraduate medical residents of CPSP. J Coll Physicians Surg Pak. 2017;27(9):540–3. PMID: **29017667**
579. Goshtasbi K, Lehrich BM, Moshtaghi O, [et al.]. Patients’ Online Perception and Ratings of Neurotologists. Otol Neurotol. 2019;40(1):139–43. PMID: **30531643**
580. Gressel GM, Lundsberg LS, Illuzzi JL, [et al.]. Patient and provider perspectives on Bedsider.org, an online contraceptive information tool, in a low income, racially diverse clinic population. Contraception. 2014;90(6):588–93. PMID: **25139723**
581. Grol SM, Schers HJ. The perspective of frail elderly on their care networks in primary care. International Journal of Integrated Care (IJIC). 2016 Dec 2;16(6):1–2. doi: [10.5334/ijic.3011](http://doi.org/10.5334/ijic.3011)
582. Head B, Peters B, Middleton A, Friedman C, Guman N. Results of a nationwide hospice and palliative care social work job analysis. J Soc Work End Life Palliat Care. 2019 Jan;15(1):16–33. PMID: **30892137**
583. Hoffman SJ, Silverberg SL. Training the next generation of global health advocates through experiential education: A mixed-methods case study evaluation. Can J Public Health. 2015;106(6):e442–9. PMID: **26680437**
584. Huber SA, Priestley J, Kasabwala K, Gadidov B, Culligan P. Understanding Your Online Ratings: A Methodological Analysis Using Urogynecologists in the United States. Female Pelvic Med Reconstr Surg. 2019;25(2):193–7. PMID: **30807427**
585. Jackel B. A Survey of Parents of Children With Cortical or Cerebral Visual Impairment: 2018 Follow-up. Semin Pediatr Neurol. 2019;31:3–4. PMID: **31548021**
586. Jagodzinski A, Johansen C, Koch-Gromus U, [et al.]. Rationale and Design of the Hamburg City Health Study. Eur J Epidemiol. 2020;35(2):169–81. PMID: **31705407**
587. Jiménez-Marín G, Zambrano RE, Galiano-Coronil A, Ravina-Ripoll R. Food and Beverage Advertising Aimed at Spanish Children Issued through Mobile Devices: A Study from a Social Marketing and Happiness Management Perspective. Int J Environ Res Public Health. 2020;17(14):1–18. PMID: **32674347**
588. Ju C, Zhang S. Influencing Factors of Continuous Use of Web-Based Diagnosis and Treatment by Patients With Diabetes: Model Development and Data Analysis. J Med Internet Res. 2020 Sep 28;22(9):e18737. PMID:32771982
589. Knox ADC, Reddy S, Mema B, Demoya M, Cilli-Turner Ma E, Harris I. Back in the day. What are surgeon bloggers saying about their careers?. J Surg Educ. 2014;71(1):21–31. PMID: **24411419**
590. Labrague LJ, McEnroe-Petitte D, D’Souza MS, [et al.]. Capability beliefs and the intention to adopt evidence-based practices in the future among nursing students: An international study. J Prof Nurs. 2020 Sep;36(5):301–7. PMID: **33039062**
591. Leach MJ, Shaw R, Austin P, [et al.]. Attitudes, skills, and use of evidence-based practice: A cross-sectional survey of Swedish osteopaths. International Journal of Osteopathic Medicine. 2020 Dec;38:41–9. doi: 10.1016/j.ijosm.2020.10.006
592. Malik FS, Panlasigui N, Gritton J, Gill H, Yi-Frazier JP, Moreno MA. Adolescent Perspectives on the Use of Social Media to Support Type 1 Diabetes Management: Focus Group Study. J Med Internet Res. 2019 May 30;21(6):e12149. PMID:31199310
593. Martin-Kerry JM, Knapp P, Atkin K, [et al.]. Supporting children and young people when making decisions about joining clinical trials: Qualitative study to inform multimedia website development. BMJ Open. 2019;9(1). doi: 10.1136/bmjopen-2018-023984_rfseq1
594. Misra V, Chemane N, Maddocks S, Chetty V. Community-based primary healthcare training for physiotherapy: Students’ perceptions of a learning platform. S Afr J Physiother. 2019;75(1):471. PMID:31206093
595. Moss E, Bergren MD, Maughan ED. School Nurse Websites: What Do They Tell Us About School Nurses? J Sch Nurs. 2019 Dec;35(6):395–400. PMID: **30983512**
596. Mullikin TC, Shahi V, Grbic D, Pawlina W, Hafferty FW. First Year Medical Student Peer Nominations of Professionalism: A Methodological Detective Story about Making Sense of Non-Sense. Anat Sci Educ. 2019;12(1):20–31. PMID: **29569347**
597. Navabi N, Okhovati M, Alsadat Hashemipour M. Can Internet Anxiety Affect Electronic Journals Usage? A CrossSectional Study with Iranian Postgraduate Dental Students. Pesquisa Brasileira em Odontopediatria e Clinica Integrada. 2019;19(1):e4559. doi: 10.4034/pboci.2019.191.57
598. Ogbogu U, Du J, Koukio Y. The involvement of Canadian physicians in promoting and providing unproven and unapproved stem cell interventions. BMC Medical Ethics. 2018;19(1):32. PMID: **29716594**
599. Okike K, Berger PZ, Schoonover C, O′Toole RV. Do Orthopaedic Resident and Fellow Case Logs Accurately Reflect Surgical Case Volume?. J Surg Educ. 2018;75(4):1052–7. PMID: **29287752**
600. Page TM, Lloyd HM, Williamson GR, Jones RB. Can Nursing Students Gain Person-Centred Skills while Supporting Citizen Contacts to use the Internet for Health? International Journal of Integrated Care (IJIC). 2018 Oct 2;18:1–2. doi: 10.5334/ijic.s2292
601. Persky AM, Kirwin JL, Marasco CJ, May DB, Skomo ML, Kennedy KB. Classroom attendance: Factors and perceptions of students and faculty in US schools of pharmacy. Currents in Pharmacy Teaching and Learning. 2014;6(1):1–9. doi:10.1016/j.cptl.2013.09.014
602. Pettersen H, Landheim A, Skeie I, [et al.]. How Social Relationships Influence Substance Use Disorder Recovery: A Collaborative Narrative Study. Subst Abuse. 2019;13:1178221819833379. PMID:30886519
603. Poowuttikul P, Seth D. New Concepts and Technological Resources in Patient Education and Asthma Self-Management. Clin Rev Allergy Immunol. 2020 Aug;59(1):19–37. PMID:32215784
604. Reyes AN, Brown CA. Factors affecting occupational therapists’ decision to join their regional professional association: Facteurs influençant la décision des ergothérapeutes de joindre les rangs de leur association professionnelle régionale. Can J Occup Ther. 2018;85(3):222–31. PMID: **29649888**
605. Ristkari T, Kurki M, Suominen A, [et al.]. Web-based parent training intervention with telephone coaching for disruptive behavior in 4-year-old children in real-world practice: Implementation study. J Med Int Res. 2019;21(4):e114446. PMID: **30973337**
606. Sellberg M, Skavberg Roaldsen K, Nygren-Bonnier M, Halvarsson A. Clinical supervisors’ experience of giving feedback to students during clinical integrated learning. Physiother Theory Pract. 2020;1-10. PMID: **32149555**
607. Shorey S, Goh ML, Ang SY, Ang L, Devi MK, Ang E. The Progression and Future of Nursing in Singapore: A Descriptive Qualitative Study. J Transcult Nurs. 2019 Sep;30(5):512–20. PMID: **30688168**
608. Singh A, Ravi P, Lepcha K. Patient satisfaction factors with in house Third Party Administrator (TPA) department of a tertiary care hospital: a cross sectional analysis. International Journal of Healthcare Management. 2021;14: 603-609. doi: 10.1080/20479700.2019.1679519
609. Somerson JS, Patton A, Ahmed AA, Ramey S, Holliday EB. Burnout Among United States Orthopaedic Surgery Residents. J Surg Educ 2020. Aug;77(4):961–8. PMID:32171748
610. Sunell S, Laronde DM, Kanji Z. Dental hygiene graduates’ educational preparedness: Self‐confidence ratings of the CDHA baccalaureate competencies. International Journal of Dental Hygiene. 2020 Aug;18(3):295–306. doi: 10.1111/idh.12434
611. Tan Y, Teng Z, Qiu Y, Tang H, Xiang H, Chen J. Potential of Mobile Technology to Relieve the Urgent Mental Health Needs in China: Web-Based Survey. JMIR Mhealth Uhealt.h 2020 Jul 7;8(7):e16215. PMID:32673239
612. Tâncu AMC, Purcărea VL, Pantea M, Imre M. Key Factors Correlations in Selecting Dental Services. J Med Life 2019 Mar;12(1):83–9. PMID:31123530
613. Thanawala RM, Jesneck JL, Seymour NE. Education Management Platform Enables Delivery and Comparison of Multiple Evaluation Types. J Surg Educ. 2019;76(6):e209–16. PMID: **31515199**
614. Tinner LE, Kaner E, Garnett C, [et al.]. Qualitative Evaluation of Web-Based Digital Intervention to Prevent and Reduce Excessive Alcohol Use and Harm Among Young People Aged 14-15 Years: A “Think-Aloud” Study. JMIR Pediatr Parent. 2020 Dec 15;3(2):e19749. PMID:33320100
615. Trappenburg M, van Beek G. “My profession is gone”: how social workers experience de-professionalization in the Netherlands. European Journal of Social Work. 2019 Jul;22(4):676–89. doi: 10.1080/13691457.2017.1399255
616. Williams AD, Greenwald EE, Soricelli RL, DePace DM. Medical students’ reactions to anatomic dissection and the phenomenon of cadaver naming. Anat Sci Educ. 2014;7(3):169–80. PMID: **23913911**
617. Wu XV, Chi Y, Selvam UP, [et al.]. A Clinical Teaching Blended Learning Program to Enhance Registered Nurse Preceptors’ Teaching Competencies: Pretest and Posttest Study. J Med Internet Res. 2020;22(4):e18604. PMID: **32329743**
618. Yeh Y-T, Chen H-Y, Cheng K-J, Hou S-A, Yen Y-H, Liu C-T. Evaluating an online pharmaceutical education system for pharmacy interns in critical care settings. Comput Methods Programs Biomed. 2014;113(2):682–9. PMID: **24315478**
619. Zeijlemaker C, Moosa S. The prevalence of burnout among registrars in the School of Clinical Medicine at the University of the Witwatersrand, Johannesburg, South Africa. S Afr Med J. 2019 Aug 28;109(9):668–72. PMID:31635592
620. Zhang R, Lu X, Wu W, Shang X. Why do patients follow physicians’ advice? The influence of patients’ regulatory focus on adherence: an empirical study in China. BMC Health Serv Res. 2019 May 10;19(1):301. PMID:31077196
621. Zhang Z, Yang H, He J, Lu X, Zhang R. The Impact of Treatment-Related Internet Health Information Seeking on Patient Compliance. Telemed J E Health. 2020 Sep 9; PMID:32907505
622. Zhu X, Hu H, Xiong Z, [et al.]. Utilization and professionalism toward social media among undergraduate nursing students. Nurs Ethics. 2020 Sep 25;096973302095210. PMID: **32975494**
623. Zigdon A, Zigdon T, Moran DS. Attitudes of Nurses Towards Searching Online for Medical Information for Personal Health Needs: Cross-Sectional Questionnaire Study. J Med Internet Res. 2020 Mar 16;22(3):e16133. PMID:32175910
624. Chisholm-Burns MA, Spivey CA, Jaeger MC, Williams J, George C. Development of an Instrument to Measure Pharmacy Student Attitudes Toward Social Media Professionalism. Am J Pharm Educ. 2017 May;81(4):65. PMID: 28630506
625. Willemse JJ, Bozalek V. Exploration of the affordances of mobile devices in integrating theory and clinical practice in an undergraduate nursing programme. Curationis. 2015;38(2):e1–e10. PMID: 26852423
626. Jones R, Kelsey J, Nelmes P, Chinn N, Chinn T, Proctor-Childs T. Introducing Twitter as an assessed component of the undergraduate nursing curriculum: case study. J Adv Nurs. 2016 Jul;72(7):1638–53. PMID: 26861572
627. Chester AN, Walthert SE, Gallagher SJ, Anderson LC, Stitely ML. Patient-targeted Googling and social media: a cross-sectional study of senior medical students. BMC Med Ethics. 2017 Dec 4;18(1):70. PMID: 29202840
628. Whiting M, Kinnison T, Mossop L. Teaching Tip: Developing an Intercollegiate Twitter Forum to Improve Student Exam Study and Digital Professionalism. J Vet Med Educ. 2016 Fall;43(3):282–6. PMID: 26966982
629. Jackson J, Gettings S, Metcalfe A. “The power of Twitter”: Using social media at a conference with nursing students. Nurse Educ Today. 2018 Sep;68:188–91. PMID: 29945099
630. Twynstra J, Dworatzek P. Use of an Experiential Learning Assignment to Prepare Future Health Professionals to Utilize Social Media for Nutrition Communications. Can J Diet Pract Res. 2016 Mar;77(1):30–4. PMID: 26451805
631. Armitage-Chan E, Maddison J, May SA. What is the veterinary professional identity? Preliminary findings from web-based continuing professional development in veterinary professionalism. Vet Rec. 2016 Mar 26;178(13):318. PMID: 26857071
632. Dressler JA, Ryder BA, Connolly M, Blais MD, Miner TJ, Harrington DT. “Tweet”-Format Writing Is an Effective Tool for Medical Student Reflection. J Surg Educ. 2018;75(5):1206–10. PMID: **29576247**
633. Feizy F, Sadeghian E, Shamsaei F, Tapak L. The relationship between internet addiction and psychosomatic disorders in Iranian undergraduate nursing students: a cross-sectional study. J Addict Dis. 2020;38(2):164–9. PMID: **32469289**
634. Geyer E, Irish E, Hagzan A, Wiczulis A. Social Media and Online Professionalism Integrated into Year 3 OB/GYN Clerkship. Med Ref Serv Q. 2020 Dec;39(4):359–69. PMID:33085946
635. Heinonen A-T, Kääriäinen M, Juntunen J, Mikkonen K. Nursing students’ experiences of nurse teacher mentoring and beneficial digital technologies in a clinical practice setting. Nurse Educ Pract. 2019 Oct;40:102631. PMID: **31585310**
636. Jalil A, Mahmood QK, Fischer F. Young medical doctors’ perspectives on professionalism: a qualitative study conducted in public hospitals in Pakistan. BMC Health Serv Res. 2020 Sep 10;20(1):847. PMID:32912271
637. McGurgan P, Calvert KL, Narula K, Celenza A, Nathan EA, Jorm C. Medical students’ opinions on professional behaviours: The Professionalism of Medical Students’ (PoMS) study. Med Teach. 2020 Mar;42(3):340–50. PMID: **31738619**
638. O’Doherty D, Lougheed J, Hannigan A, [et al.]. Internet skills of medical faculty and students: is there a difference? BMC Med Educ. 2019 Jan 30;19(1):39. PMID:30700293
639. Sharma N, Advani U, Sharma L, Jain M, Sharma K, Dixit A. Pattern of mobile phone usage among medical students. International Journal of Academic Medicine. 2019;5(2):118–23. doi: 10.4103/IJAM.IJAM_61_18
640. Xu Y, Francis Z, Saleem K, [ et al.]. Usage of smart devices amongst medical practitioners in universitas academic hospital. S Afr Fam Pract. 2020;62(1):e1-e7. PMID: **32148052**
641. Fatollahi JJ, Colbert JA, Agarwal P, [et al.]. The Impact of Physician Social Media Behavior on Patient Trust. AJOB Empir Bioeth. 2020 Jun;11(2):77–82. PMID:31663810
642. Lai D, Wang D, Calvano J, Raja AS, He S. Addressing immediate public coronavirus (COVID-19) concerns through social media: Utilizing Reddit’s AMA as a framework for Public Engagement with Science. PLoS One. 2020;15(10):e0240326. PMID:33021985
643. Ryan G, Jackson J, Cornock M. Exploring public perspectives of e-professionalism in nursing. Nurs Manag (Harrow). 2019 Dec 2;26(6):29–35. PMID:31686468
644. Weijs C, Coe J, Desmarais S, Majowicz S, Jones-Bitton A. Effects of Mock Facebook Workday Comments on Public Perception of Professional Credibility: A Field Study in Canada. J Med Internet Res. 2019 Apr 18;21(4):e12024. PMID:30998223
645. Kalia V, Patel AK, Moriarity AK, Canon CL. Personal Branding: A Primer for Radiology Trainees and Radiologists. J Am Coll Radiol. 2017 Jul;14(7):971–5. PMID: 28476605
646. Baron RJ, Berinsky AJ. Mistrust in science - A threat to the patient-physician relationship. N Engl J Med. 2019;381(2):182–5. PMID: **31291524**
647. Daigle A. Social media and professional boundaries in undergraduate nursing students. J Prof Nurs. 2020 Mar;36(2):20–3. PMID: **32204855**
648. What does coronavirus mean for community nurses? Journal of Community Nursing. 2020 Apr;34(2):8–13.
649. Brazil V, Parker C. A day in the life: social media for clinical practice and medical education. Med J Aust. 2017 Jun 19;206(11):478–80. PMID: 28918726
650. Hudson S, French A. CardioTweeters: an analysis of Twitter use by UK cardiologists. British Journal of Cardiology. 2018 Jul;25(3):1–5.
651. Gill BC, Zhang JJH, Knoedler MA, Shoskes DA, Vasavada SP. Digital Identity in Academic Urology: Assessment of Female Pelvic Medicine and Reconstructive Surgery, and Opportunity for Improvement. Urology Practice. 2017;4(5):425–9.
652. Curran V, Fleet L, Simmons K, Ravalia M, Snow P. Exploratory Study of Rural Physicians’ Self-Directed Learning Experiences in a Digital Age. J Contin Educ Health Prof. 2016;36(4):284–9. PMID: 28350310
653. Booth RG. Happiness, stress, a bit of vulgarity, and lots of discursive conversation: a pilot study examining nursing students’ tweets about nursing education posted to Twitter. Nurse Educ Today. 2015 Feb;35(2):322–7. PMID: 25467718
654. Augustine JM, Jackowski McKinley RM, Warholak TL, Yehoshua A, Ip Q, Armstrong EP. Perceptions of student pharmacists on professionalism and social networking sites: A Rasch analysis. Currents in Pharmacy Teaching and Learning. 2015;7(5):645–55.
655. Call T, Hillock R. Professionalism, social media, and the Orthopaedic Surgeon: What do you have on the Internet? Technol Health Care. 2017;25(3):531–9. PMID: 28128772
656. Kogan LR, Hellyer PW, Stewart SM, Hendrickson DA, Dowers KL, Schoenfeld-Tacher R. Researching Applicants Online in the Veterinary Program Admissions Process: Perceptions, Practices, and Implications for Curricular Change. J Vet Med Educ. 2015;42(4):286–96. PMID: 26291414
657. Probst YC, Peng Q. Social media in dietetics: Insights into use and user networks. Nutr Diet. 2018 Oct 28. PMID: 30370651. doi: 10.1111/1747-0080.12488.
658. Social media still a work in progress for physicians. Physician Practice Perspectives. 2016 Feb;35(2):1–5.
659. Smith GC, Knudson TK. Student nurses’ unethical behavior, social media, and year of birth. Nurs Ethics. 2016 Dec;23(8):910–8. PMID: 26169881
660. Bramstedt KA, Ierna BN, Woodcroft-Brown VK. Using SurveyMonkey® to teach safe social media strategies to medical students in their clinical years. Communication and Medicine. 2014;11(2):117–24.
661. Gagnon K. Using Twitter in Health Professional Education. J Allied Health. 2015 Spring;44(1):25–33. PMID: 25743398
662. Bacaksiz FE, Eskici GT, Seren AKH. “From my Facebook profile”: What do nursing students share on Timeline, Photos, Friends, and About sections? Nurse Educ Today. 2020 Mar;86:104326. PMID:31945672
663. De Clercq E, Rost M, von der Weid N, Ansari M, Elger BS. To be or not to be in the social media arena? The perspective of healthcare providers working within adolescent and young adult oncology in Switzerland. Int J Adolesc Med Health. 2020 Aug 27; PMID:32860667
664. Dungarwalla M, Chapireau D, Bentley R. Use of WhatsApp in an oral and maxillofacial surgery department at a major trauma centre and its role during major incidents: our experience. Br J Oral Maxillofac Surg. 2019;57(5):449–53. PMID: **31006506**
665. Guo J-W, Tay DL, Litchman ML. Hashtags and heroes: perceptions of nursing on Twitter following a high profile nurse arrest. J Prof Nurs. 2019 Oct;35(5):398–404. PMID:31519344
666. Holden ACL, Adam L, Thomson WM. Dentists’ Perspectives on Commercial Practices in Private Dentistry. JDR Clin Trans Res. 2020 Dec 7;2380084420975700. PMID:33283607
667. Jones S, Chudleigh M, Baines R, Jones RB. Did introducing Twitter and digital professionalism as an assessed element of the nursing curriculum impact social media related incidence of “Fitness to Practise”: 12-year case review. Nurse Educ Pract. 2020 Dec 5;50:102950. PMID:33310508
668. Mulaikal TA, Helou MF, Martinelli SM. Training the next generation of anesthesiologists. Int Anesthesiol Clin. 2020;58(4):23–30. PMID: **32852315**
669. Omanga AO, Mua BN, Edalia LG, Gakonyo J. Adherence to professionalism and ethical practice on social media among dentists in Nairobi. East African Medical Journal. 2019;96(5):2700–8.
670. Shah R, Dyke A, Harris L, Hodges S. How accessible are you? A hospital-wide audit of the accessibility and professionalism of Facebook profiles. Br Dent J. 2019 Jun;226(11):878–82. PMID: **31203342**
671. Wagner JP, Cochran AL, Jones C, Gusani NJ, Varghese TK, Attai DJ. Professional Use of Social Media Among Surgeons: Results of a Multi-Institutional Study. J Surg Educ. 2018 Jun;75(3):804–10. PMID:28964746
