# Supplementary material for: Dangers and Benefits of Social Media on E-Professionalism of Health Care Professionals: Scoping Review
Source: J Med Internet Res. 2021 Nov 17;23(11):e25770. doi: 10.2196/25770 (PMC8663533; doi:10.2196/25770)
Supplement: Multimedia Appendix 4 [file jmir_v23i11e25770_app4.docx]

**Multimedia Appendix 4.** Quality of the reviewed studies.

Quality of qualitative and mixed-methods studies [n=36]

Critical Appraisal Skills Programme (CASP) Qualitative Research Checklist [1].

1. Was there a clear statement of the aims of the research?
2. Is a qualitative methodology appropriate?
3. Was the research design appropriate to address the aims of the research?
4. Was the recruitment strategy appropriate to the aims of the research?
5. Was the data collected in a way that addressed the research issue?
6. Has the relationship between researcher and participants been adequately considered?
7. Have ethical issues been taken into consideration?
8. Was the data analysis sufficiently rigorous?
9. Is there a clear statement of findings?
10. How valuable is the research?

| **Year** | **First author** | **(1)** | **(2)** | **(3)** | **(4)** | **(5)** | **(6)** | **(7)** | **(8)** | **(9)** | **(10)** | **Rating** |
| --- | --- | --- | --- | --- | --- | --- | --- | --- | --- | --- | --- | --- |
| **2014** | George et al [2] | YES | YES | YES | YES | YES | YES | YES | YES | YES | YES | SATISFACTORY |
|  | Henry et al [3] | YES | YES | YES | YES | YES | YES | YES | YES | YES | YES | SATISFACTORY |
|  | Langenfeld et al [4] | YES | YES | YES | YES | YES | YES | YES | YES | YES | YES | SATISFACTORY |
|  | Ness et al [5] | YES | YES | YES | YES | YES | YES | YES | YES | YES | YES | SATISFACTORY |
| **2015** | Chretien et al [6] | YES | YES | YES | YES | YES | YES | YES | YES | YES | YES | SATISFACTORY |
|  | Jawaid et al [7] | YES | YES | YES | YES | YES | YES | NA | YES | YES | YES | SATISFACTORY |
|  | Flickinger et al [8] | YES | YES | YES | YES | YES | NA | YES | NA | YES | YES | SATISFACTORY |
|  | Langenfeld et al [9] | YES | YES | YES | YES | YES | NA | YES | YES | YES | YES | SATISFACTORY |
|  | Khandelwal et al [10] | YES | YES | YES | YES | YES | YES | YES | YES | YES | YES | SATISFACTORY |
|  | Marnocha et al [11] | YES | YES | YES | YES | YES | NA | YES | YES | YES | YES | SATISFACTORY |
|  | Mawdsley [12] | YES | YES | YES | YES | YES | NA | YES | YES | YES | YES | SATISFACTORY |
|  | Nyageni et al [13] | YES | YES | YES | YES | YES | YES | YES | YES | YES | YES | SATISFACTORY |
|  | Pereira et al [14] | YES | YES | YES | YES | YES | YES | NA | YES | YES | YES | SATISFACTORY |
| **2016** | Campbell et al [15] | YES | YES | YES | YES | YES | YES | YES | YES | YES | YES | SATISFACTORY |
|  | Ferguson et al [16] | YES | YES | YES | YES | YES | NA | YES | YES | YES | YES | SATISFACTORY |
|  | Panahi et al [17] | YES | YES | YES | YES | YES | YES | YES | YES | YES | YES | SATISFACTORY |
| **2017** | Benetoli et al [18] | YES | YES | YES | YES | YES | YES | YES | YES | YES | YES | SATISFACTORY |
|  | Chester et al [19] | YES | YES | YES | YES | YES | YES | YES | YES | YES | YES | SATISFACTORY |
|  | Duke et al [20] | YES | YES | YES | YES | YES | YES | YES | YES | YES | YES | SATISFACTORY |
|  | Gomes et al [21] | YES | YES | YES | YES | YES | YES | NA | YES | YES | YES | SATISFACTORY |
|  | Henning et al [22] | YES | YES | YES | YES | YES | YES | YES | YES | YES | YES | SATISFACTORY |
|  | Jafarey et al [23] | YES | YES | YES | YES | YES | NA | YES | YES | YES | YES | SATISFACTORY |
|  | Koo et al [24] | YES | YES | YES | YES | YES | YES | YES | YES | YES | YES | SATISFACTORY |
|  | Neville et al [25] | YES | YES | YES | YES | YES | YES | YES | YES | YES | YES | SATISFACTORY |
|  | Nicolai et al [26] | YES | YES | YES | YES | YES | YES | YES | YES | YES | YES | SATISFACTORY |
|  | Scragg et al [27] | YES | YES | YES | YES | YES | YES | YES | YES | YES | YES | SATISFACTORY |
|  | Soares et al [28] | YES | YES | YES | YES | YES | YES | YES | YES | YES | YES | SATISFACTORY |
| **2018** | Knott et al [29] | YES | YES | NO | NO | YES | NO | YES | NO | NO | CD | SATISFACTORY |
|  | Koo et at [30] | YES | YES | YES | YES | YES | YES | YES | YES | YES | YES | SATISFACTORY |
|  | Nason et al [31] | YES | YES | YES | YES | YES | YES | YES | YES | YES | YES | SATISFACTORY |
| **2019** | Al Qarni et al [32] | YES | YES | YES | YES | YES | YES | YES | YES | YES | YES | SATISFACTORY |
|  | Hsieh et al [33] | YES | YES | YES | YES | YES | YES | YES | YES | YES | YES | SATISFACTORY |
|  | Justinia et al [34] | YES | YES | YES | YES | YES | YES | YES | YES | YES | YES | SATISFACTORY |
| **2020** | Loo et al [35] | YES | YES | YES | YES | YES | YES | YES | YES | YES | YES | SATISFACTORY |
|  | Kerr et al [36] | YES | YES | YES | YES | YES | NA | YES | YES | YES | YES | SATISFACTORY |
|  | Ruan et al [37] | YES | YES | YES | YES | YES | YES | YES | YES | YES | YES | SATISFACTORY |

Quality Assessment Tool for Observational Cohort and Cross-Sectional Studies [n=52]

National Heart, Lung, and Blood Institute. 2014. Quality Assessment Tool for Observational Cohort and Cross-Sectional Studies - NHLBI, NIH [38].

1. Was the research question or objective in this paper clearly stated?
2. Was the study population clearly specified and defined?
3. Was the participation rate of eligible persons at least 50%?
4. Were all the subjects selected or recruited from the same or similar populations (including the same time period)? Were inclusion and exclusion criteria for being in the study prespecified and applied uniformly to all participants?
5. Was a sample size justification, power description, or variance and effect estimates provided?
6. For the analyses in this paper, were the exposure(s) of interest measured prior to the outcome(s) being measured?
7. Was the timeframe sufficient so that one could reasonably expect to see an association between exposure and outcome if it existed?
8. For exposures that can vary in amount or level, did the study examine different levels of the exposure as related to the outcome (e.g., categories of exposure, or exposure measured as continuous variable)?
9. Were the exposure measures (independent variables) clearly defined, valid, reliable, and implemented consistently across all study participants?
10. Was the exposure(s) assessed more than once over time?
11. Were the outcome measures (dependent variables) clearly defined, valid, reliable, and implemented consistently across all study participants?
12. Were the outcome assessors blinded to the exposure status of participants?
13. Was loss to follow-up after baseline 20% or less?
14. Were key potential confounding variables measured and adjusted statistically for their impact on the relationship between exposure(s) and outcome(s)?

| **Year** | **First author** | **(1)** | **(2)** | **(3)** | **(4)** | **(5)** | **(6)** | **(7)** | **(8)** | **(9)** | **(10)** | **(11)** | **(12)** | **(13)** | **(14)** | **Rating** |
| --- | --- | --- | --- | --- | --- | --- | --- | --- | --- | --- | --- | --- | --- | --- | --- | --- |
| **2014** | Bagley et al [39] | YES | YES | NO | YES | NO | NO | NA | YES | YES | NO | YES | NA | NA | NA | FAIR |
|  | Levati et al [40] | YES | YES | NO | YES | NO | NO | NA | NA | YES | NO | YES | NA | NA | NA | GOOD |
|  | Rocha et al [41] | YES | YES | YES | YES | NO | NO | NA | YES | YES | NO | YES | NA | NA | NA | GOOD |
| **2015** | Alkhateeb et al [42] | YES | YES | YES | YES | NO | NO | NA | YES | YES | NO | YES | NA | NA | NA | GOOD |
|  | Avci et al [43] | YES | YES | YES | YES | YES | NO | NA | NA | YES | NO | YES | NA | NA | NA | GOOD |
|  | Barlow et al [44] | YES | YES | YES | YES | NO | NO | NA | YES | YES | NO | YES | NA | NA | NA | GOOD |
|  | Brisson et al [45] | YES | YES | NO | YES | NO | NO | NA | YES | YES | NO | YES | NA | NA | NA | GOOD |
|  | Borgmann et al [46] | YES | YES | NO | YES | YES | NO | NO | NA | YES | NO | YES | NA | NO | NA | GOOD |
|  | Gupta et al [47] | YES | YES | YES | YES | NO | NO | NA | NA | YES | NO | YES | NA | NA | NA | GOOD |
|  | Fuoco et al [48] | YES | YES | NO | YES | NO | NO | NA | NA | YES | NO | YES | NA | NA | NA | GOOD |
|  | Klee et al [49] | YES | YES | NO | YES | NO | NO | NA | YES | YES | NO | YES | YES | NA | NA | GOOD |
|  | Mohiuddin et al [50] | YES | YES | YES | YES | CD | YES | YES | NO | NO | NO | YES | YES | NA | NA | GOOD |
|  | Walton et al [51] | YES | YES | NR | YES | NO | YES | YES | YES | YES | YES | YES | NA | YES | NA | GOOD |
| **2016** | Adilman et al [52] | YES | YES | NO | YES | NO | NO | NA | YES | YES | YES | YES | NA | NA | NA | GOOD |
|  | Garg et al [53] | YES | YES | NO | YES | NO | NO | NA | YES | YES | NO | YES | NA | NA | NA | GOOD |
|  | Getting et al [54] | YES | YES | YES | YES | YES | NO | NA | NA | YES | NO | YES | NA | NA | NA | GOOD |
|  | Kenny et al [55] | YES | YES | YES | YES | NO | NO | NA | YES | YES | NO | YES | NA | NA | NA | GOOD |
|  | Kesselheim et al [56] | YES | YES | YES | YES | NO | NO | NA | NA | YES | NO | YES | NA | NA | NA | GOOD |
|  | Kitsis et al [57] | YES | YES | YES | YES | YES | NO | YES | NA | YES | NO | YES | NA | NA | NA | GOOD |
|  | Langenfeld [58] | YES | YES | NO | YES | NO | NO | NA | NA | YES | NO | YES | NA | NA | NA | GOOD |
|  | Lefebvre et al [59] | YES | YES | YES | YES | NO | NO | NA | YES | YES | NO | NA | NA | NA | NA | GOOD |
|  | Mather et al [60] | YES | NO | NO | YES | NO | NO | NA | YES | YES | NO | YES | NA | NA | NA | FAIR |
|  | Nikiphorou et al [61] | YES | YES | NO | YES | NO | NO | NA | NA | YES | NO | YES | NA | NA | NA | GOOD |
|  | Robertson et al [62] | YES | YES | YES | YES | NR | NA | YES | YES | YES | NA | YES | NA | NA | NA | GOOD |
|  | Yang et al [63] | YES | YES | YES | YES | YES | NO | NA | NA | YES | NO | YES | NA | NA | NA | GOOD |
| **2017** | Dawkins et al [64] | YES | YES | NO | YES | YES | NO | NA | NA | YES | NO | YES | NA | NA | NA | GOOD |
|  | D’Souza et al [65] | YES | YES | NO | YES | YES | NO | NA | NA | YES | NO | YES | NA | NA | NA | GOOD |
|  | Duyumus et al [66] | YES | YES | NO | YES | YES | YES | NA | NA | YES | NO | YES | NA | NA | NA | GOOD |
|  | Laliberté et al [67] | YES | YES | NO | YES | NO | NO | NA | NA | YES | NO | YES | NA | NA | NA | GOOD |
|  | Long et al [68] | YES | YES | YES | YES | NO | YES | YES | NO | YES | NO | YES | NA | NA | NA | GOOD |
|  | Marnocha et al [69] | YES | YES | YES | YES | NO | YES | NA | YES | YES | YES | YES | NA | NA | YES | GOOD |
|  | Mostaghimi et al [70] | YES | YES | YES | YES | NO | NO | NA | YES | YES | YES | YES | NA | NO | NA | GOOD |
|  | O’Sullivan et al [71] | YES | YES | NO | YES | NO | NO | NA | YES | YES | NO | YES | NA | NA | NA | GOOD |
|  | Patel et al [72] | YES | YES | YES | YES | NO | NO | NA | NA | YES | NO | YES | NA | NA | NA | GOOD |
|  | Surani et al [73] | YES | YES | YES | YES | YES | NO | NA | NA | NO | NO | NA | NA | NA | NA | GOOD |
|  | Wagner et al [74] | YES | YES | NO | YES | NO | NO | NA | NA | YES | NO | YES | NA | NA | NA | GOOD |
|  | Weijs et al [75] | YES | YES | NR | YES | NO | NO | NA | YES | YES | NO | YES | NA | NA | NA | GOOD |
| **2018** | Alshakhs et al [76] | YES | YES | NR | YES | NO | NO | NA | YES | YES | NO | NA | NA | NA | NA | GOOD |
|  | Barnable et al [77] | YES | YES | NO | YES | NO | NO | NA | YES | YES | NO | YES | NA | NA | NA | GOOD |
|  | Hazzam et al [78] | YES | YES | NO | YES | YES | YES | NA | NA | YES | NO | YES | NA | NA | NA | GOOD |
|  | Hinojo-Lucena et al [79] | YES | YES | NR | YES | NO | NO | NA | NA | YES | NO | YES | NA | NA | NA | FAIR |
|  | Irfan et al [80] | YES | YES | YES | YES | YES | NO | NA | NA | YES | NO | YES | NA | NA | NA | GOOD |
|  | West et al [81] | YES | YES | NO | YES | NO | NO | NA | NA | YES | NO | YES | NA | NA | NA | GOOD |
| **2019** | Dobson et al [82] | YES | YES | YES | YES | NO | NO | NA | NA | YES | NO | YES | NA | NA | NA | GOOD |
|  | Lee et al [83] | YES | YES | YES | YES | NR | NA | NA | NA | YES | NA | YES | NA | NA | NA | GOOD |
|  | Renew et al [84] | YES | YES | NO | YES | NR | NA | NA | NA | YES | NA | YES | NA | NA | NA | GOOD |
|  | Sadd [85] | YES | YES | NO | YES | NO | NO | NA | NA | YES | NO | YES | NA | NA | NA | GOOD |
|  | Staud et al [86] | YES | YES | NO | YES | YES | NO | NA | NA | YES | NO | YES | NA | NA | NA | GOOD |
|  | Wang et al [87] | YES | YES | YES | YES | YES | NO | NA | NA | YES | NO | YES | NA | NA | NA | GOOD |
| **2020** | Ahmad et al [88] | YES | YES | NA | YES | YES | NO | NO | NA | YES | NO | YES | NA | NA | YES | GOOD |
|  | Karveleas et al [89] | YES | YES | YES | YES | YES | NO | NA | NA | YES | NO | YES | NA | NA | NA | GOOD |
|  | Low et al [90] | YES | YES | NO | YES | NR | NA | NA | NA | YES | NA | YES | NA | NA | NA | GOOD |

*CD, cannot determine; NA, not applicable; NR, not reported

References:

1. Critical Appraisal Skills Programme (CASP) Qualitative Research Checklist [online]. URL: https://casp-uk.net/wp-content/uploads/2018/03/CASP-Qualitative-Checklist-2018_fillable_form.pdf [accessed Apr 27, 2019].
2. George DR, Navarro AM, Stazyk KK, Clark MA, Green MJ. Ethical quandaries and Facebook use: How do medical students think they (and their peers) should (and would) act? AJOB Empir Bioeth. 2014;5(2):68–79. doi: 10.1080/23294515.2013.864344.
3. Henry RK, Pieren JA. The use of social media in dental hygiene programs: a survey of program directors. J Dent Hyg. 2014 Aug;88(4):243–9. PMID: 25134957
4. Langenfeld SJ, Cook G, Sudbeck C, Luers T, Schenarts PJ. An assessment of unprofessional behavior among surgical residents on Facebook: a warning of the dangers of social media. J Surg Educ. 2014 Dec;71(6):e28-32. PMID: 24981657 doi: 10.1016/j.jsurg.2014.05.013
5. Ness GL, Sheehan AH, Snyder ME. Graduating student pharmacists’ perspectives on e-professionalism and social media: qualitative findings. J Am Pharm Assoc (2003). 2014 Mar-Apr;54(2):138–43. PMID: 24632929 doi: 10.1331/JAPhA.2014.13188
6. Chretien KC, Tuck MG, Simon M, Singh LO, Kind T. A Digital Ethnography of Medical Students who Use Twitter for Professional Development. J Gen Intern Med. 2015 Nov;30(11):1673–80. PMID: 25952652 doi: 10.1007/s11606-015-3345-z
7. Jawaid M, Khan MH, Bhutto SN. Social network utilization (Facebook) & e-Professionalism among medical students. Pak J Med Sci. 2015 Jan-Feb;31(1):209-13. PMID: 25878645 doi: 10.12669/pjms.311.5643
8. Flickinger TE, O’Hagan T, Chisolm MS. Developing a Curriculum to Promote Professionalism for Medical Students Using Social Media: Pilot of a Workshop and Blog-Based Intervention. JMIR Med Educ. 2015 Dec 1;1(2):e17. PMID: 27731846 doi: 10.2196/mededu.4886
9. Langenfeld SJ, Sudbeck C, Luers T, Adamson P, Cook G, Schenarts PJ. The Glass Houses of Attending Surgeons: An Assessment of Unprofessional Behavior on Facebook Among Practicing Surgeons. J Surg Educ. 2015 Nov-Dec;72(6):e280-5. PMID: 26276300 doi: 10.1016/j.jsurg.2015.07.007
10. Khandelwal A, Nugus P, Elkoushy MA, Cruess RL, Cruess SR, Smilovitch M, et al. How we made professionalism relevant to twenty-first century residents. Med Teach. 2015;37(6):538–42. PMID: 25594336 doi: 10.3109/0142159X.2014.990878
11. Marnocha S, Marnocha MR, Pilliow T. Unprofessional content posted online among nursing students. Nurse Educ. 2015 May-Jun;40(3):119–23. PMID: 25501656 doi: 10.1097/NNE.0000000000000123
12. Mawdsley A. Pharmacy students’ perceptions of social media in education. Pharmacy Education. 2015;15(1):108–10.
13. Nyangeni T, Du Rand S, Van Rooyen D. Perceptions of nursing students regarding responsible use of social media in the Eastern Cape. Curationis. 2015 Jul 23;38(2):1496. PMID: 26244464 doi: 10.4102/curationis.v38i2.1496
14. Pereira I, Cunningham AM, Moreau K, Sherbino J, Jalali A. Thou shalt not tweet unprofessionally: an appreciative inquiry into the professional use of social media. Postgrad Med J. 2015 Oct;91(1080):561–4. PMID: 26294333 doi: 10.1136/postgradmedj-2015-133353
15. Campbell L, Evans Y, Pumper M, Moreno MA. Social media use by physicians: a qualitative study of the new frontier of medicine. BMC Med Inform Decis Mak. 2016 Jul 15;16:91. PMID: 27418201 doi: 10.1186/s12911-016-0327-y
16. Ferguson C, DiGiacomo M, Saliba B, Green J, Moorley C, Wyllie A et al. First year nursing students’ experiences of social media during the transition to university: a focus group study. Contemp Nurse. 2016 Oct;52(5):625–35. PMID: 27334672 doi: 10.1080/10376178.2016.1205458
17. Panahi S, Watson J, Partridge H. Fostering interpersonal trust on social media: physicians’ perspectives and experiences. Postgrad Med J. 2016 Feb;92(1084):70–3. PMID: 26627975 doi:10.1136/postgradmedj-2015-133270
18. Benetoli A, Chen TF, Schaefer M, Chaar B, Aslani P. Pharmacists’ perceptions of professionalism on social networking sites. Res Social Adm Pharm. 2017 May-Jun;13(3):575–88. PMID: 27423783 doi: 10.1016/j.sapharm.2016.05.044
19. Chester AN, Walthert SE, Gallagher SJ, Anderson LC, Stitely ML. Patient-targeted Googling and social media: a cross-sectional study of senior medical students. BMC Med Ethics. 2017 Dec 4;18(1):70. PMID:29202840 doi: 10.1186/s12910-017-0230-9
20. Duke VJA, Anstey A, Carter S, Gosse N, Hutchens KM, Marsh JA. Social media in nurse education: Utilization and E-professionalism. Nurse Educ Today. 2017 Oct;57:8–13. PMID: 28683342 doi: 10.1016/j.nedt.2017.06.009
21. Gomes AW, Butera G, Chretien KC, Kind T. The Development and Impact of a Social Media and Professionalism Course for Medical Students. Teach Learn Med. 2017 Jul-Sep;29(3):296–303. PMID: 28272900 doi: 10.1080/10401334.2016.1275971
22. Henning MA, Hawken S, MacDonald J, McKimm J, Brown M, Moriarty H, et al. Exploring educational interventions to facilitate health professional students’ professionally safe online presence. Med Teach. 2017 Sep;39(9):959-66. PMID: 28562145 doi: 10.1080/0142159X.2017.1332363
23. Jafarey A, Shekhani S, Mohsin-E-Azam, Gill R, Shirazi B, Hassan M, et al. Physicians in Cyberspace: Finding Boundaries. Asian Bioeth Rev. 2016 Dec;8(4):272–89. doi: 10.1353/asb.2016.0023
24. Koo K, Ficko Z, Gormley EA. Unprofessional content on Facebook accounts of US urology residency graduates. BJU Int. 2017 Jun;119(6):955–60. PMID: 28393475 doi: 10.1111/bju.13846
25. Neville P. Social media and professionalism: a retrospective content analysis of Fitness to Practise cases heard by the GDC concerning social media complaints. Br Dent J. 2017 Sep 8;223(5):353–7. PMID: 28883584 doi: 10.1038/sj.bdj.2017.765
26. Nicolai L, Schmidbauer M, Gradel M, Ferch S, Antón S, Hoppe B, et al. Facebook Groups as a Powerful and Dynamic Tool in Medical Education: Mixed-Method Study. J Med Internet Res. 2017 Dec 22;19(12):e408. PMID: 29273572 doi: 10.2196/jmir.7990
27. Scragg B, Shaikh S, Shires G, Stein Hodgins J, Mercer C, Robinson L, et al. An exploration of mammographers’ attitudes towards the use of social media for providing breast screening information to clients. Radiography (Lond). 2017 Aug;23(3):249–55. PMID: 28687294 doi: 10.1016/j.radi.2017.04.004
28. Soares W, Shenvi C, Waller N, Johnson R, Hodgson CS. Perceptions of Unprofessional Social Media Behavior Among Emergency Medicine Physicians. J Grad Med Educ. 2017 Feb;9(1):85–9. PMID: 28261400 doi: 10.4300/JGME-D-16-00203.1
29. Knott PN, Wassif HS. Older and wiser? First year BDS graduate entry students and their views on using social media and professional practice. Br Dent J. 2018 Sep;225(5):437–40. PMID: 30168814 doi: 10.1038/sj.bdj.2018.745
30. Koo K, Bowman MS, Ficko Z, Gormley EA. Older and wiser? Changes in unprofessional content on urologists’ social media after transition from residency to practice. BJU Int. 2018 Aug;122(2):337–43. PMID: 29694713 doi: 10.1111/bju.14363
31. Nason KN, Byrne H, Nason GJ, O’Connell B. An assessment of professionalism on students’ Facebook profiles. Eur J Dent Educ. 2018 Feb;22(1):30–3. PMID: 27735108 doi: 10.1111/eje.12240
32. Al Qarni AM, Al Shehri SZ, Wahab MMA. Perceptions and Attitudes of Family Medicine Residency Program Directors Regarding Ranking Applicants for Residency Programs using their Social Media Accounts: A National Study in Saudi Arabia. J Fam Community Med. 2019 Aug;26(2):133–40. PMID:31143087 doi: 10.4103/jfcm.JFCM_176_18
33. Hsieh J-G, Kuo L-C, Wang Y-W. Learning medical professionalism - the application of appreciative inquiry and social media. Med Educ Online. 2019 Dec;24(1):1586507. PMID:30831060 doi: 10.1080/10872981.2019.1586507
34. Justinia T, Alyami A, Al-Qahtani S, Bashanfar M, El-Khatib M, Yahya A, Zagzoog F. Social Media and the Orthopaedic Surgeon: a Mixed Methods Study. Acta Inform Med. 2019 Mar;27(1):23–8. PMID:31213739 doi: 10.5455/aim.2019.27.23-28.
35. Loo ME, Wong B, Lee YM. Evaluating the appropriateness of facebook posts – what do faculty and residents consider? The Asia Pacific Scholar. 2020;5(3):71–82. doi: 10.29060/TAPS.2020-5-3/OA2226
36. Kerr H, Booth R, Jackson K. Exploring the Characteristics and Behaviors of Nurses Who Have Attained Microcelebrity Status on Instagram: Content Analysis. J Med Internet Res. 2020 May 26;22(5):e16540. PMID:32452809 doi: 10.2196/16540
37. Ruan B, Yilmaz Y, Lu D, Lee M, Chan TM. Defining the Digital Self: A Qualitative Study to Explore the Digital Component of Professional Identity in the Health Professions. J Med Internet Res. 2020 Sep 29;22(9):e21416. PMID: 32990636 doi: 10.2196/21416
38. National Heart, Lung, and Blood Institute. 2014. Quality Assessment Tool for Observational Cohort and Cross-Sectional Studies - NHLBI, NIH. URL: https://www.nhlbi.nih.gov/health-pro/guidelines/in-develop/cardiovascular-risk-reduction/tools/cohor t [accessed Apr 19, 2019].
39. Bagley JE, DiGiacinto D, Lawyer J, Anderson MP. Health Care Students Who Frequently Use Facebook Are Unaware of the Risks for Violating HIPAA Standards: A Pilot Study. J Diagn Med Sonogr. 2014;30(3):114–20. doi: 10.1177/8756479314530509
40. Levati S. Professional conduct among registered nurses in the use of online social networking sites. J Adv Nurs. 2014 Oct;70(10):2284–92. PMID: 24617801 doi: 10.1111/jan.12377
41. Rocha PN, de Castro NA. Opinions of students from a Brazilian medical school regarding online professionalism. J Gen Intern Med. 2014 May;29(5):758–64. PMID: 24395103 doi: 10.1007/s11606-013-2748-y
42. Alkhateeb F, Alameddine S, Attarabeen O, Latif DA, Osolin S, et al. Pharmacy students’ use of social media sites and perception toward Facebook use. Archives of Pharmacy Practice. 2015;6(4):77-84. doi: 10.4103/2045-080X.165134
43. Avcı K, Çelikden SG, Eren S, Aydenizöz D. Assessment of medical students’ attitudes on social media use in medicine: a cross-sectional study. BMC Med Educ. 2015 Feb 15;15(1):18. PMID: 25890252 doi: 10.1186/s12909-015-0300-y
44. Barlow CJ, Morrison S, Stephens HO, Jenkins E, Bailey MJ, Pilcher D. Unprofessional behaviour on social media by medical students. Med J Aust. 2015 Dec 14;203(11):439. PMID: 26654611
45. Brisson GE, Fisher MJ, LaBelle MW, Kozmic SE. Defining a mismatch: differences in usage of social networking sites between medical students and the faculty who teach them. Teach Learn Med. 2015;27(2):208–14. PMID: 25893945 doi: 10.1080/10401334.2015.1011648
46. Borgmann H, DeWitt S, Tsaur I, Haferkamp A, Loeb S. Novel survey disseminated through Twitter supports its utility for networking, disseminating research, advocacy, clinical practice and other professional goals. Can Urol Assoc J. 2015 Oct;9(9–10):E713-7. PMID:26664662 doi: 10.5489/cuaj.3014
47. Gupta S, Singh S, Dhaliwal U. Visible Facebook profiles and e-professionalism in undergraduate medical students in India. J Educ Eval Health Prof. 2015;12:50. PMID:26582630 doi: 10.3352/jeehp.2015.12.50
48. Fuoco M, Leveridge MJ. Early adopters or laggards? Attitudes toward and use of social media among urologists: Urologists’ use of social media. BJU Int. 2015 Mar;115(3):491–7. PMID: 24981237 doi: 10.1111/bju.12855

49. Klee D, Covey C, Zhong L. Social media beliefs and usage among family medicine residents and practicing family physicians. Fam Med. 2015 Mar;47(3):222-6. PMID: 25853534

50. Mohiuddin Z, Shahid H, Shuaib W. Social Media Impact: Utility of Reflective Approach in the Practice of Surgery. Indian J Surg. 2015 Dec;77(Suppl 3):893–8. PMID: 27011477 doi: 10.1007/s12262-014-1056-z

51. Walton JM, White J, Ross S. What’s on YOUR Facebook profile? Evaluation of an educational intervention to promote appropriate use of privacy settings by medical students on social networking sites. Med Educ Online. 2015 Jul 20;20:28708. PMID: 26198434 doi: 10.3402/meo.v20.28708

52. Adilman R, Rajmohan Y, Brooks E, Urgoiti GR, Chung C, Hammad N, et al. Social Media Use Among Physicians and Trainees: Results of a National Medical Oncology Physician Survey. J Oncol Pract. 2016 Jan;12(1):79–80, e52-60. PMID: 26443837 doi: 10.1200/JOP.2015.006429

53. Garg M, Pearson DA, Bond MC, Runyon M, Pillow MT, Hopson L, et al. Survey of Individual and Institutional Risk Associated with the Use of Social Media. West J Emerg Med. 2016 May;17(3):344–9. PMID: 27330669 doi: 10.5811/westjem.2016.2.28451

54. Gettig JP, Noronha S, Graneto J, Obucina L, Christensen KJ, Fjortoft NF. Examining Health Care Students’ Attitudes toward E-Professionalism. Am J Pharm Educ. 2016 Dec 25;80(10):169. PMID:28179718 doi: 10.5688/ajpe8010169

55. Kenny P, Johnson IG. Social media use, attitudes, behaviours and perceptions of online professionalism amongst dental students. Br Dent J. 2016 Nov 18;221(10):651–5. PMID: 27857111 doi: 10.1038/sj.bdj.2016.864

56. Kesselheim JC, Schwartz A, Belmonte F, Boland KA, Poynter S, Batra M, et al. A National Survey of Pediatric Residents’ Professionalism and Social Networking: Implications for Curriculum Development. Acad Pediatr. 2016 Mar;16(2):110–4. PMID: 26718877 doi:10.1016/j.acap.2015.12.004

57. Kitsis EA, Milan FB, Cohen HW, Myers D, Herron P, McEvoy M, et al. Who’s misbehaving? Perceptions of unprofessional social media use by medical students and faculty. BMC Med Educ. 2016 Feb 18;16:67. PMID: 26887561 doi: 10.1186/s12909-016-0572-x

58. Langenfeld SJ, Vargo DJ, Schenarts PJ. Balancing Privacy and Professionalism: A Survey of General Surgery Program Directors on Social Media and Surgical Education. J Surg Educ. 2016 Nov - Dec;73(6):e28–e32. PMID: 27524278 doi: 10.1016/j.jsurg.2016.07.010

59. Lefebvre C, Mesner J, Stopyra J, O’Neill J, Husain I, Geer C, et al. Social Media in Professional Medicine: New Resident Perceptions and Practices. J Med Internet Res. 2016 Jun 9;18(6):e119. PMID: 27283846 doi: 10.2196/jmir.5612

60. Mather C, Cummings E, Nichols L. Social Media Training for Professional Identity Development in Undergraduate Nurses. Stud Health Technol Inform. 2016;225:344–8. PMID: 27332219

61. Nikiphorou E, Studenic P, Ammitzbøll CG, Canavan M, Jani M, Ospelt C, Berenbaum F, EMEUNET. Social media use among young rheumatologists and basic scientists: results of an international survey by the Emerging EULAR Network (EMEUNET). Ann Rheum Dis. 2017 Apr;76(4):712–5. PMID:27797750 doi: 10.1136/annrheumdis-2016-209718

62. Robertson M, Shoss MK, Broom MA. Social Media: Social Intelligence Training Module. MedEdPORTAL. 2016 Aug 26;12:10442. PMID:31008220 doi: 10.15766/mep_2374-8265.10442

63. Yang Y-M, Jeong E, Je NK, Jee J-P, Yoo JC, Choi EJ. An analysis of pharmacy students’ social networking service activities and perceptions regarding e-professionalism under the newly implemented 6-year pharmacy educational system in South Korea. Indian Journal of Pharmaceutical Education and Research. 2016;50(1):63–9. doi: 10.5530/ijper.50.1.9

64. Dawkins R, King WD, Boateng B, Nichols M, Desselle BC. Pediatric Residents’ Perceptions of Potential Professionalism Violations on Social Media: A US National Survey. JMIR Med Educ. 2017 Jan 31;3(1):e2. PMID: 28143804 doi: 10.2196/mededu.5993

65. D’Souza K, Henningham L, Zou R, Huang J, O’Sullivan E, Last J, et al. Attitudes of Health Professional Educators Toward the Use of Social Media as a Teaching Tool: Global Cross-Sectional Study. JMIR Med Educ. 2017 Aug 4;3(2):e13. PMID: 28778841 doi: 10.2196/mededu.6429

66. Duymuş TM, Karadeniz H, Şükür E, Atiç R, Zehir S, Azboy İ. Social media and Internet usage of orthopaedic surgeons. J Clin Orthop Trauma. 2017;8(1):25–30. PMID:28360492 doi: 10.1016/j.jcot.2016.10.007

67. Laliberté M, Beaulieu-Poulin C, Campeau Larrivée A, Charbonneau M, Samson É, Ehrmann Feldman D. Current Uses (and Potential Misuses) of Facebook: An Online Survey in Physiotherapy. Physiother Can. 2016;68(1):5–12. PMID: 27504042 doi: 10.3138/ptc.2014-41

68. Long X, Qi L, Ou Z, Zu X, Cao Z, Zeng X, Li Y, Chen M, Wang Z, Wang L. Evolving use of social media among Chinese urologists: Opportunity or challenge? PLOS One. 2017 Jul 28;12(7):e0181895. PMID: **28753632** doi: 10.1371/journal.pone.0181895

69. Marnocha S, Marnocha M, Cleveland R, Lambie C, Limberg CY, Wnuk J. A Peer-Delivered Educational Intervention to Improve Nursing Student Cyberprofessionalism. Nurse Educ. 2017 Oct;42(5):245–9. PMID: 28252547 doi: 10.1097/NNE.0000000000000368

70. Mostaghimi A, Olszewski AE, Bell SK, Roberts DH, Crotty BH. Erosion of Digital Professionalism During Medical Students’ Core Clinical Clerkships. JMIR Med Educ. 2017 May 3;3(1):e9. PMID: 28468745 doi: 10.2196/mededu.6879

71. O’Sullivan E, Cutts E, Kavikondala S, Salcedo A, D’Souza K, Hernandez-Torre M, Anderson C, Tiwari A, Ho K, Last J. Social Media in Health Science Education: An International Survey. JMIR Med Educ 2017 Jan 4;3(1):e1. PMID:28052842

72. Patel SS, Hawkins CM, Rawson JV, Hoang JK. Professional Social Networking in Radiology: Who Is There and What Are They Doing? Acad Radiol. 2017 May;24(5):574–9. PMID: 28153576 doi: 10.1016/j.acra.2016.09.026

73. Surani Z, Hirani R, Elias A, Quisenberry L, Varon J, Surani S, et al. Social media usage among health care providers. BMC Res Notes. 2017 Nov 29;10(1):654. PMID: 29187244 doi: 10.1186/s13104-017-2993-y

74. Wagner JP, Cochran AL, Jones C, Gusani NJ, Varghese TK Jr, Attai DJ. Professional Use of Social Media Among Surgeons: Results of a Multi-Institutional Study. J Surg Educ. 2018 May-Jun;75(3):804–10. PMID: 28964746 doi: 10.1016/j.jsurg.2017.09.008

75. Weijs C, Majowicz S, Coe JB, Desmarais S, Jones-Bitton A. The personal use of Facebook by public health professionals in Canada: Implications for public health practice. J Commun Healthc. 2017 Jan 2;10(1):8–15. doi: 10.1080/17538068.2016.1274846

76. Alshakhs F, Alanzi T. The evolving role of social media in health-care delivery: measuring the perception of health-care professionals in Eastern Saudi Arabia. J Multidiscip Healthc. 2018 Sep 21;11:473-79. PMID: 30275699 doi: 10.2147/JMDH.S171538

77. Barnable A, Cunning G, Parcon M. Nursing Students’ Perceptions of Confidentiality, Accountability, and E-Professionalism in Relation to Facebook. Nurse Educ. 2018 Jan/Feb;43(1):28–31. PMID: 28857955 doi: 10.1097/NNE.0000000000000441

78. Hazzam J, Lahrech A. Health Care Professionals’ Social Media Behavior and the Underlying Factors of Social Media Adoption and Use: Quantitative Study. J Med Internet Res. 2018 Nov 7;20(11):e12035. PMID:30404773. doi: 10.2196/12035

79. Hinojo-Lucena F-J, Aznar-Díaz I, Cáceres-Reche M-P, Romero-Rodríguez J-M. Use of social networks for international collaboration among medical students. Educacion Médica. 2020 Mar 1;21(2):137–41. doi: 10.1016/j.edumed.2018.08.009

80. Irfan KS, Farhana I, Eiad AF, Nassr AM, Al Mohammed AQ, Maya N, et al. Family physicians’ utility of social media: a survey comparison among family medicine residents and physicians. Afr Health Sci. 2018 Sep;18(3):817-27. PMID: 30603016 doi: 10.4314/ahs.v18i3.41

81. West CA, Wagner JM, Greenberg SB, Buck E, Hsieh P, Horn K, et al. Examining Medical Students’ Social Media Beliefs and Behaviors and Their Relationship to Professional Identity. Medical Science Educator. 2018;28(2):389–99. doi: 10.1007/s40670-018-0562-1

82. Dobson E, Patel P, Neville P. Perceptions of e-professionalism among dental students: a UK dental school study. Br Dent J. 2019 Jan;226(1):73–8. PMID: **30631197** doi: 10.1038/sj.bdj.2019.11

83. Lee YJ, Hwang J, Lee SI, Woo S-I, Hahn SW, Koh S. Impact of experience of psychiatrists and psychiatry residents regarding electronic communication and social networking on internet use patterns: a questionnaire survey for developing e-professionalism in South Korea. BMC Med Educ. 2019 Nov 8;19(1):411. PMID:31703677 doi: 10.1186/s12909-019-1771-z

84. Renew JR, Ladlie B, Gorlin A, Long T. The Impact of Social Media on Anesthesia Resident Recruitment. J Educ Perioper Med. 2019 Mar;21(1):E632. PMID:31406704

85. Sadd R. Student Nurse Attitudes and Behaviours when Using Social Network Sites. Stud Health Technol Inform. 2019 Aug 21;264:1342–6. PMID:31438144 doi: 10.3233/SHTI190445

86. Staud SN, Kearney RC. Social Media Use Behaviors and State Dental Licensing Boards. J Dent Hyg. 2019 Jun;93(3):37–43. PMID:31182567

87. Wang Z, Wang S, Zhang Y, Jiang X. Social media usage and online professionalism among registered nurses: A cross-sectional survey. Int J Nurs Stud. 2019 Oct;98:19–26. PMID:31255853 doi: 10.1016/j.ijnurstu.2019.06.001

88. Ahmad T, Sattar K, Akram A. Medical professionalism videos on YouTube: Content exploration and appraisal of user engagement. Saudi J Biol Sci. 2020 Sep;27(9):2287–92. PMID:32884409 doi: 10.1016/j.sjbs.2020.06.007

89. Karveleas I, Kyriakouli A, Koukou M, Koufatzidou M, Kalogirou E-M, Tosios KI. The relationship between Facebook behaviour and e-professionalism: A questionnaire-based cross-sectional study among Greek dental students. Eur J Dent Educ. 2020 Aug 11; PMID:32780448 doi: 10.1111/eje.12585

90. Low J, Tan M, Joseph R. Doctors and social media: knowledge gaps and unsafe practices. Singapore Med J. 2020 Apr 21. PMID: **32312027 doi:** 10.11622/smedj.2020067
